# A Comparative Study of Some Procedures for Isolation of Fruit DNA of Sufficient Quality for PCR-Based Assays

**DOI:** 10.3390/molecules25184317

**Published:** 2020-09-20

**Authors:** Lenka Fialova, Denisa Romanovska, Ivana Marova

**Affiliations:** Faculty of Chemistry, Brno University of Technology, 612 00 Brno, Czech Republic; xcromanovska@fch.vut.cz (D.R.); marova@fch.vut.cz (I.M.)

**Keywords:** DNA isolation, real-time PCR, commercial kit, red fruit, *Prunus*, tropical fruit

## Abstract

Food fraud has been and still is a problem in the food industry. It is detectable by several approaches, such as high performance liquid chromatography (HPLC), chemometric assays, or DNA-based techniques, each with its own drawbacks. This work addresses one major drawback of DNA-based methods, in particular their sensitivity to inhibitors contained in particular matrices from which DNA is isolated. We tested five commercial kits and one in-house method characterized by different ways of sample homogenization and DNA capture and purification. Using these methods, DNA was isolated from 10 different fruit species commonly used in plant-based foodstuffs. The quality of the DNA was evaluated by UV-VIS spectrophotometry. Two types of qPCR assays were used for DNA quality testing: (i) Method specific for plant ITS2 region, (ii) methods specific for individual fruit species. Based mainly on the results of real-time PCR assays, we were able to find two column-based kits and one magnetic carrier-based kit, which consistently provided fruit DNA isolates of sufficient quality for PCR-based assays useful for routine analysis and identification of individual fruit species in food products.

## 1. Introduction

Food fraud, both intentional, such as the substitution of an expensive fruit for a cheaper one, and unintentional, such as contamination of a product with undeclared fruit species through insufficient cleaning of processing lines, has been, and still is, a problem in food industry [1,2]. Examples of food fraud detectable by PCR-based techniques include the two types of food fraud mentioned above. There are already established methods for detection of these types of food adulteration in fruit juices and purees, such as HPLC or chemometric assays. Nevertheless, these methods have some limitations due to different content of some metabolites between varieties of the same fruit species and influence of the type of processing and storage which plant-based foodstuffs undergo [1,2,3]. Due to this fact, it still makes sense to also explore PCR-based approaches due to their high specificity, and thermal stability of DNA compared to other compounds present in plant-based food [4].

While PCR-based methods do not suffer from the same drawbacks mentioned in the previous paragraph, they also exhibit some disadvantages. Similar to other enzyme-based procedures, they are sensitive to inhibitors present in biological matrixes. Further, for reliable results, DNA of sufficient concentration and purity is required [5]. However, food, and fruit especially, are rich in substances, which may inhibit PCR [5,6]. Polysaccharides such as pectin can co-precipitate with DNA during its purification and hamper its resuspension. After DNA resuspension, polysaccharides can mimic its properties and hamper PCR. Polyphenols are able to form cross-links with nucleic acids, thus changing their properties and making them incapable of amplification. Phenolics also chelate metal ions, including magnesium ions, which can also lead to the inhibition of amplification [5,6,7]. The conditions during food processing also influence the quality of the DNA isolated from the food matrix. For example, at acidic pH, purines are hydrolyzed from DNA strands. This leads to the hydrolysis of the nearest phosphodiester bond, which in turn leads to breaking of DNA strands into shorter fragments [6].

The goal of present study is to compare several protocols for isolation of DNA from different mature fruit matrixes to obtain DNA of sufficient quality for PCR-based methods. Previously, some studies comparing DNA isolation kits and in-house protocols have already been done. Nevertheless, these studies were focused either on bacterial DNA from clinical samples [8], soil [9], wine and vinegar [10], or plant DNA from matrices other than mature fruit, such as soy products [11] or processed tomato products [6]. To our best knowledge, similar studies using mature fruits of different species and varying chemical compositions have not been done so far. In the present study, five commercial kits and one in-house protocol were selected and compared. These procedures differed in ways of sample homogenization, DNA isolation, and purification. In order to compare the whole DNA isolation protocols instead of only their purification stages, different homogenization steps recommended by the different manufacturers were employed. For the testing of these isolation and purification protocols, 10 species of fruit commonly used in plant-based food and food for infants were used. For real-time PCR assays, intercalating dye was used. To offset its main disadvantage, assays specific for individual fruit species coupled with melting curve analyses were employed. This work is considered as a necessary step for the development of complex PCR-based methods for routine authenticity analysis of fruit purees and juices. Since in these methods the chosen way of amplicon detection will be intercalating dye, we also used it in this study.

## 2. Results

The experimental strategy was proposed as follows:

(i) Isolation of DNA by 5 commercial and 1 in-house method.
(ii) Determination of concentration and purity of DNA by UV-VIS spectrophotometry.
(iii) Analysis of quality of each DNA isolate by real-time PCR using primers specific for ITS2 region of plants.
(iv) Analysis of the quality of each DNA isolate by real-time PCR using specific primers for individual fruit species. Every amplicon was analyzed by agarose gel electrophoresis as well.

A workflow chart of this study can also be found in file S15.

### 2.1. Characterization of DNA Isolates by UV-VIS Spectrophotometry

The purity of DNA isolates was assessed based on the absorbance ratios A260/A280 and A260/A230. For the former, the value 1.8 indicates pure DNA, lower value indicates protein contamination, and value higher than 2.0 indicates RNA contamination. The latter absorbance ratio should be higher than 1.5 and, ideally, close or equal to 1.8 [5]. Table 1 summarizes concentration and purity of DNA isolated from each individual fruit species.

In the case of banana, the cetyltrimethylammonium bromide (CTAB) protocol and kit 3 (Invitrogen) provided the purest samples (Table 1), however, the absorption spectra of the DNA isolates obtained by the CTAB protocol showed a distinctive peak at 270 nm, which indicates possible polysaccharide contamination. This peak was determined in all fruit DNA isolates obtained by the CTAB protocol, but not in DNA isolates obtained by commercial kits. The performance of kit 3 was followed by kit 2 (Elisabeth Pharmacon), which yielded DNA, whose A260/A280 ratio does not indicate any protein contamination, while the A260/A230 indicates possible contamination with residues of salts, EDTA, or phenolic compounds (Table 1). Performances of kits 1 (Qiagen), 4 (PerkinElmer), and 5 (Tools) were comparable.

The bilberry DNA isolates provided by CTAB protocol had the best absorbance ratios. While the A260/A280 ratio indicates some protein contamination, the A260/A230 ratio is higher than 1.5, which was not achieved by any of the other five methods. Kits 2 and 5 provided DNA with slight or no protein contamination, respectively. For pear DNA isolates, the best A260/A280 ratio was measured in isolates provided by kit 5. Using kit 5 for apple DNA isolation, the lowest level of protein contamination was obtained, while the highest was found in isolates obtained by kits 1 and 4. The A260/A230 ratios indicate possible salt contamination in DNA isolates obtained by all methods, with the level of contamination being comparable between the five commercial kits, and significantly lower in case of the CTAB protocol (Table 1).

Regarding DNA isolates from strawberry and raspberry, the best A260/A280 ratio was observed in isolates provided by kits 2 and 5, and by the CTAB protocol. This ratio for kits 1, 3, and 4 indicates protein contamination. The A260/A230 ratio indicated pure DNA in isolates obtained by kit 5 and the CTAB protocol, and the ratio for kit 2 indicates salt or phenolic contamination. (Table 1).

Regarding apricot DNA, the A260/A280 ratio indicated comparable levels of protein contamination in DNA isolates obtained by all methods except kit 5. The A260/A230 ratio of all DNA isolates except those obtained by the CTAB protocol indicated salt/phenolic contamination, which was comparable among all five commercial kits (Table 1).

Of plum DNA isolates, those obtained by kit 2 showed possible RNA contamination. Isolates obtained by all six methods showed salt/phenolic contamination, whose levels were comparable among the five commercial kits, and lower in isolates obtained by the CTAB protocol (Table 1).

Peach DNA isolates obtained by kit 5 were pure, while those obtained by kit 2 showed possible RNA contamination, and those obtained by the CTAB protocol showed slight protein contamination. The rest of the isolates, showed higher, but comparable, levels of protein contamination and salt/phenolic contamination. In DNA isolates obtained by kits 2 and 5, and by the CTAB protocol, the A260/A230 ratio was close to or higher than 1.5, indicating pure DNA (Table 1).

Regarding mango DNA isolates, those obtained by kit 2 showed RNA contamination, while in the other isolates, low levels of protein contamination (kits 3 and 4, CTAB protocol), or pure DNA (kits 1 and 5) was found. The A260/A230 ratios of DNA isolates obtained by kits 1, 4, and 5 were lower than 1.0, indicating salt/phenolic contamination. In isolates obtained by kits 2 and 3, and by the CTAB protocol, the A260/230 ratio, indicates only slight contamination or pure DNA, respectively (Table 1).

To sum up, when assessed only by purity taken as the primary consideration and concentration as the secondary consideration, the best performances overall were given by kits 2 (Elisabeth Pharmacon) and 5 (Tools), and by the CTAB protocol. However, when the duration and complexity of the isolation methods is taken into account, the CTAB protocol must be disqualified as too complicated and time consuming (approximately 6 h of work even with less than five samples) when compared with the two kits (less than 3 h work even with large number of samples).

Another criterion, which helped to determine the final choice of the DNA isolation method most appropriate for our stated purpose was the amplifiability of DNA obtained by each method. As mentioned above, the amplifiability of every DNA isolate was assessed in two types of real-time PCR assays, the results of which are presented in the following chapter. As the final goal, we would like to find simple and reproducible DNA isolation procedures for PCR-based techniques applied in authenticity analyses of processed plant foods.

### 2.2. Real-Time PCR

Another criterion, which helped to determine the final choice of the DNA isolation method most appropriate for our stated purpose, was amplifiability of DNA obtained by each method. As mentioned above, the amplifiability of every DNA isolate was assessed in two types of real-time PCR assays: (i) Method specific for plant ITS2 region, (ii) method specific for individual fruit species. Results were divided into some groups according to fruit types, e.g., red fruits, stone fruits, tropical fruits, and pome fruits.

#### 2.2.1. Red Fruits

**Figure 1:**
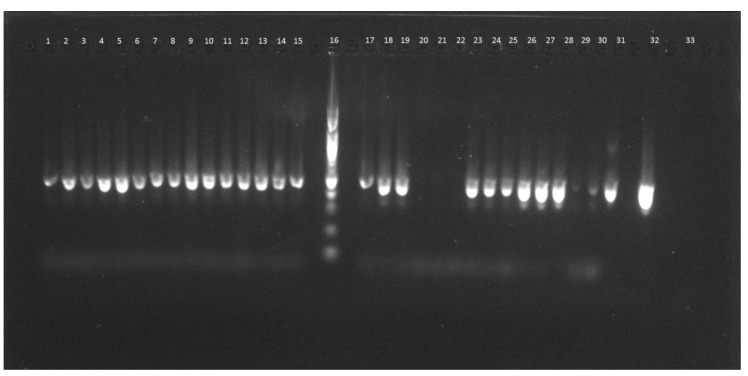
Result of plant-specific PCR assay with DNA isolates obtained by kit 5: 1–3 banana, 4–6 raspberry, 7–9 blueberry, 10–12 mango, 13–15 peach, 16-DNA ladder, 17–19 apricot, 20–22 apple, 23–25 plum, 26–28 strawberry, 29–31 pear, 32 positive control, 33 no template control.

In this study, the various DNA isolation methods were tested on three red fruit species, in particular raspberry, strawberry, and bilberry. Of the raspberry DNA isolates, those obtained by kit 1 (Qiagen), were not amplifiable in either the plant-specific, or the raspberry specific real-time PCR assay (Table 2 and File S10 in Supplementary Materials). All of the other isolates were amplifiable in both assays mentioned above. Best results were achieved with DNA isolates obtained by kits 2 and 4 (Elisabeth Pharmacon and Perkin-Elmer, respectively), followed by kit 3 (Invitrogen), and kit 5 (Tools) (Figure 1, Table 2 and File S10 in Supplementary Materials). However, based on the Ct values of assays with bilberry and strawberry DNA isolates obtained by kit 3 (Table 1, Table 2), it is evident that in these two cases, the concentration of these isolates was overestimated. The isolates obtained by the CTAB protocol were also amplifiable in both plant-specific and raspberry-specific assays, but the results were less acceptable than results achieved with DNA isolates obtained by the commercial kits (Table 2 and File S10 in Supplementary Materials). The amounts of amplified DNA corresponding with each Ct value are in supplementary File S14.

In case of strawberry, all methods, including the CTAB protocol, provided amplifiable DNA isolates. While the results of real-time PCR with plant-specific primers were similar among the six DNA isolation methods (Table 2 and File S9 in Supplementary Materials), with species-specific primers they differed (Table 2 and File S9 in Supplementary Materials). The assays with DNA isolated by kit 5 (Tools) yielded the best results by far (Table 2 and File S9 in Supplementary Materials). Similarly to strawberry DNA, bilberry DNA yielded comparable results in plant specific assays with isolates obtained by all of the commercial kits (Table 2 and File S5 in Supplementary Materials), while the results of bilberry-specific assays once again differed. The best results were achieved with DNA isolates obtained by kit 5 (Tools), followed by kit 3 (Invitrogen) (Table 2 and File S5 in Supplementary Materials). Based on these results, we consider kit 5 (Tools) the most appropriate for DNA isolation from food with high content of red fruit, with kit 3 being a suitable alternative.

#### 2.2.2. Stone Fruits

**Figure 2:**
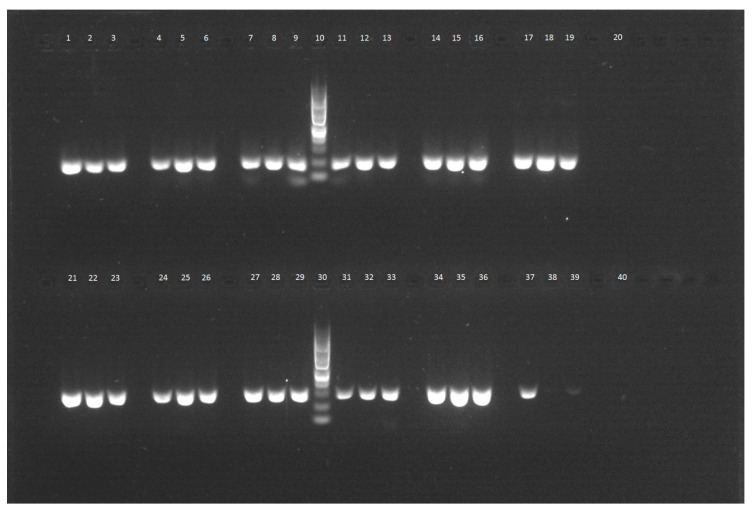
Results of PCR assay with primers, specific for individual species, upper row: Peach, 1–3 kit 1 (Qiagen), 4–6 kit 2 (Elisabeth Pharmacon), 7–9 kit 3 (Invitrogen), 10,100 bp DNA ladder, 11–13 kit 4 (PerkinElmer), 14–16 kit 5 (Tools), 17–19 CTAB protocol, 20 no template control. Lower row: Banana, 21–23 kit 1, 2426 kit 2, 27–29 kit 3, 30 DNA ladder, 31–33 kit 4, 34–36 kit 5, 37–39 CTAB protocol, 40 no template control.

This group of fruits involved one of the least problematic samples in this study (peach) and two samples which proved to be recalcitrant (apricot and plum). In the case of peach, good results were achieved with DNA isolates obtained by kit 1 (Qiagen) providing best results in real-time PCR assays with plant-specific primers (Table 3 and File S6 in Supplementary Materials), and kit 5 (Tools) and the CTAB protocol in peach-specific assays (Figure 2, Table 3 and File S6 in Supplementary Materials). All other kits in all preparations provided DNA, which was successfully amplified in both plant-specific and peach-specific assays with only slightly lower PCR efficiency than the DNA obtained by kits 1 and 5 and the CTAB protocol (Table 3 and File S6 in Supplementary Materials). The amounts of amplified DNA which correspond with each Ct value can be found in supplementary File S14, Table 2.

However, in the case of apricot and plum, kit 1 proved to be the least appropriate method for DNA isolation from these species, with no amplification observed in any, or only in one out of three, parallel real-time PCR assays, regardless of primers used (Table 3 and File S12 in Supplementary Materials). Assays with plum DNA isolates obtained by kit 5 yielded the best results, followed by those with DNA isolates obtained by kit 2 (Elisabeth Pharmacon) (Table 3 and File S13 in Supplementary Materials). Plum DNA isolates obtained by kit 4 (Perkin-Elmer) were successfully amplified in assays with plant-specific primers, and so were the DNA isolates obtained by the CTAB protocol, although with a lower PCR efficiency (Table 3 and File S13 in Supplementary Materials). However, the results of plum-specific assay with DNA isolates obtained by kit 4 indicate that the melting temperature of the amplicons differs from the one observed in other plum specific assays with DNA isolates obtained by all other methods (File S13 in Supplementary Materials), even though the DNA was isolated from the same fruit. In plum specific assay with DNA isolates obtained by the CTAB protocol, no specific amplification was observed (Table 3 and File S13 in Supplementary Materials). Regarding kit 3 (Invitrogen), no amplification was observed in assays with plum DNA isolates obtained by this kit, regardless of primers used (Table 3 and File S13 in Supplementary Materials). 

In the case of apricot, the best results were achieved with DNA isolates obtained by kit 3, followed by kit 5 (Table 3 and File S12 in Supplementary Materials). Kits 2 and 4 provided amplifiable DNA in two out of three parallel preparations, with the PCR efficiency being comparable between these two kits (Table 3 and File S12 in Supplementary Materials). In assays with DNA isolates obtained by the CTAB protocol, specific amplicons were either not detected (plant-specific assays), or they were detected only in one out of three parallels (apricot-specific assays) (Table 3 and File S12 in Supplementary Materials). To sum up, kit 5 proved to be the most appropriate for DNA isolation from the stone fruit species used in this study, or foodstuffs with high content of plum, apricot, or peach matrix. We propose kit 2 as an alternative to kit 5, as the former also consistently provided amplifiable DNA from all three stone fruit species in this study, although the PCR efficiency was lower. For DNA isolation from apricot, or food with high content of apricot matrix, kit 3 is the most appropriate, although we do not recommend it for DNA isolation from plums.

#### 2.2.3. Tropical Fruit

Two tropical fruit species—banana and mango—were among the least problematic samples in this study. In case of mango, DNA isolates from every preparation were amplifiable in the plant-specific assay, and all DNA isolates except those obtained by kit 2 (Elisabeth Pharmacon) were amplifiable in the mango-specific assay (Table 4 and File S11 in Supplementary Materials). Best results in terms of PCR efficiency were achieved in assays with DNA isolates obtained by kits 1 (Qiagen) and 5 (Tools) (Table 4 and File S11 in Supplementary Materials). The amounts of amplified DNA corresponding with each Ct value can be found in supplementary File S14, Table 3.

In case of banana, the results were similar. Two differences were observed: All DNA isolates obtained by kit 2 were amplifiable in both plant-specific and banana-specific assay, while DNA isolates obtained by the CTAB protocol could be amplified in both assays with much lower PCR efficiency than any of the DNA isolates obtained by the five commercial kits (File S4 in Supplementary Materials). When the results of both plant-specific and fruit-specific assays are taken into account, we can conclude that kits 1 and 5 are the most suitable for DNA isolation from both mango and banana, or from foodstuffs with high content of these two species. For banana, we suggest kits 2 and 3 (Invitrogen) as possible alternatives, and for mango, we suggest kits 3 and 4 as possible alternatives to kits 1 and 5.

#### 2.2.4. Pome Fruit

Apple and pear were the most problematic fruit samples used in this study. However, fruit based infant food is often composed mostly of apple and/or pear puree, so the results of the real-time PCR assays with DNA isolates from these two fruits had a great influence on our final choice of a kit for further work.

In assays with pear DNA isolates, best results were achieved with those obtained by kit 2 (Elisabeth Pharmacon). This kit yielded DNA amplifiable in both plant-specific and pear-specific assays in all three parallel preparations (Table 5 and File S7 in Supplementary Materials). Kits 1 (Qiagen), 3 (Invitrogen), and 5 (Tools) also provided DNA isolates amplifiable in both assays, but the PCR efficiency was lower (Table 5 and File S7 in Supplementary Materials). Kit 4 provided DNA isolates amplifiable in plant-specific assays only, (Table 5 and File S7 in Supplementary Materials). Using the isolates obtained by the CTAB protocol, specific amplicon was determined in one out of three parallels, and only in the plant-specific assay (File S7 in Supplementary Materials). The amounts of amplified DNA corresponding with each Ct value can be found in supplementary File S14, Table 4.

Apple DNA isolates obtained by kit 3 (Invitrogen), were successfully amplified in both plant-specific and apple-specific assays (Table 5 and File S8 in Supplementary Materials). Isolates obtained by the other four commercial methods exhibited specific amplification in at least two out of three parallels in all plant-specific assays (File S8 in Supplementary Materials). However, the PCR efficiency was low, and in the case of kit 5 (Tools) the amplicons were not detectable by electrophoresis (Table 5 and File S3 in Supplementary Materials). Apple-specific amplification was observed only with DNA isolates obtained by kits 2 (Elisabeth Pharmacon) 3, and 5 (File S8 in Supplementary Materials). To sum up, we consider kit 2 (Elisabeth Pharmacon) the most appropriate for DNA isolation from pear, and kit 3 (Invitrogen) the most appropriate for DNA isolation from apple. At the same time, apple DNA isolates obtained by kit 2 were amplifiable, and the same was true for pear DNA isolates obtained by kit 3. We therefore consider these two kits the most appropriate for DNA isolation from apple and pear, or from foodstuffs with high content of either of these fruits.

When considered together, the results of the plant-specific PCR assay and the species-specific PCR assays show that of the five commercial kits tested in this study, the most universal were kit 2 (Elisabeth Pharmacon), kit 3 (Invitrogen), and partially kit 5 (Tools). These kits consistently provided amplifiable DNA from most of the ten fruit species used in this study, and each of them proved to be the most suitable for DNA isolation from some of the more problematic samples, in particular plum, apricot, apple, and pear.

For further work, we can recommend particularly kit 2 (Elisabeth Pharmacon). The deciding factors included the quality of pear and apple DNA isolated with these kits, because apple and/or pear puree is often a major component of infant food, a requirement of liquid nitrogen for sample homogenization, time needed for DNA isolation, and price per sample. The first factor disqualified kit 5 (Tools), which is not recommended for DNA isolation from either apple or pear. The remaining factors disqualified the magnetic carrier-based kit 3 (Invitrogen), as the DNA isolation using the column based kit 2 (Elisabeth Pharmacon) was easier and faster, did not require sample homogenization in liquid nitrogen, and the price per sample was lower. 

## 3. Discussion

In this study, we compared six DNA isolation methods characterized by different ways of sample homogenization and DNA capture and purification. Five of these methods were commercial and one was in-house. The aim of this study was to select/verify a method for DNA isolation from different single fruits, which would provide DNA of sufficient quality for following PCR-based assays. In this work, 10 individual fruit species were tested. The main criteria of evaluation were (i) repeated detection of specific amplicons in real-time PCR assays with both plant-specific and fruit species specific primers, with emphasis placed on the results of assays with apple and pear DNA isolates, (ii) sufficient concentration and purity of isolated DNA, (iii) simplicity or complexity of each method, (iv) price per sample.

Each method showed some differences in performance depending on both the fruit from which the DNA was isolated and primers used in both types of real-time PCR assays—methods specific for plant ITS2 region and methods specific for DNA of individual fruit species. The isolates obtained by the CTAB protocol showed A260/A280 ratios comparable to isolates obtained by the commercial kits, and 260/230 ratios indicative of lower levels of salt/phenolic contamination than in the isolates obtained by the commercial kits. However, the UV absorption spectra of DNA isolates obtained by the CTAB protocol also showed a distinctive peak at 270 nm, which indicated a possible polysaccharide contamination. This peak was not determined in the UV absorption spectra of any DNA isolates obtained by the commercial kits. Further, when evaluated in real-time PCR assays, the CTAB protocol provided poorest results overall, which indicates that the contaminants had inhibitory effect on the PCR assays.

Out of the five commercial methods, the best performance was given by kits 2 (Elisabeth Pharmacon), 3 (Invitrogen), and 5 (Tools). Choosing any of these methods as the sole protocol for DNA isolation from mature fruit means a certain compromise. The most reasonable one, and also our final recommendation, could be to use kit 2. This kit performed better in DNA isolation from either apple or pear yielded DNA isolates of acceptable concentration, and the purity of the DNA isolates also tended to be better in those obtained by kit 2 (Table 1). Furthermore, DNA isolation was faster with kit 2, sample homogenization with liquid nitrogen was not required, unlike with kit 3 (Table 2), and price per sample was lower with kit 2 as well. We therefore found kit 2 to be the most suitable for general use, but we recommend kit 5 for DNA isolation from foodstuffs with high content of fruit matrix other than apple or pear, and kit 3 for DNA isolation from apricot and apple fruit, or from foodstuffs with high content of these two species.

## 4. Materials and Methods

### 4.1. Fruit Species

For this study, ten different fruit species were selected, in particular apple (*Mallus domestica*), pear (*Pyrus communis*), red raspberry (*Rubus idaeus*), bilberry (also known as European blueberry), (*Vaccinium myrtillus*), peach (*Prunus persica*), apricot (*Prunus armeniaca*), plum (*Prunus domestica*), banana (*Musa acuminata*), mango (*Mangifera indica*), and strawberry (*Fragaria ananasa*). Mature fruits of these species were obtained in a commercial retail. The fruits were cut into small pieces with a scalpel and stored in a freezer at −20°C until further use.

### 4.2. Commercial Kits for DNA Isolation

Five kits and one in-house method were selected for the DNA isolation. Different ways of sample homogenization and DNA capture and purification were applied, which are summarized in Table 6.

### 4.3. DNA Isolation

Simple in-house method based on CTAB protocol proposed by Saghai-Maroof, 1987, with modification by Glyn et al., 1997 [12,13], was compared with different commercial kits. Isolation kits EliGene Plant DNA Isolation Kit, Elisabeth Pharmacon, Brno, Czech Republic; Chemagic DNA Plant Kit, Perkin-Elmer, USA; Easy Prep Polyphenol Plant DNA Extraction Kit, Tools, New Taipei City, Taiwan; ChargeSwitch gDNA Plant Kit, Invitrogen, Carlsbad, CA, USA and DNEasy PowerPlant Pro Kit, Qiagen, Hilden, Germany were selected. The DNA isolations by commercial methods were performed according to the manufacturers’ protocols, which are briefly introduced in Table 1. DNA was isolated from each fruit in triplicate, and concentration and purity of every sample was determined by UV/VIS spectrophotometry (NanoDrop 2000, Fisher Scientific, Hampton, NH, USA).

### 4.4. Real-Time PCR

All PCR reactions were performed in RotorGene 6000 (Corbett Life Science, city, Germany). All assays were prepared using SYTO9 MasterMix (Top Bio, Vestec, Czech Republic) containing the intercalating dye SYTO9 (excitation at 485 nm and emission at 498 nM) according to the manufacturer’s instructions. Each DNA isolate was used in one plant specific and one species specific reaction (similar approach regarding the number of real-time PCR replicates was chosen by Paulos et al.) [8]. The specificity of all primer pairs except for the s2 + s3 pair was verified using the Primer Blast Aplication at ncbi.gov [14]. All primers were obtained from Elisabeth Pharmacon. Sequences of all primers are introduced in Table 3 [15,16,17,18,19,20,21,22,23,24]. All PCR reactions were performed in 25 µL of the total volume. Each reaction mixture contained 9.5 μL of PCR water, 12.5 µL of SYTO9 MasterMix, 1 μL of each primer (solutions diluted to 10 pmol/µL), and 1 μL of template DNA of concentrations shown in Table 3. Cycling profiles for each pair of primers are introduced in Table 3, too [15,16,17,18,19,20,21,22,23,24]. The reason for using undiluted DNA isolates as opposed to adjusting the concentration of all DNA isolates to the same value was the fact that the different dilutions would also influence the concentration of contaminants in the DNA isolates, which could in turn influence the results. DNA isolates obtained by a certain method could seem better amplifiable than others, but this result would not be due to the better quality of DNA, but due to the dilution of inhibitory compounds present in the isolates.

### 4.5. Agarose Gel Electrophoresis of PCR Products

The PCR products of the S2 + S3 primer pair were analyzed using 1.2% TBE agarose gel at 30 V for 5 h. The PCR products of DNA amplification of individual plant species using specific primers were analyzed using 1.2% TBE agarose gel at 80 V for 1 h. Amplicons were visualized using 302 nm UV light and Gel Red intercalating dye (Biotium, Fremont, CA, USA) according to the manufacturer’s protocol. The gels were documented by Azure c200 documentation system (Azure Biosystems, Dublin, CA, USA).

## 5. Conclusions

The goal of present study is to compare several protocols for isolation of DNA from different mature fruits to obtain DNA of sufficient quality for PCR-based methods. The purity of DNA isolates was assessed based on the absorbance ratios A260/A280 and A260/A230. The highest DNA purity overall was given by kits 2 (Elisabeth Pharmacon) and 5 (Tools). Amplifiability of isolated DNA was evaluated using two types of real-time PCR assays.

Based on qPCR results, we consider kit 5 (Tools) as the most appropriate for DNA isolation from red fruit. Kit 5 proved to be also the best choice for DNA isolation from the stone fruit and foodstuffs with high content of plum, apricot, or peach matrix. Kit 5 (and kit 1) also belonged to the most suitable for DNA isolation from both mango and banana. Kit 3 can be a suitable alternative for apricot, stone fruit, and red fruit as well. As the most appropriate for DNA isolation from pear and apple, kits 2 and 3, respectively, are proposed.

When considered together, the most universal were kit 2 (Elisabeth Pharmacon), kit 3 (Invitrogen), and kit 5 (Tools). These kits consistently provided amplifiable DNA for PCR-based assays from most of the fruit species used in this study, including more problematic samples.

## Figures and Tables

**Table 1:** Mean values of total quantity of isolated DNA, DNA concentration, and absorbance ratios. c—DNA concentration, A260/A280 and A260/230—absorbance ratios, m—total amount of isolated DNA.

Fruit	Method	c (ng·µL^−1^)	A260/A280	A260/A230	m (ng)
Banana	1	5.0 ± 1.7	1.40 ± 0.09	0.36 ± 0.05	500 ± 167
	2	3.5 ± 1.0	1.95 ± 0.07	1.04± 0.06	350 ± 101
	3	5.4 ± 0.8	2.13 ± 0.15	1.46 ± 0.63	540 ± 78
	4	4.6 ± 1.5	1.23 ± 0.05	0.27 ± 0.16	460 ± 150
	5	4.1 ± 11.6	1.81 ± 0.34	0.27 ± 0.16	410 ± 1164
	6	227.5 ± 37.5	1.59 ± 0.04	1.66 ± 0.16	22,750 ± 3747
Bilberry	1	23.2 ± 21.3	1.18 ± 0.17	0.43 ± 0.10	2320 ± 2134
	2	1.7 ± 0.2	1.65 ± 0.47	0.28 ± 0.35	170 ± 21
	3	48.5 ± 14.7	0.63 ± 0.26	0.14 ± 0.06	4850 ± 1468
	4	3.0 ± 1.0	0.99 ± 0.07	0.30 ± 0.03	300 ± 103
	5	4.3 ± 6.7	1.96 ± 0.25	0.33 ± 0.82	430 ± 668
	6	80.5 ± 26.4	1.39 ± 0.03	1.66 ± 0.22	8050 ± 2644
Pear	1	41.3 ± 23.5	1.39 ± 0.14	0.57 ± 0.19	4130 ± 2,353
	2	5.6 ± 1.2	2.60 ± 0.14	0.55 ± 0.07	560 ± 121
	3	5.7 ± 0.4	1.54 ± 0.07	0.59 ± 0.04	570 ± 36
	4	4.1 ± 0.5	1.10 ± 0.13	0.28 ± 0.03	410 ± 50
	5	5.6 ± 3.0	1.73 ± 0.31	0.32 ± 0.77	560 ± 303
	6	54.9 ± 11.0	1.36 ± 0.01	1.61 ± 0.15	5490 ± 1104
Apple	1	11.8 ± 3.1	1.21 ± 0.04	0.37 ± 0.06	1180 ± 305
	2	1.6 ± 1.3	2.37 ± 2.32	0.22 ± 0.06	160 ± 126
	3	3.9 ± 0.7	1.43 ± 0.03	0.51 ± 0.04	390 ± 66
	4	15.7 ± 1.7	1.19 ± 0.02	0.21 ± 0.03	1570 ± 168
	5	3.3 ± 1.9	1.59 ± 0.24	0.53 ± 2.97	330 ± 193
	6	67.5 ± 7.4	1.39 ± 0.02	1.48 ± 0.08	6750 ± 745
Strawberry	1	7.0 ± 0.4	1.25 ± 0.10	0.39 ± 0.04	700 ± 35
	2	6.0 ± 2.9	2.08 ± 0.13	1.11 ± 0.21	600 ± 295
	3	19.0 ± 24.9	0.60 ± 0.12	0.14 ± 0.04	1900 ± 2491
	4	3.1 ± 7.8	1.17 ± 0.05	0.41 ± 0.05	310 ± 783
	5	2.6 ± 0.6	1.53 ± 0.69	1.98 ± 5,18	260 ± 56
	6	298.5 ± 159.1	1.59 ± 0.13	1.82 ± 0.05	29,850 ± 15,914
Raspberry	1	241.9 ± 44.2	1.50 ± 0.05	0.37 ± 0.03	24,190 ± 4423
	2	4.0 ± 2.1	2.09 ± 0.08	1.02 ± 0.20	400 ± 211
	3	6.3 ± 1.3	1.39 ± 0.10	1.17 ± 0.10	630 ± 125
	4	1.7 ± 0.9	1.25 ± 0.27	0.33 ± 0.07	170 ± 86
	5	9.3 ± 19.4	1.99 ± 0.04	0.38 ± 2.15	930 ± 1938
	6	650.3 ± 65.2	1.43 ± 0.02	1.78 ± 0.18	65,030 ± 6524
Apricot	1	48.2 ± 16.4	1.24 ± 0.06	0.43 ± 0.04	4820 ± 1638
	2	0.9 ± 0.4	1.20 ± 0.35	0.19 ± 0.14	90 ± 40
	3	33.3 ± 14.6	0.76 ± 0.19	0.16 ± 0.04	3330 ± 1456
	4	2.2 ± 0.3	1.11 ± 0.09	0.23 ± 0.03	220 ± 30
	5	4.6 ± 2.0	1.93 ± 0.16	0.27 ± 0.06	460 ± 203
	6	75.9 ± 23.1	1.43 ± 0.03	1.53 ± 0.06	7590 ± 2308
Plum	1	50.4 ± 21.9	1.25 ± 0.06	0.34 ± 0.04	5040 ± 2187
	2	1.7 ± 0.3	2.29 ± 0.20	0.41 ± 0.07	170 ± 30
	3	18.9 ± 3.2	1.18 ± 0.05	0.39 ± 0.04	1890 ± 323
	4	2.1 ± 0.2	1.05 ± 0.13	0.27 ± 0.12	210 ± 15
	5	4.1 ± 0.4	1.56 ± 0.44	0.49 ± 0.11	410 ± 42
	6	101.2 ± 12.0	1.38 ± 0.02	1.14 ± 0.06	10,120 ± 1196
Peach	1	4.3 ± 2.1	1.30 ± 0.48	0.23 ± 0.05	430 ± 206
	2	15.4 ± 0.8	2.25 ± 0.05	1.82 ± 0.21	1540 ± 75
	3	2.7 ± 1.1	1.28 ± 1.43	0.32 ± 0.41	270 ± 108
	4	14.3 ± 3.9	1.25 ± 0.05	0.26 ± 0.05	1430 ± 391
	5	3.1 ± 0.2	1.80 ± 0.15	1.67 ± 1.61	310 ± 21
	6	184.2 ± 30.5	1.65 ± 0.01	1.45 ± 0.08	18,420 ± 3054
Mango	1	2.6 ± 7.0	1.75 ± 0.58	0.27 ± 0.11	260 ± 696
	2	8.5 ± 1.7	2.68 ± 0.10	1.43 ± 0.44	850 ± 170
	3	1.9 ± 0.7	1.55 ± 3,8	1.69 ± 3,41	190 ± 66
	4	4.1 ± 0.5	1.54 ± 0.02	0.59 ± 0.01	410 ± 47
	5	4.7 ± 2.2	1.81 ± 0.22	0.66 ± 32,58	470 ± 217
	6	123.8 ± 12.5	1.57 ± 0.02	1.63 ± 0.08	12,380 ± 1246

**Table 2:** Ct values of real-time PCR reactions with strawberry, raspberry, and bilberry DNA isolates. Each Ct value represents a different DNA preparation. The Column “Figure” refers to corresponding figures in supplementary files.

Fruit	Method	C_t_—Plant Specific Primers			C_t_—Species Specific Primers			Figure
Strawberry	Kit 1 (Qiagen)	21.64	21.24		26.91	25.03	24.77	S9, 1,2 13, 14
	Kit 2 (Elisabeth Pharmacon)	19.52	20.21	33.54	23.56	25.13	29.28	S9, 3, 4, 15, 16
	Kit 3 (Invitrogen)	18.18	18.48	19.07	21.11	22.22	22.88	S9, 5, 6, 17, 18
	Kit 4 (PerkinElmer)	22.48	21.98	21.56	26.46	32.49	26.34	S9, 7, 8, 19, 20
	Kit 5 (Tools)	18.19	18.19	17.25	20.16	24.03	23.01	S9, 9, 10, 21, 22
	6 (CTAB protocol)	32.15	24.0	21.17		29.81	31.47	S9, 11, 12, 23, 24
Raspberry	Kit 1 (Qiagen)		33.25	31.18				S10, 1, 2, 13, 14
	Kit 2 (Elisabeth Pharmacon)	16.62	22.15	18.33	28.14	23.79	22.66	S10, 3, 4, 15, 16
	Kit 3 (Invitrogen)	18.05	17.31	19.28	25.08	24.91	25.47	S10, 5, 6, 17, 18
	Kit 4 (PerkinElmer)	19.21	19.13	17.33	27.05	25.68	24.9	S10, 7, 8, 19, 20
	Kit 5 (Tools)	15.01	16.57	18.32	21.98	24.11	25.3	S10, 9, 10, 21, 22
	6 (CTAB protocol)	20.6	17.62	24.1	25.25	23.88	29.58	S10, 11, 12, 23, 24
Bilberry	Kit 1 (Qiagen)	19.71	18.83	19.1	26.72	24.77	24.09	S5, 1, 2, 13, 14
	Kit 2 (Elisabeth Pharmacon)	19.94	20.55	14.38	28.87	31.31	28.16	S5, 3, 4, 15, 16
	Kit 3 (Invitrogen)	17.45	16.93	17.73	22.84	22.61	23.49	S5, 5, 6, 17, 18
	Kit 4 (PerkinElmer)	22.6	23.91	21.07	28.27	28.78	26.42	S5, 7, 8, 19, 20
	Kit 5 (Tools)	16.75	18.08	16.09	22.36	24.37	23.56	S5, 9, 10, 21, 22
	6 (CTAB protocol)	31.01	29.32	27.87			30.36	S5, 11, 12, 23, 24

**Table 3:** Ct values of real-time PCR reactions with plum, apricot, and peach DNA isolates. Each Ct value represents a different DNA preparation. The Column “Figure” refers to corresponding figures in supplementary files.

Fruit	Method	C_t_—Plant Specific Primers			C_t_—Species Specific Primers			Figure
Plum	Kit 1 (Qiagen)	30.96	33.41	30.06		29.56	29.62	S13, 1, 2, 13, 14
	Kit 2 (Elisabeth Pharmacon)	22.91	21.94	21.74	20.78	22.71	22.56	S13, 3, 4. 15, 16
	Kit 3 (Invitrogen)	31.12	30.39	35.6	21.91	30.5	29.98	S13, 5, 6, 17, 18
	Kit 4 (Perkin-Elmer)	23.75	24.05	21.44	25.82	25.79	24.32	S13, 7, 8, 19, 20
	Kit 5 (Tools)	18.61	20.06	19.78	21.37	23.53	22.26	S13, 9, 10, 21, 22
	6 (CTAB protocol)	29.36	31.69	28.25	31.24	25.34	28.73	S13, 23, 24
Peach	Kit 1 (Qiagen)	16.09	18.53	17.48	23.96	28.22	27.43	S6, 1, 2, 13, 14
	Kit 2 (Elisabeth Pharmacon)	19.43	20.49	19.54	28.46	26.64	27.27	S6, 3, 4, 15, 16
	Kit 3 (Invitrogen)	18.85	17.98	18.45	26.59	26.36	26.13	S6, 5, 6, 17, 18
	Kit 4 (Perkin-Elmer)	20.08	19.66	21.11	27.93	27.09	26.36	S6, 7, 8, 19, 20
	Kit 5 (Tools)	15.68	18.09	17.06	25.25	26.43	25.51	S6, 9, 10, 21, 22
	6 (CTAB protocol)	20.61	20.5	20.27	26.06	24.61	27.04	S6, 11, 12, 23, 24
Apricot	Kit 1 (Qiagen)	31.38		32.25		31.85		S12, 1, 2, 13, 14
	Kit 2 (Elisabeth Pharmacon)	25.07	23.51	30.76		30.42	30.96	S12, 3, 4, 15, 16
	Kit 3 (Invitrogen)	17.94	15.61	17.26	25.78	24.27	26.16	S12, 5, 6, 17, 18
	Kit 4 (Perkin-Elmer)	24.65	23.54	24.48	32.28		29.5	S6, 7, 8, 19, 20
	Kit 5 (Tools)	18.51	19.05	18.28	27.17	27.93	28.99	S6, 9, 10, 21, 22
	6 (CTAB protocol)	29.76	28.82	24.92			32.0	S6, 11, 12, 23, 24

**Table 4:** Ct values of real-time PCR reactions with banana and mango DNA isolates. Each Ct value represents a different DNA preparation. The Column “Figure” refers to corresponding figures in supplementary files.

Fruit	Method	C_t_—Plant Specific Primers			C_t_—Species Specific Primers			Figure
Banana	Kit 1 (Qiagen)	16.51	16.97	18.13	21.04	21.63	23.06	S4, 1, 2, 13, 14
	Kit 2 (Elisabeth Pharmacon)	17.38	19.24	19.06	24.2	22.31	23.45	S4, 3, 4, 15, 16
	Kit 3 (Invitrogen)	20.09	20.38	19.61	23.03	24.2	23.85	S4, 5, 6, 17, 18
	Kit 4 (Perkin-Elmer)	22.25	23.52	23.76	25.69	25.3	24.42	S4, 7, 8, 19. 20
	Kit 5 (Tools)	18.77	17.4	18.56	20.77	17.87	20.75	S4, 9, 10, 21, 22
	6 (CTAB protocol)	20.82	29.96	28.24	25.24	32.1	29.47	S4, 11, 12, 23, 24
Mango	Kit 1 (Qiagen)	22.43	19.25	18.52	21.98	20.72	20.86	S11, 1, 2, 13, 14
	Kit 2 (Elisabeth Pharmacon)	23.26	23.75	22.83		30.92	30.0	S11, 3, 4, 15, 16
	Kit 3 (Invitrogen)	21.83	21.92	22.05	25.03	25.77	25.94	S11, 5, 6, 17, 18
	Kit 4 (Perkin-Elmer)	21.54	22.39	21.7	25.11	26.2	25.17	S11, 7, 8, 19, 20
	Kit 5 (Tools)	16.91	18.02	17.33	21.18	24.01	21.98	S11, 9, 10, 21, 22
	6 (CTAB protocol)	21.15	21.78	25.03	25.62	26.64	28.02	S11, 11, 12, 23, 24

**Table 5:** Ct values of real-time PCR reactions with pear and apple DNA isolates. Each Ct value represents a different DNA preparation. The Column “Figure” refers to corresponding figures in supplementary files.

Fruit	Method	C_t_—Plant Specific Primers			C_t_—Species Specific Primers			Figure
Pear	Kit 1 (Qiagen)	25.23	24.17	26.28	29.03	28.36	28.91	S7, 1, 2, 13, 14
	Kit 2 (Elisabeth Pharmacon)	21.89	20.94	19.35	26.08	24.97	23.88	S7, 3, 4, 15, 16
	Kit 3 (Invitrogen)	26.04	26.27	26.29	29.06	29.71	29.11	S7, 5, 6, 17, 18
	Kit 4 (Perkin-Elmer)	25.27	27.43	26.34	26.77	28.73	26.54	S7, 7, 8, 19, 20
	Kit 5 (Tools)	25.92	24.63	24.35	27.84	28.48	29.93	S7, 9, 10, 21, 22
	6 (CTAB protocol)	29.58	31.65	32.42	28.27	26.65	27.97	S7, 11, 12, 23, 24
Apple	Kit 1 (Qiagen)	28.24	27.25	27.89	32.27	35.9	32.84	S8, 1, 2, 13, 14
	Kit 2 (Elisabeth Pharmacon)	29.34	27.93	29.11		34.72	32.68	S8, 3, 4, 15, 16
	Kit 3 (Invitrogen)	27.38	27.52	25.88	30.76	30.87	30.79	S8, 5, 6, 17, 18
	Kit 4 (Perkin-Elmer)	27.4	28.08	27.56		32.75		S8, 7, 8, 19, 20
	Kit 5 (Tools)	28.05	27.84	27.69	29.48	30.62		S8, 9, 10, 21, 22
	6 (CTAB protocol)	32.99	29.13	31.26	30.96		32.32	S8, 11, 12, 23, 24

**Table 6:** DNA isolation methods used in this work.

Method No.	Manufacturer	Kit/Method	Sample Homogenization	DNA Capture and Purification
1	Qiagen	DNEasy PowerPlant Pro Kit	Bead-beating of small pieces of plant tissue at room temperature	Spin column
2	Elisabeth Pharmacon	EliGene Plant DNA Isolation Kit	Mechanical (pestle and sand) and chemical (detergent), at room temperature	Capture on a spin filter in the presence of a chaotropic detergent
3	Invitrogen	ChargeSwitch gDNA Plant Kit	Grinding with mortar and pestle in liquid nitrogen	Magnetic beads positively charged in acidic pH and neutral in pH above 8.5
4	Perkin- Elmer	Chemagic DNA Plant Kit	Grinding with mortar and pestle in liquid nitrogen	Magnetic beads with chaotropic salt
5	Tools	Easy Prep Polyphenol Plant DNA Extraction Kit	Grinding with mortar and pestle in liquid nitrogen	Spin column
6	-	CTAB protocol	Grinding with mortar and pestle in liquid nitrogen	Isopropanol and ethanol precipitation [12,13]

## Supplementary Materials

The following are available online. File S1: Primer sequences, File S2: Electrophoresis, ITS2, File S3: Electrophoresis, species specific assays, File S4,: Banana qPCR, File S5: Bilberry qPCR, File S6: Peach qPCR, File S7: Pear qPCR, File S8: Apple qPCR, File S9: Strawberry qPCR, File S10: Raspberry qPCR, File S11: Mango qPCR, File S12: Apricot qPCR, File S13: Plum qPCR, File S14: DNA amounts in qPCR assays, File S15: Workflow chart.
