# Supplementary material for: A Comparative Study of Some Procedures for Isolation of Fruit DNA of Sufficient Quality for PCR-Based Assays
Source: Molecules. 2020 Sep 20;25(18):4317. doi: 10.3390/molecules25184317 (PMC7570663; doi:10.3390/molecules25184317)
Supplement: Supplementary file 1 [file molecules-25-04317-s001.zip › molecules-913289-supplementary-revised-2nd - original/molecules 913289/S1 Primer sequences.pdf]

Primer pair	Specificity	Forward primer	Reverse primer	Amplicon size	Profile		Ref.
S2F +S3R	Plant ITS2 region	ATGCGATACTTGGTGTGAAT	GACGCTTCTCCAGACTACAAT	400-533 bp	95°C for 30 seconds		9
					95°C for 30 seconds<br>56°C for 30 seconds<br>72°C for 30 seconds	40x	
					72°C for 10 minutes		
Prupe 4053	Peach	ACCCACAACAAGAACAACAGTCC	CCCTCGTGTAAAAATTCATCCAC	171 bp	95°C for 30 seconds		10
					95°C for 5 seconds<br>55°C for 30 seconds<br>72°C for 30 seconds	40x	
					72°C for 5 minutes		
APX1B	Strawberry	CTGGAGTTGTTGCTGTTGAGG	CTTTCCAGCATCAGGAAGACG	680 bp	94°C for 2 minutes		11
					94°C for 30 seconds	40x	
					60°C for 45 seconds with touchdown to 55°C in 10 cycles with 0.5°C step		
					72°C for 60 seconds		
					72°C for 5 minutes		
AGS6	Apricot	GAGTGGCCGATACCTGTTCT	AATGATGGGTTTTGGGTGTG	222 bp	94°C for 2 minutes		11
					94°C for 30 seconds		
					60°C for 45 seconds with touchdown to 55°C in 10 cycles with 0.5°C step	40x	
					72°C for 60 seconds		
					72°C for 5 minutes		
Mito-Foc-S-001	Banana	CTCGCCGACACCTTACTTGAT	GGGGTCTCGTTGCTTGTCTC	277 bp	94°C for 5 minutes		12
					94°C for 30 seconds	35x	
					58°C for 30 seconds		
					72°C for 30 seconds		
					72°C for 7 minutes		

FT MDP	Apple	GCCAGCGAGGTTTCAACTTCTT	TGCCGCAGTAGTTGCTGGAATA	128 bp	94°C for 2 minutes		13
					94°C for 30 seconds	40x	
					55°C for 30 seconds		
					72°C for 30 seconds		
					72°C for 2 minutes		
RiACO1	Raspberry	AATTGTTTGGAGCAGAGATTCAAGG	AAACTCCTTCATCACCTTCCTGTAG	177 bp	95°C for 10 minutes		14
					95°C for 2 minutes	35x	
					60°C for 15 seconds		
					72°C for 25 seconds		
					72°C for 5 minutes		
VcBHLH003	Blueberry	AAATGGATTTGCTGTTATGGGTG	GGAATCATTAGGGAAACTGGGTA	226 bp	95°C for 5 minutes		15
					95°C for 30 seconds	30x	
					55°C for 30 seconds		
					72°C for 30 seconds		
					72°C for 5 minutes		
PAL	Mango	TGGATTCAAGGGTGCTGAAATCGC	TCACATCTTGGTTGTGTTGCTCGG	113 bp	95°C for 5 minutes		16
					95°C for 10 seconds	40x	
					60°C for 10 seconds		
					72°C for 30 seconds		
					72°C for 5 minutes		
PdCass	Plum	CCGTGAGGGCAGAGAGGGGG	GCTCCCAAAAGGCCTCGTGCT	236 bp	95°C for 5 minutes		17
					95°C for 10 seconds	35x	
					58°C for 10 seconds		
					72°C for 30 seconds		
					72°C for 5 minutes		
Pear	Pear	GACCTGCCAATGTTAATGC	CAGCAGTACTTCGAATCACC	115 bp	95°C for 5 minutes		18
					95°C for 15 seconds	40x	
					55°C for 30 seconds		
					72°C for 30 seconds		
					72°C for 5 minutes		
