# Supplementary material for: A Comparative Study of Some Procedures for Isolation of Fruit DNA of Sufficient Quality for PCR-Based Assays
Source: Molecules. 2020 Sep 20;25(18):4317. doi: 10.3390/molecules25184317 (PMC7570663; doi:10.3390/molecules25184317)
Supplement: Supplementary file 1 [file molecules-25-04317-s001.zip › molecules-913289-supplementary-revised-2nd - original/molecules 913289/S14 DNA amounts in qPCR assays.pdf]

Table 1. *Amounts of red fruit DNA amplified in real-time PCR assays*

Fruit	Method	m_1 (ng)	m_2 (ng)	m_3 (ng)
Strawberry	1	7,4	6,7	7
	2	6,0	10,4	4,8
	3	60,9	16,6	19
	4	3,1	15,9	1,7
	5	2,6	2,2	3,3
	6	53,8	298,5	352,4
Raspberry	1	223,9	307,9	241,9
	2	6,9	2,8	4,0
	3	6,3	6	8,3
	4	1,1	1,7	2,8
	5	41,5	6,7	9,3
	6	756,0	636,9	650,3
Bilberry	1	11,5	52,9	23,2
	2	1,8	1,4	1,7
	3	48,5	41,5	69,7
	4	3,0	1,6	3,6
	5	3,6	4,3	15,5
	6	87,5	80,5	38,6

Table 2. *Amounts of stone fruit DNA amplified in real-time PCR assays*

Fruit	Method	m_1 (ng)	m_2 (ng)	m_3 (ng)
Plum	1	50,4	76,1	32,6
	2	1,4	2,0	1,7
	3	14,2	18,9	20,4
	4	2,3	2,1	2,0
	5	4,1	3,9	4,7
	6	120,5	101,2	98,6
Peach	1	6,7	2,6	4,3
	2	16,7	15,4	15,4
	3	1,2	2,7	3,3
	4	14,3	15,5	8,2
	5	3,4	3,1	3
	6	167,2	184,2	226,5
Apricot	1	48,2	54,5	23,5
	2	0,9	0,9	0,2
	3	54,5	26,6	33,3
	4	2,2	2,5	1,9
	5	6	2	4,6
	6	75,9	107,9	63,1

Table 3. *Amounts of tropical fruit DNA amplified in real-time PCR assays*

Fruit	Method	m_1 (ng)	m_2 (ng)	m_3 (ng)
Banana	1	5,0	4,2	7,4
	2	4,7	2,7	3,5
	3	5,4	4,3	5,8
	4	3,0	4,6	6,0
	5	4,1	22,9	1,6
	6	227,5	165,7	233,3
Mango	1	13,6	0,7	2,6
	2	9,1	8,5	5,9
	3	1,1	1,9	2,4
	4	4,8	3,9	4,1
	5	4,6	4,7	8,4
	6	135,3	123,8	110,4

Table 4. *Amounts of pome fruit DNA amplified in real-time PCR assays*

Fruit	Method	m_1 (ng)	m_2 (ng)	m_3 (ng)
Pear	1	45,3	41,3	2,7
	2	5,6	4,2	6,6
	3	5,9	5,7	5,2
	4	4,5	3,5	4,1
	5	5,6	5,5	10,8
	6	64,2	42,2	54,9
Apple	1	11,1	16,7	11,8
	2	0,6	1,6	3,1
	3	4,7	3,4	3,9
	4	15,7	17,9	14,6
	5	2,4	3,3	6,1
	6	67,5	79,7	66,2
