# Supplementary material for: A Comparative Study of Some Procedures for Isolation of Fruit DNA of Sufficient Quality for PCR-Based Assays
Source: Molecules. 2020 Sep 20;25(18):4317. doi: 10.3390/molecules25184317 (PMC7570663; doi:10.3390/molecules25184317)
Supplement: Supplementary file 1 [file molecules-25-04317-s001.zip › molecules-913289-supplementary-revised-2nd - original/molecules 913289/S2 Electrophoresis, ITS2.pdf]

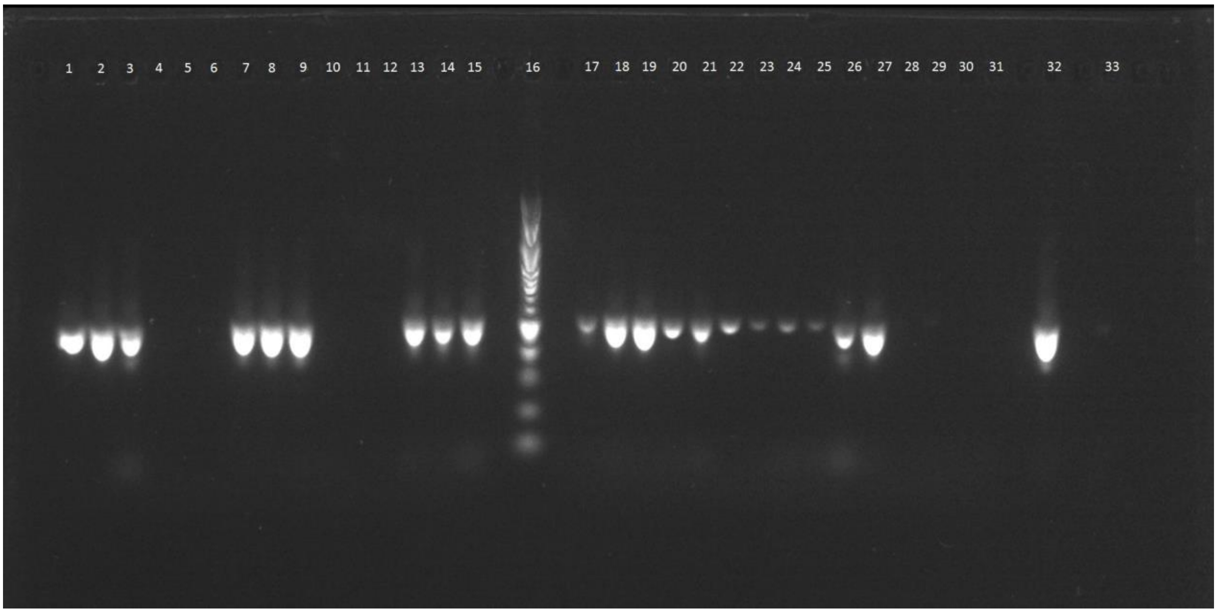

Figure 1: Result of PCR assay with DNA isolates obtained by kit 1. 1-3 banana, 4-6 raspberry, 7-9 peach, 10-12 apricot, 13-15 blueberry, 16-DNA ladder, 17-19 mango, 20-22 pear, 23-25 apple, 26-28 strawberry, 29-31 plum, 32-positive control, 33-no template control

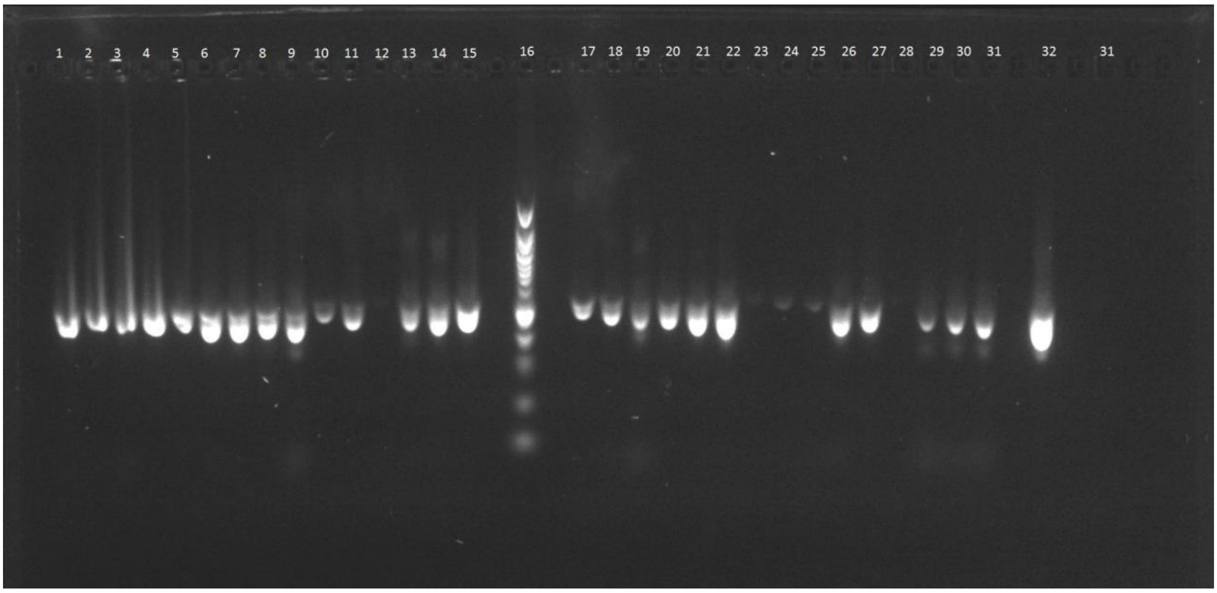

Figure 2: Result of PCR assay with DNA isolates obtained by kit 2. 1-3 banana, 4-6 raspberry, 7-9 peach, 10-12 apricot, 13-15 blueberry, 16-DNA ladder, 17-19 mango, 20-22 pear, 23-25 apple, 26-28 strawberry, 29-31 plum, 32-positive control, 33-no template control

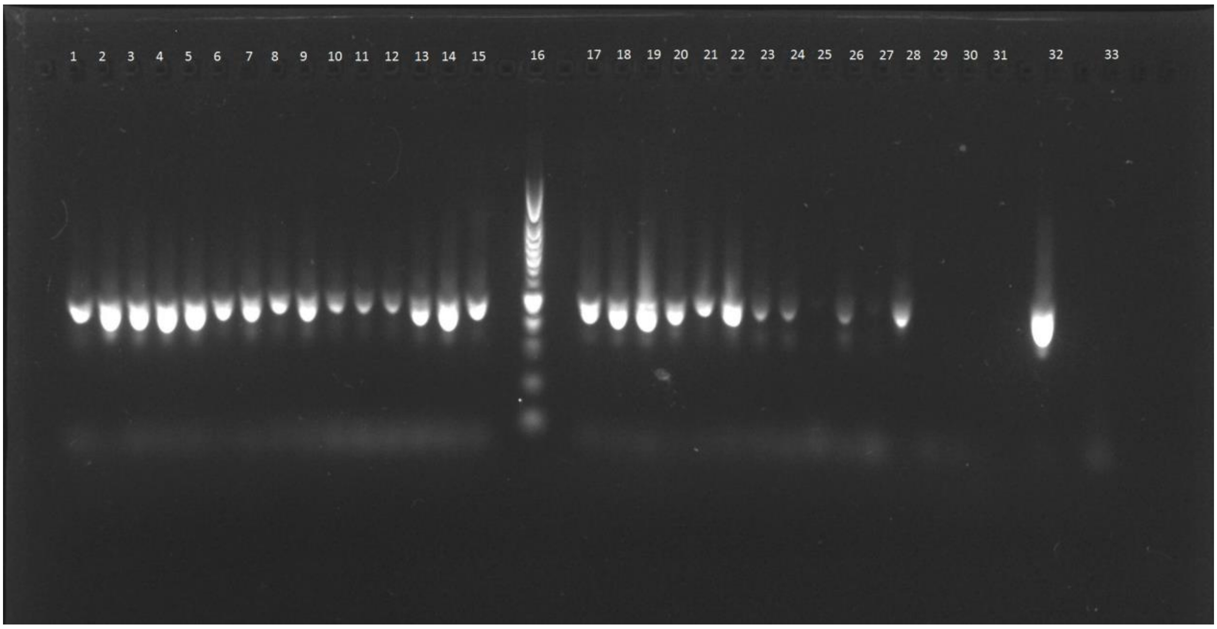

Figure 3: Result of PCR assay with DNA isolates obtained by kit 3: 1-3 banana, 4-6 raspberry, 7-9 blueberry, 10-12 mango, 13-15 peach, 16-DNA ladder, 17-19 apricot, 20-22 strawberry, 23-25 pear, 26-28 apple, 29-31 plum, 32 positive control, 33 no template control

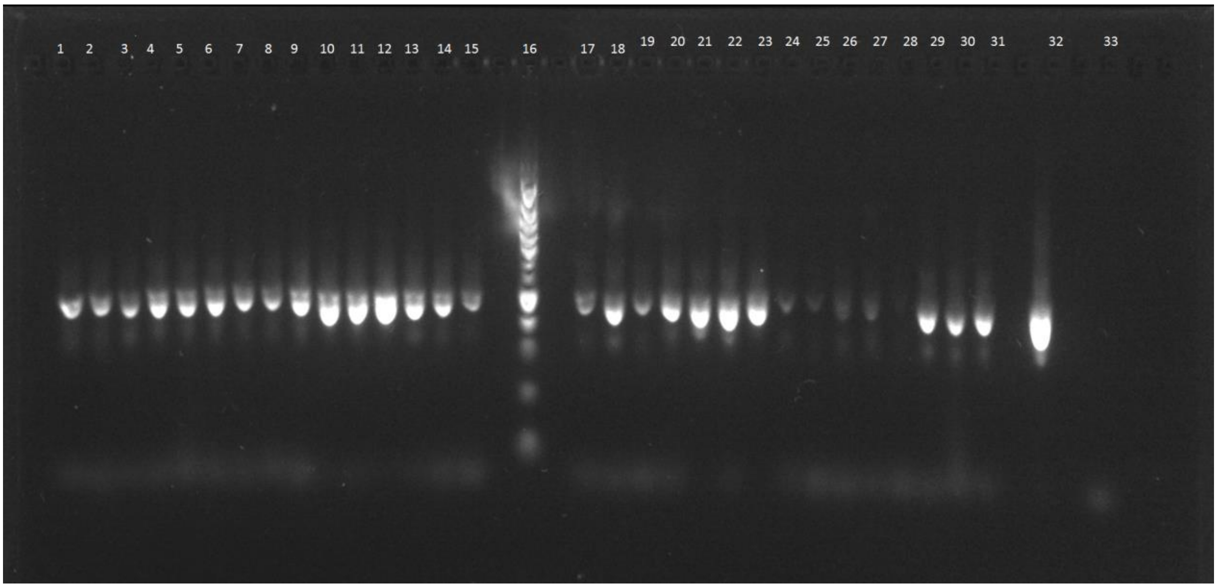

Figure 4: Result of PCR assay with DNA isolates obtained by kit 4: 1-3 banana, 4-6 raspberry, 7-9 blueberry, 10-12 mango, 13-15 peach, 16-DNA ladder, 17-19 apricot, 20-22 strawberry, 23-25 pear, 26-28 apple, 29-31 plum, 32 positive control, 33 no template control

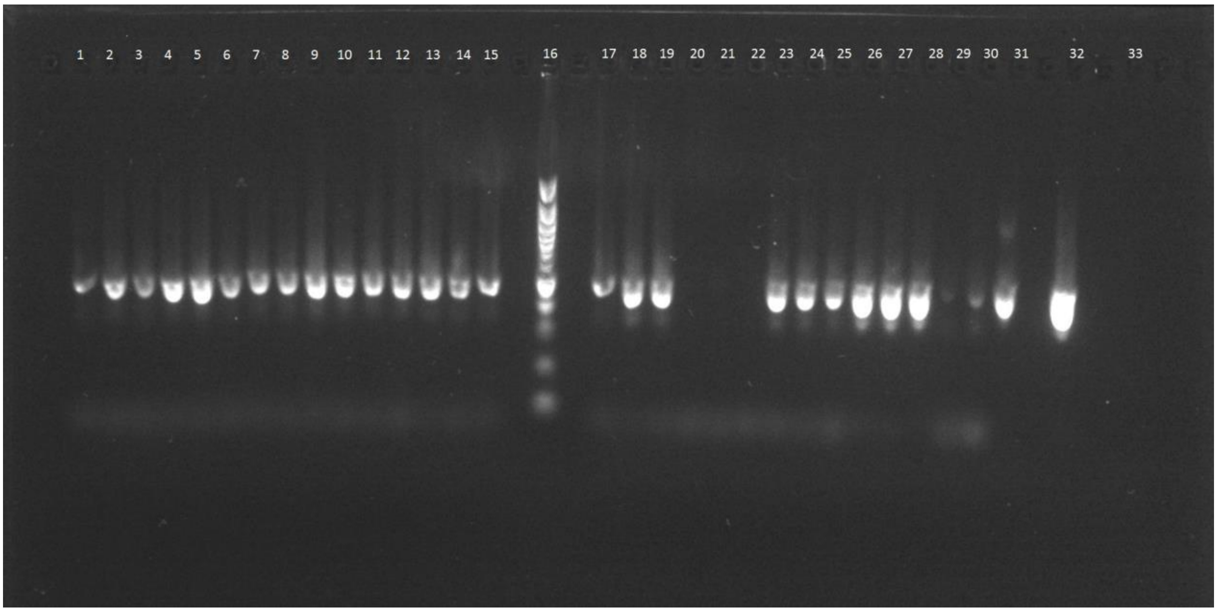

Figure 5: Result of PCR assay with DNA isolates obtained by kit 5: 1-3 banana, 4-6 raspberry, 7-9 blueberry, 10-12 mango, 13-15 peach, 16-DNA ladder, 17-19 apricot, 20-22 apple, 23-25 plum, 26-28 strawberry, 29-31 pear, 32 positive control, 33 no template control

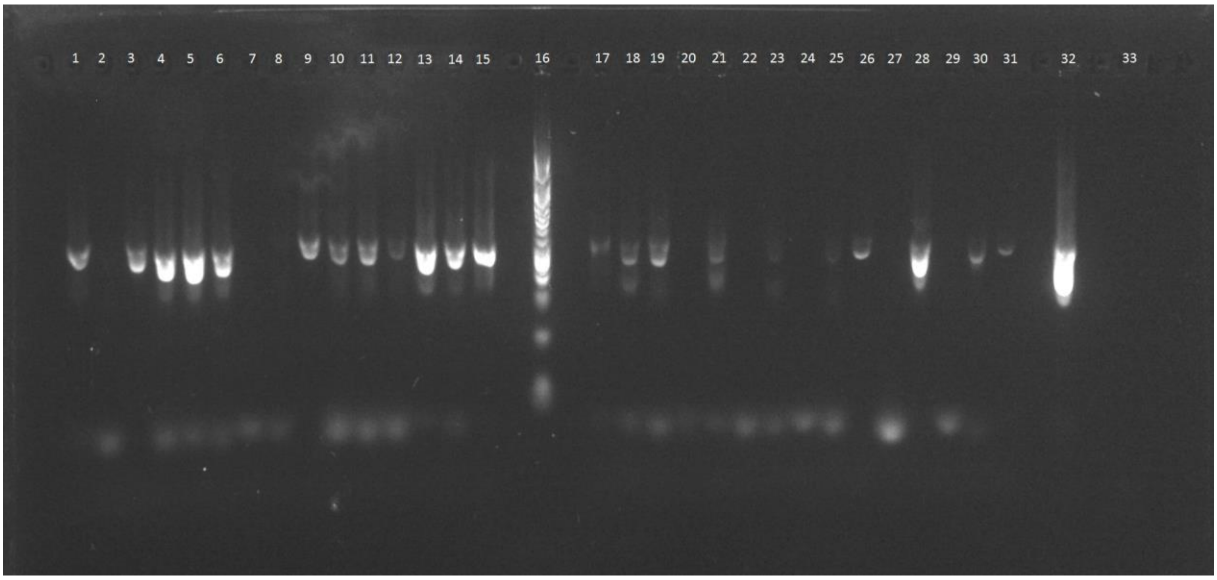

Figure 6: Result of PCR assay with DNA isolates obtained by CTAB method: 1-3 banana, 4-6 raspberry, 7-9 blueberry, 10-12 mango, 13-15 peach, 16-DNA ladder, 17-19 apricot, 20-22 strawberry, 23-25 pear, 26-28 apple, 29-31 plum, 32 positive control, 33 no template control
