# Supplementary material for: A Comparative Study of Some Procedures for Isolation of Fruit DNA of Sufficient Quality for PCR-Based Assays
Source: Molecules. 2020 Sep 20;25(18):4317. doi: 10.3390/molecules25184317 (PMC7570663; doi:10.3390/molecules25184317)
Supplement: Supplementary file 1 [file molecules-25-04317-s001.zip › molecules-913289-supplementary-revised-2nd - original/molecules 913289/S3 Electrophoresis, species specific assays.pdf]

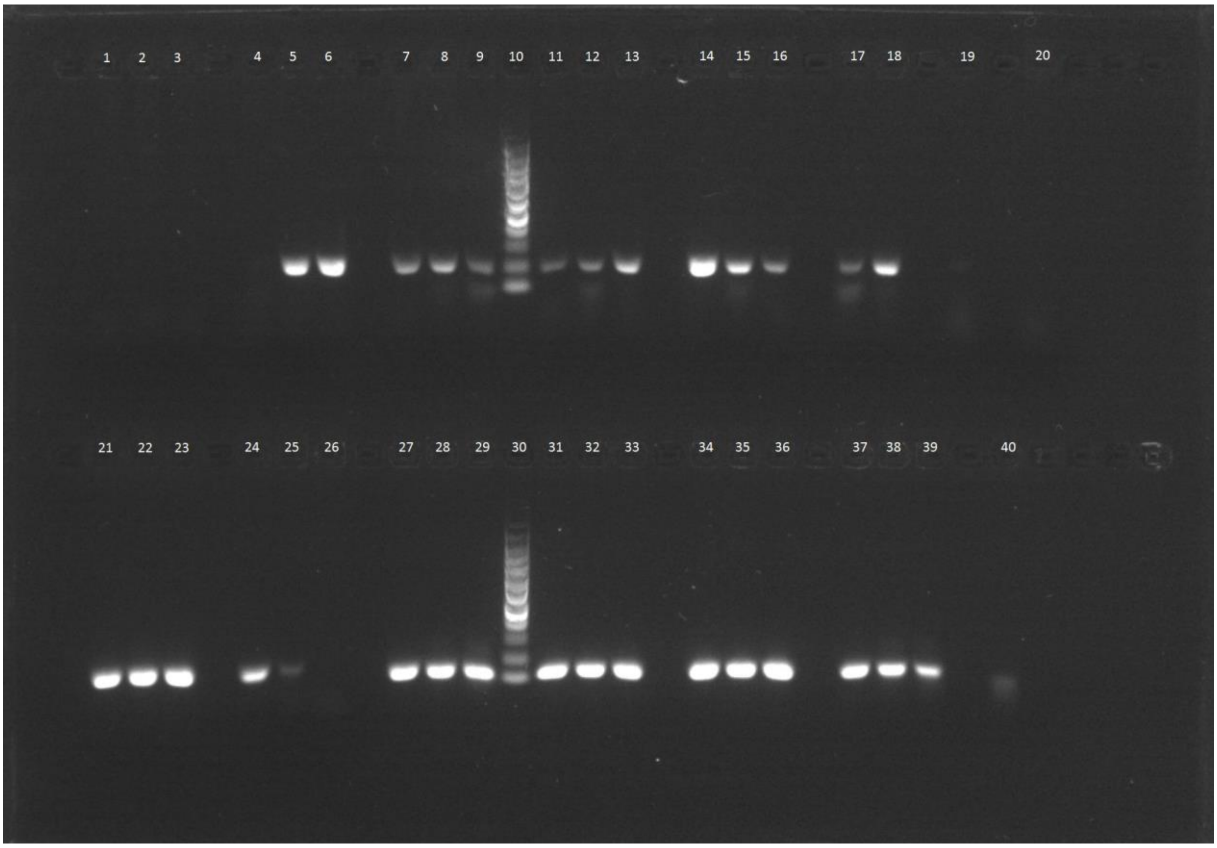

Figure1: Result of PCR assay with species specific primers. Upper row: Raspberry, 1-3 kit 1, 4-6 kit 2, 7-9 kit 3, 10 DNA ladder, 11-13 kit 4, 14-16 kit 5, 17-19 CTAB protocol, 20 no template control. Lower row: Mango, 21-23 kit 1, 24-26 kit 2, 27-29 kit 3, 30 DNA ladder, 31-33 kit 4, 34-36 kit 5, 37-39 CTAB protocol, 40 no template control.

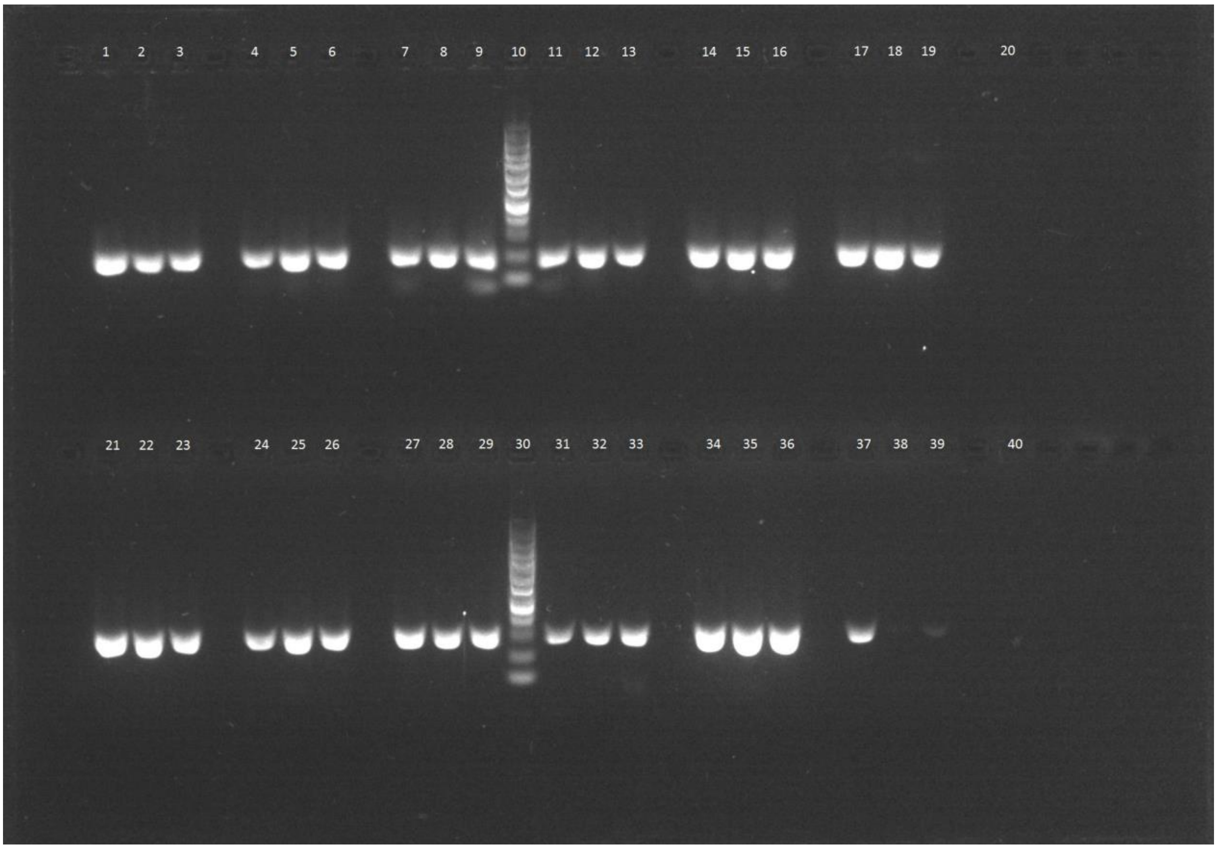

Figure 2: Peach – result of PCR assay with species specific primers: 1-3 kit 1, 4-6 kit 2, 7-9 kit 3, 10 DNA ladder, 11-13 kit 4, 14-16 kit 5, 17-19 CTAB protocol, 20 no template control. Banana: 21-23 kit 1, 24-26 kit 2, 27-29 kit 3, 30 DNA ladder, 31-33 kit 4, 34-36 kit 5, 37-39 CTAB protocol, 40 no template control.

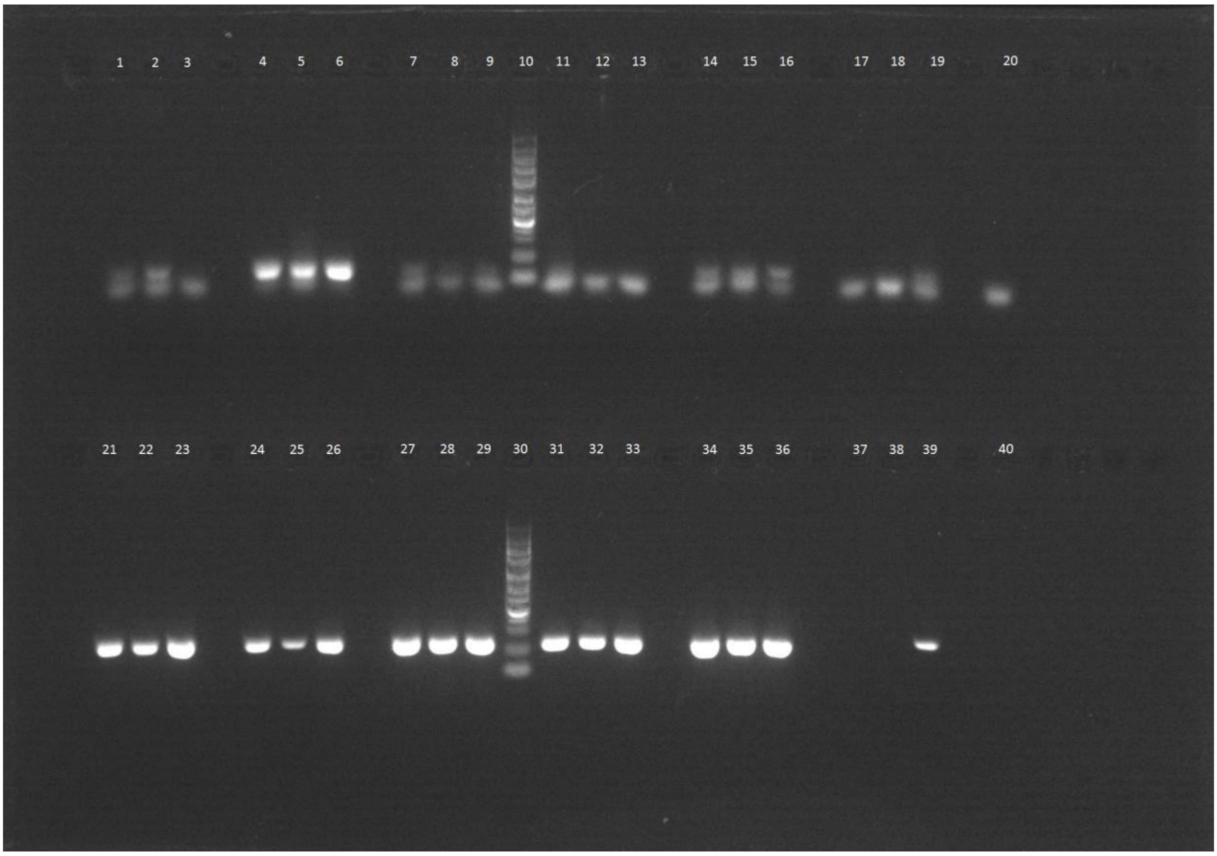

Figure 3: Pear – result of PCR assay with species specific primers: 1-3 kit 1, 4-6 kit 2, 7-9 kit 3, 10 DNA ladder, 11-13 kit 4, 14-16 kit 5, 17-19 CTAB protocol, 20 no template control. Blueberry: 21-23 kit 1, 24-26 kit 2, 27-29 kit 3, 30 DNA ladder, 31-33 kit 4, 34-36 kit 5, 37-39 CTAB protocol, 40 no template control.

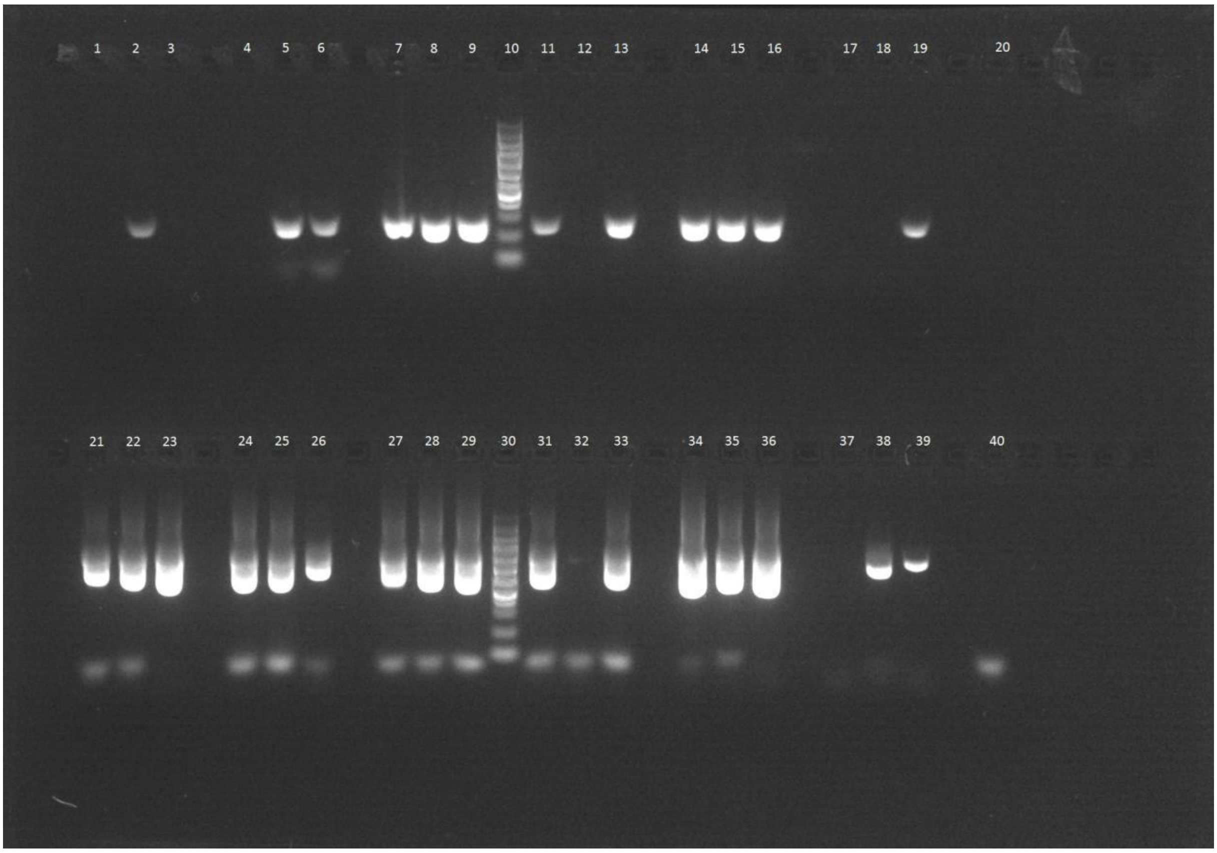

Figure 4: Apricot – result of PCR assay with species specific primers: 1-3 kit 1, 4-6 kit 2, 7-9 kit 3, 10 DNA ladder, 11-13 kit 4, 14-16 kit 5, 17-19 CTAB protocol, 20 no template control. Strawberry: 21-23 kit 1, 24-26 kit 2, 27-29 kit 3, 30 DNA ladder, 31-33 kit 4, 34-36 kit 5, 37-39 CTAB protocol, 40 no template control.

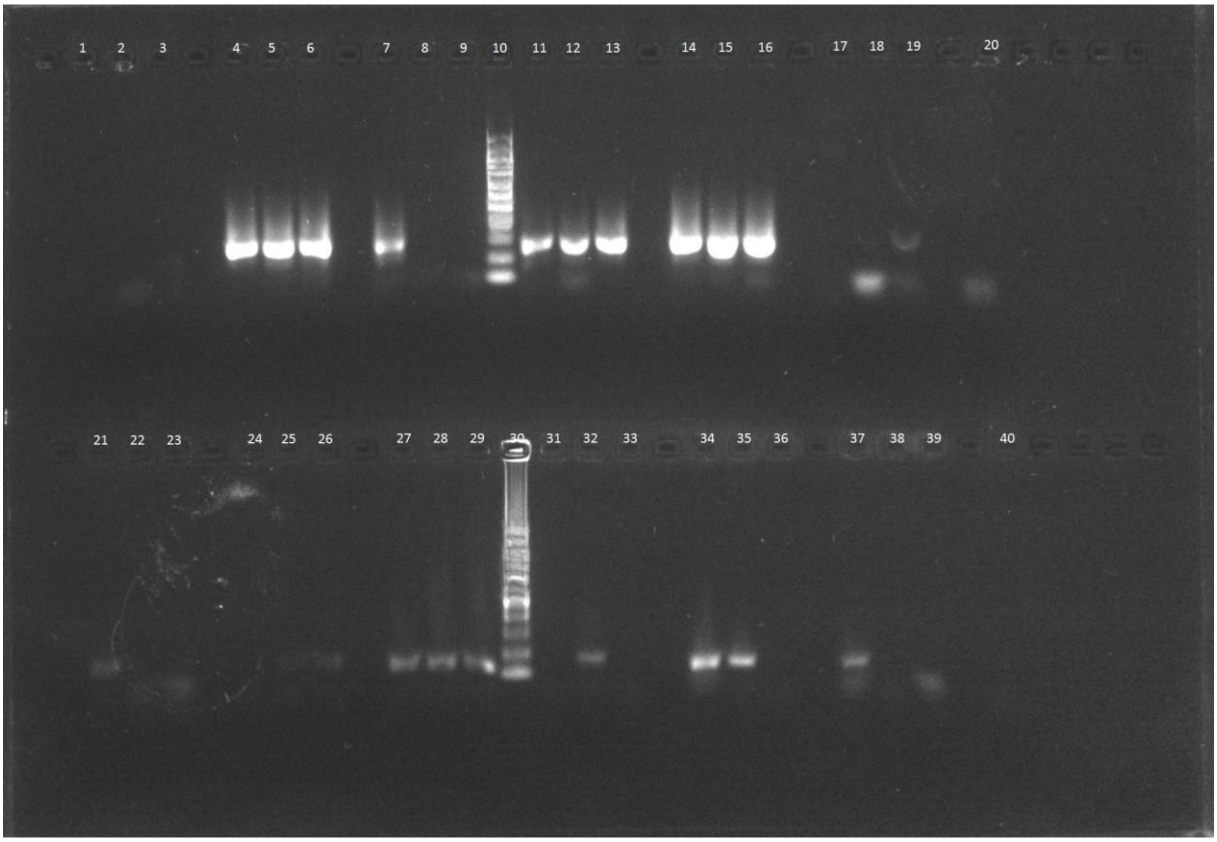

Figure 5: Plum – result of PCR assay with species specific primers: 1-3 kit 1, 4-6 kit 2, 7-9 kit 3, 10 DNA ladder, 11-13 kit 4, 14-16 kit 5, 17-19 CTAB protocol, 20 no template control. Apple: 21-23 kit 1, 24-26 kit 2, 27-29 kit 3, 30 DNA ladder, 31-33 kit 4, 34-36 kit 5, 37-39 CTAB protocol, 40 no template control.
