# Supplementary material for: A Comparative Study of Some Procedures for Isolation of Fruit DNA of Sufficient Quality for PCR-Based Assays
Source: Molecules. 2020 Sep 20;25(18):4317. doi: 10.3390/molecules25184317 (PMC7570663; doi:10.3390/molecules25184317)
Supplement: Supplementary file 1 [file molecules-25-04317-s001.zip › molecules-913289-supplementary-revised-2nd - original/molecules 913289/S5 bilberry qPCR.pdf]

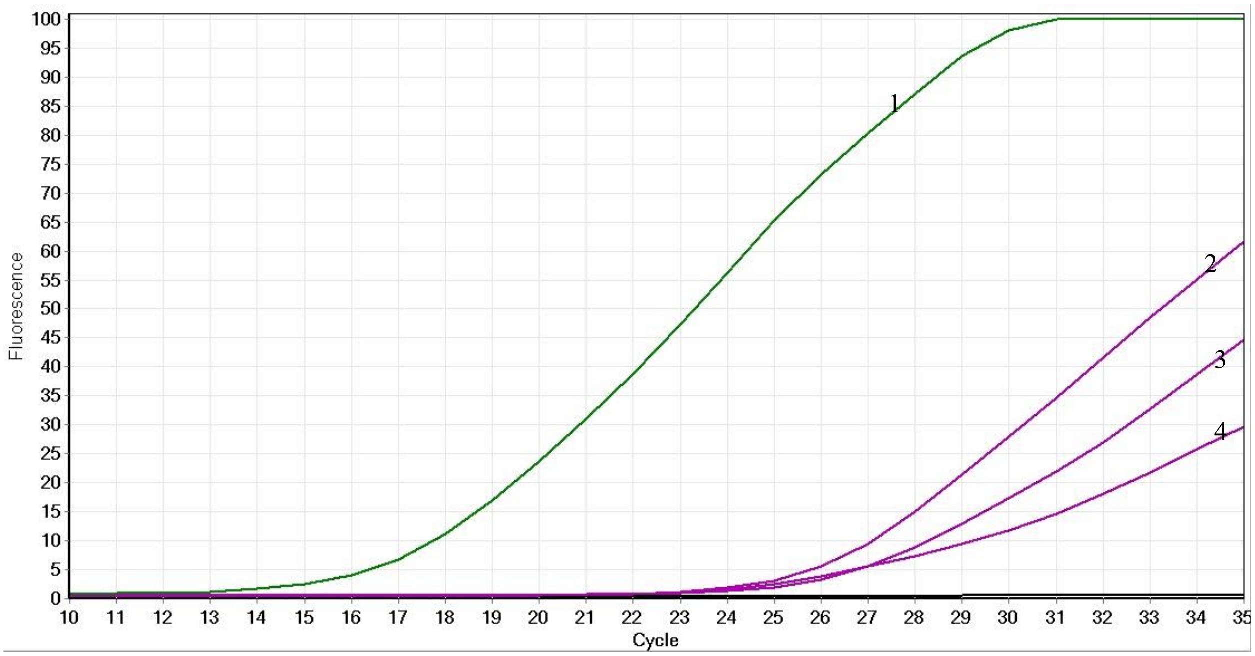

Figure 1: ITS2 amplification curves of blueberry DNA isolates obtained by kit 1 (Qiagen). 1 - positive control, 2-4 bilberry DNA isolates.

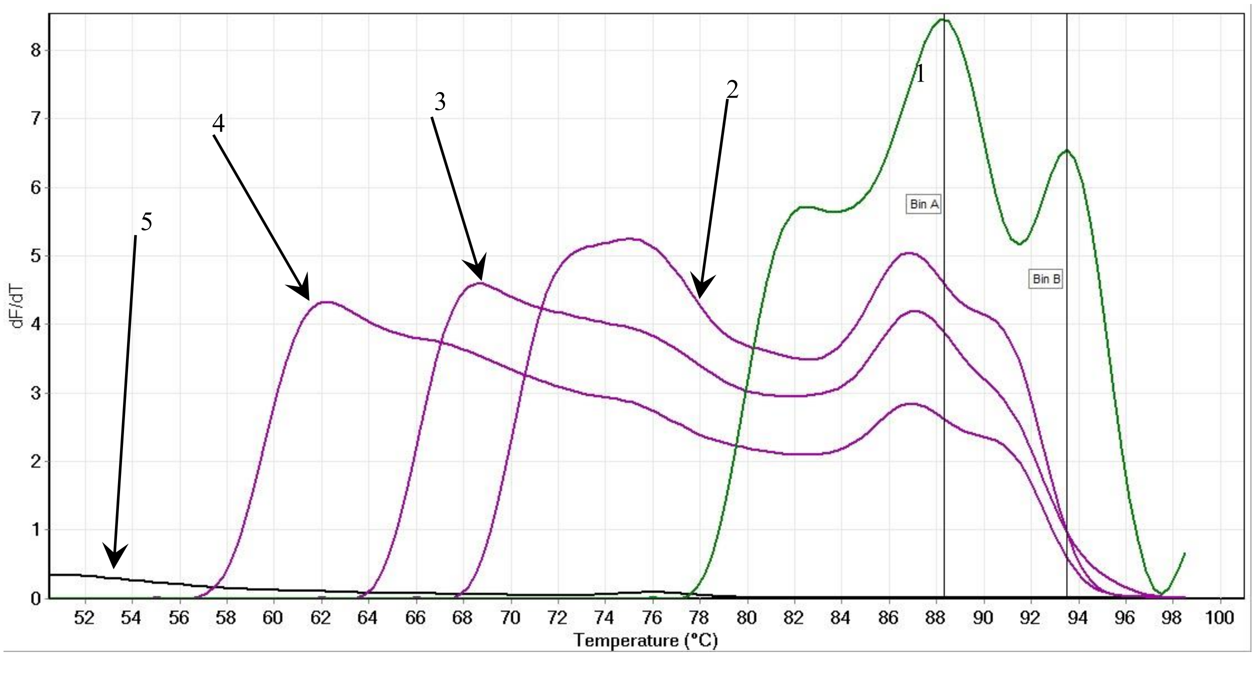

Figure 2: Melting curves of ITS2 amplicones of blueberry DNA isolates obtained by kit 1 (Qiagen). 1 - positive control, 2-4 bilberry DNA isolates, 5 - no template control.

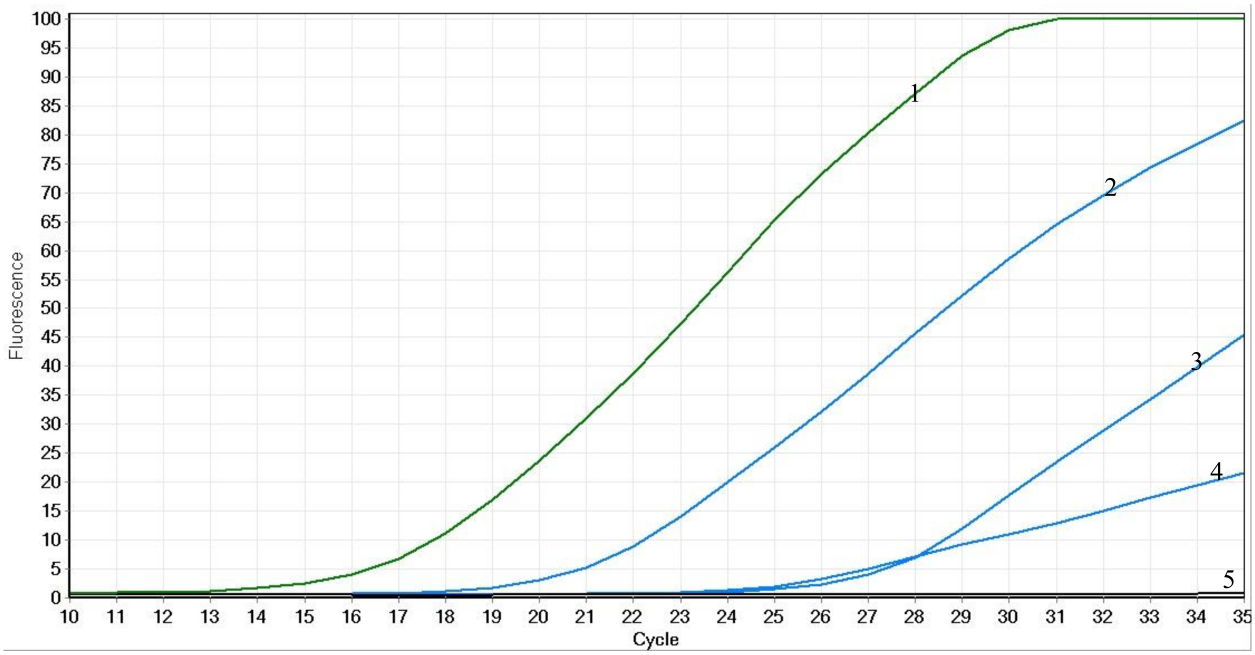

Figure 3: ITS2 amplification curves of blueberry DNA isolates obtained by kit 2 (Elisabeth Pharmacon). 1 - positive control, 2-4 bilberry DNA isolates, 5 - no template control.

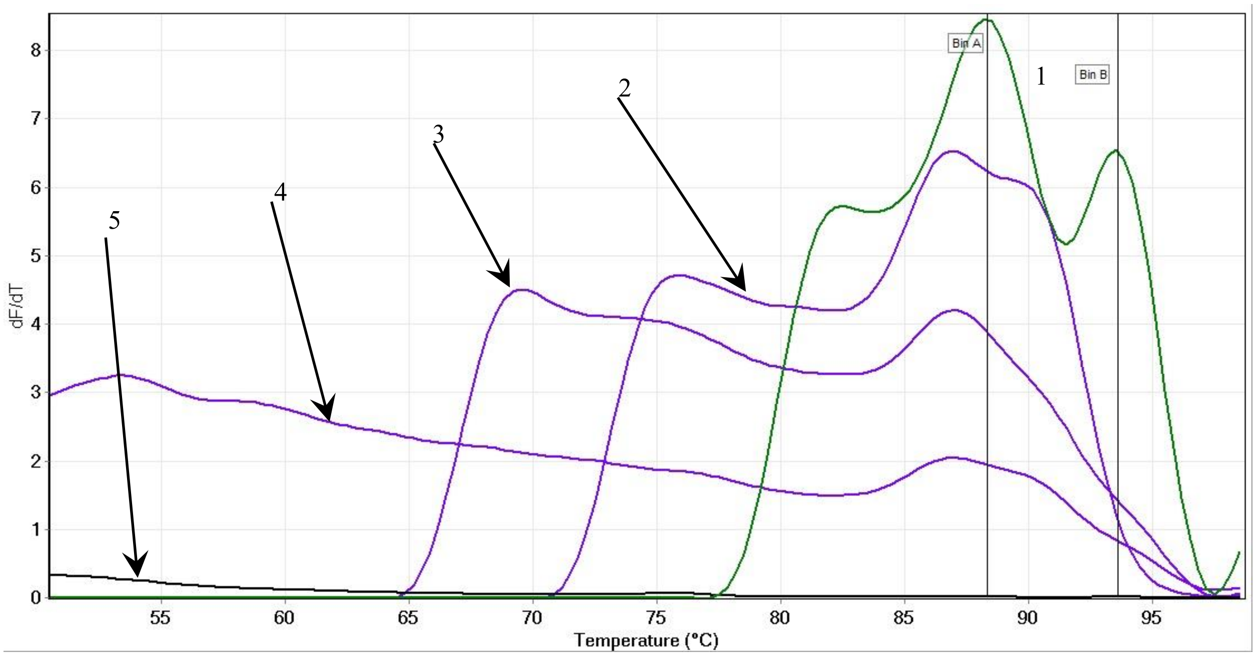

Figure 4: Melting curves of ITS2 amplicones of blueberry DNA isolates obtained by kit 2 (Elisabeth Pharmacon). 1- positive control, 2-4 bilberry DNA isolates, 5 - no template control

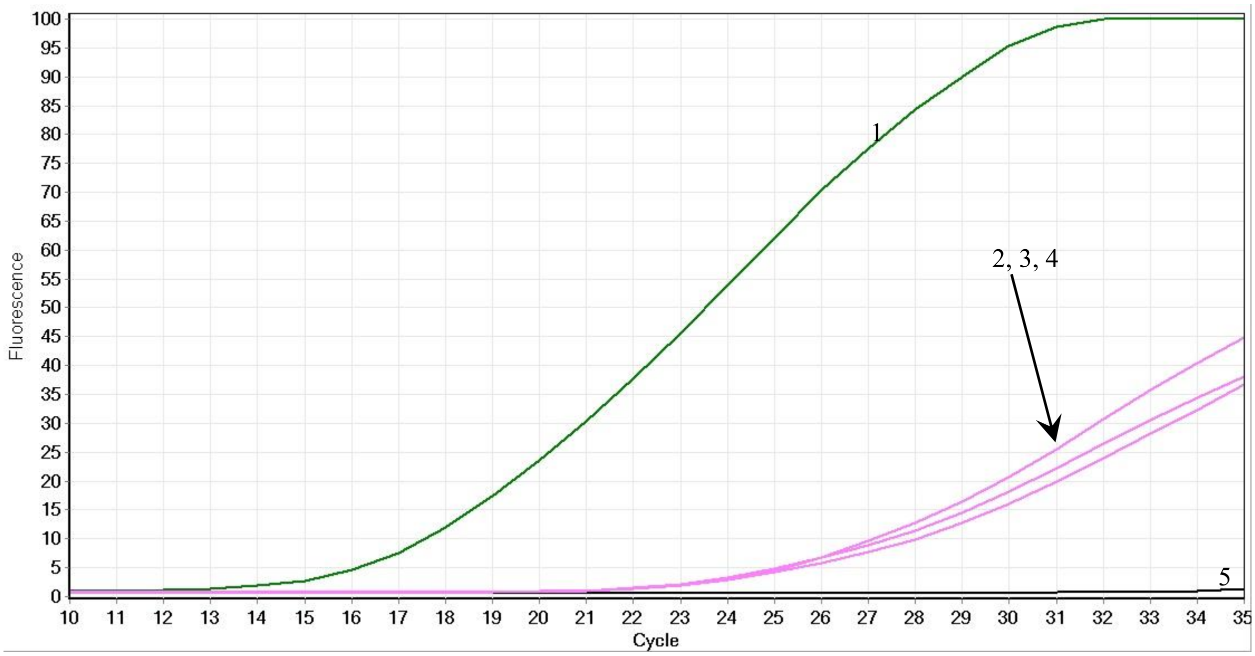

Figure 5: ITS2 amplification curves of blueberry DNA isolates obtained by kit 3 (Invitrogen). 1 - positive control, 2-4 bilberry DNA isolates, 5 - no template control

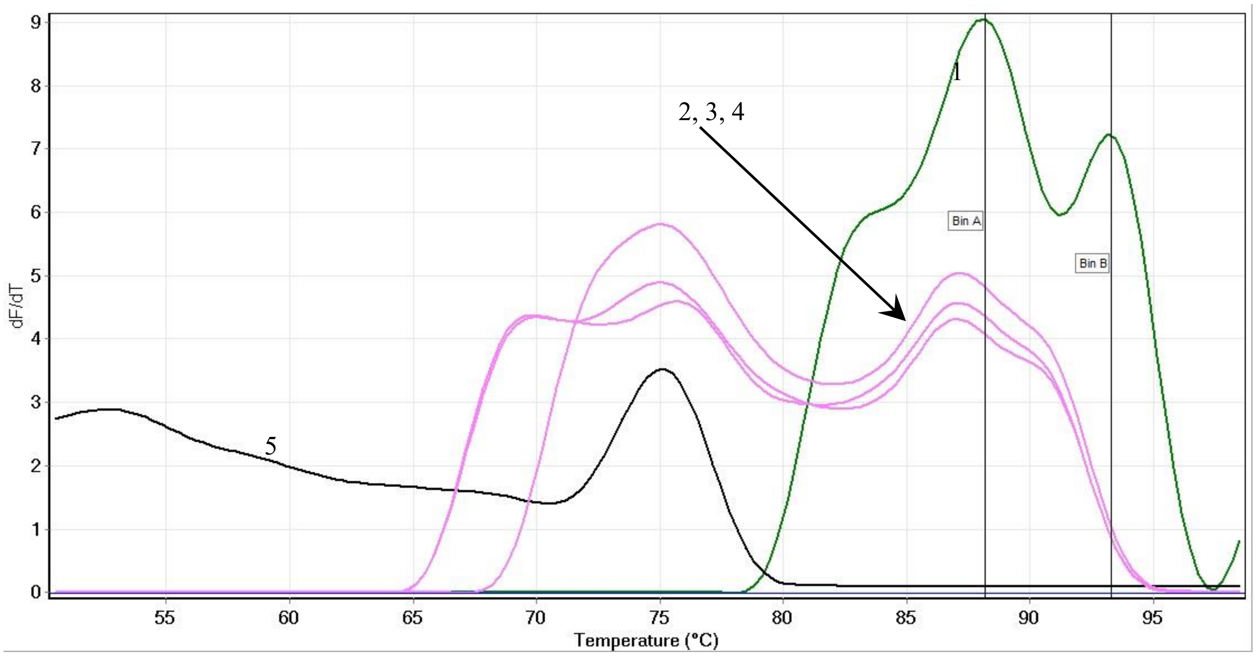

Figure 6: Melting curves of ITS2 amplicones of blueberry DNA isolates obtained by kit 3 (Invitrogen). 1 - positive control, 2-4 bilberry DNA isolates, 5 - no template control.

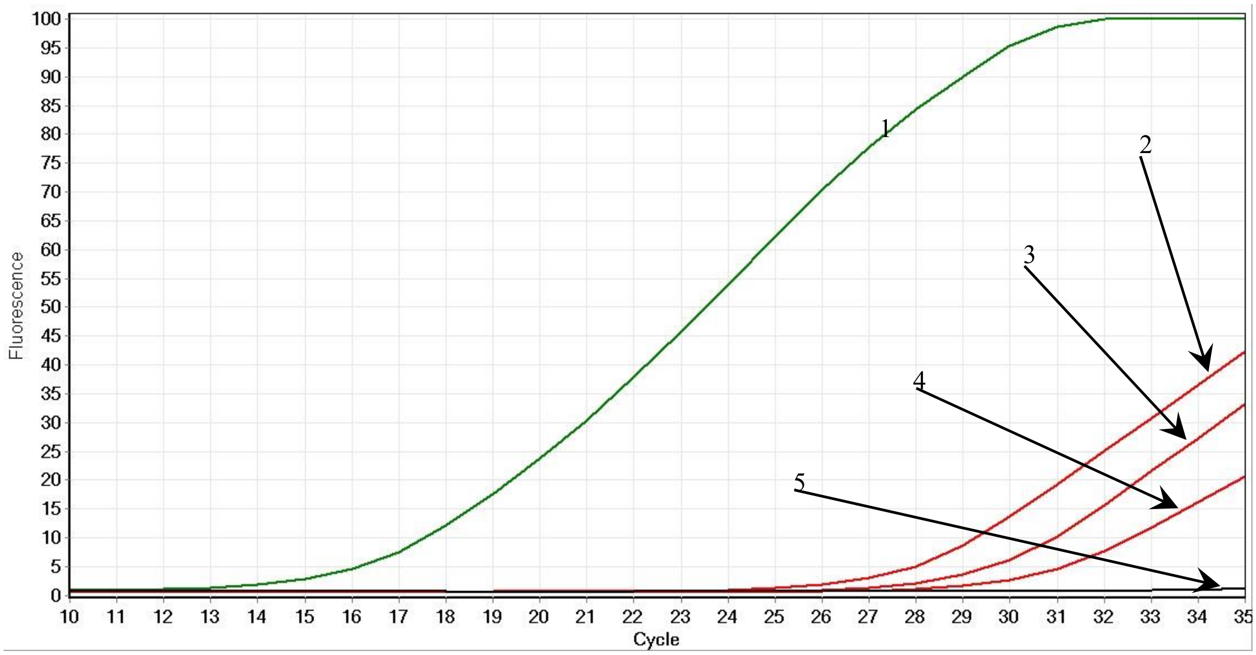

Figure 7: ITS2 amplification curves of blueberry DNA isolates obtained by kit 4 (Perkin-Elmer). 1 - positive control, 2-4 bilberry DNA isolates, 5 - no template control.

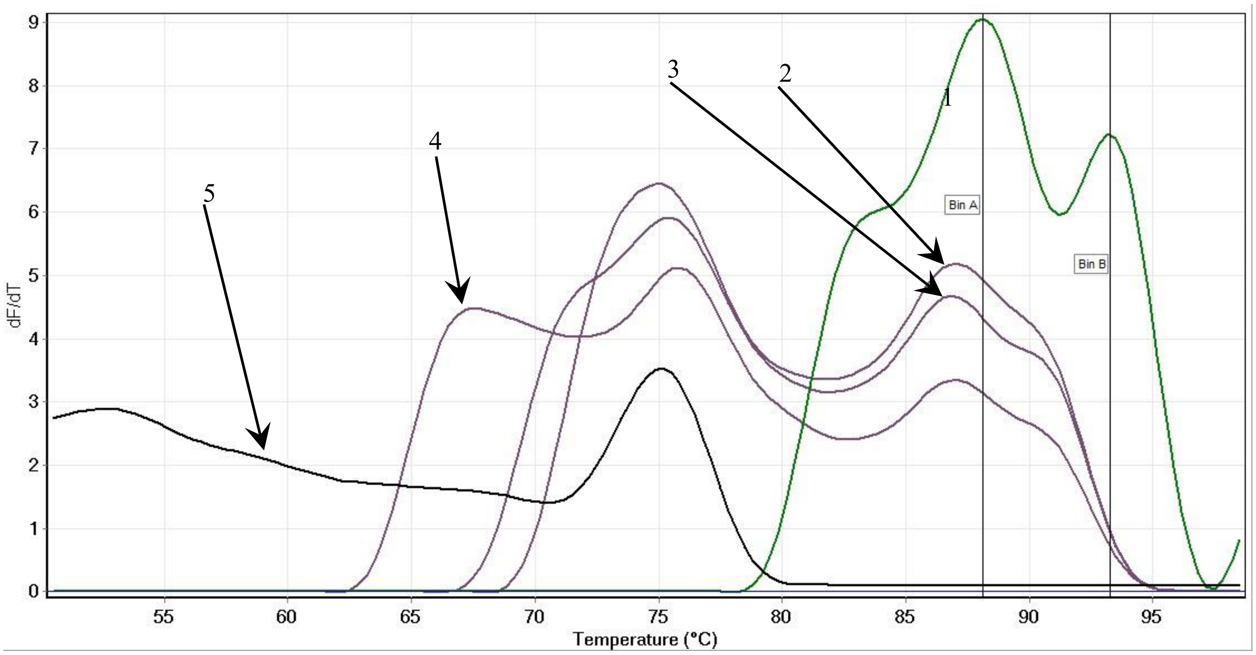

Figure 8: Melting curves of ITS2 amplicones of blueberry DNA isolates obtained by kit 4 (Perkin-Elmer). 1 - positive control, 2-4 bilberry DNA isolates, 5 - no template control.

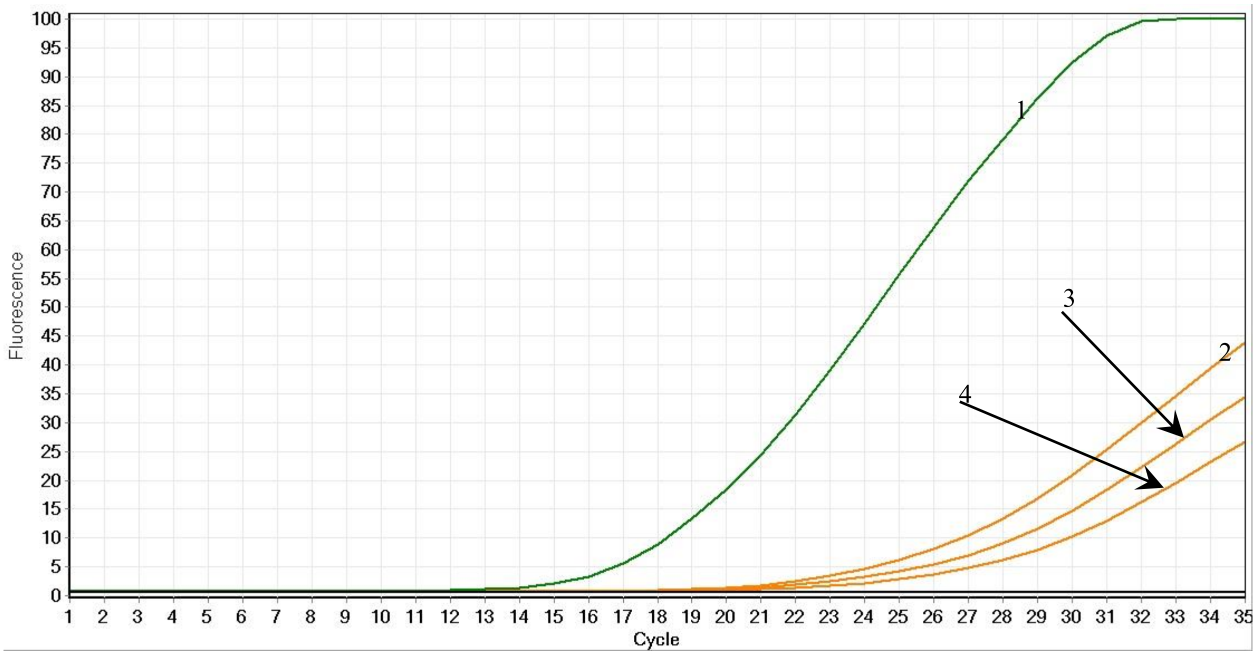

Figure 9: ITS2 amplification curves of blueberry DNA isolates obtained by kit 5 (Tools). 1- positive control, 2-4 bilberry DNA isolates.

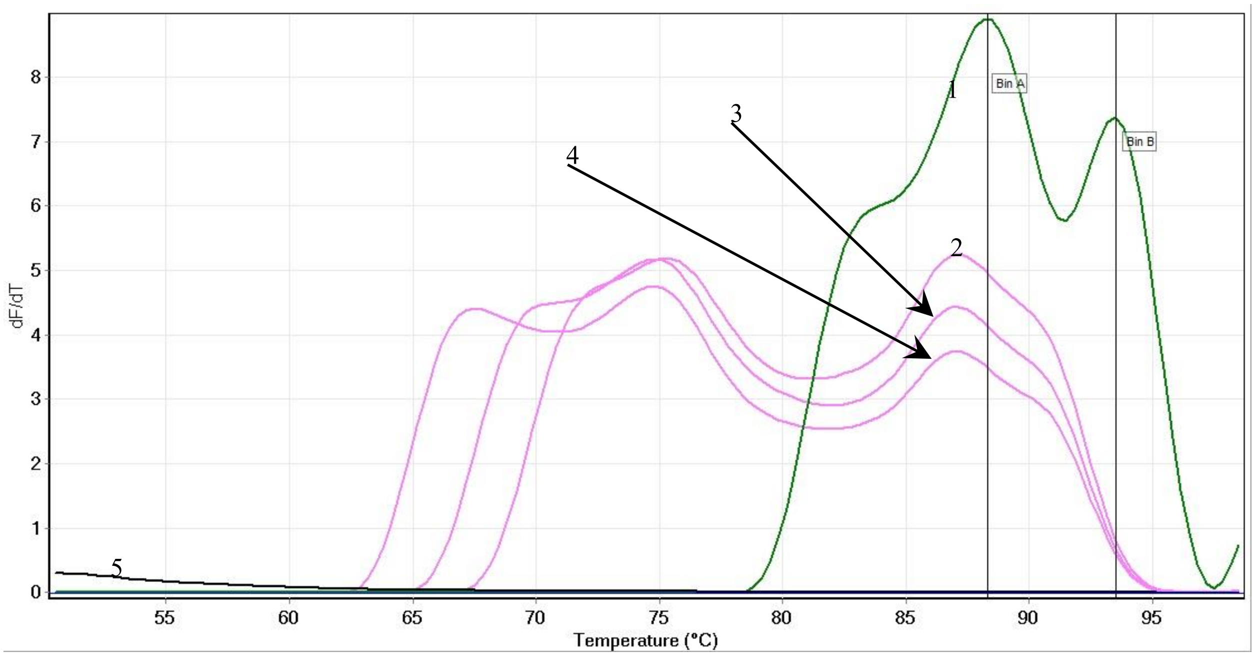

Figure 10: Melting curves of ITS2 amplicones of blueberry DNA isolates obtained by kit 5 (Tools). 1- positive control, 2-4 bilberry DNA isolates, 5 - no template control.

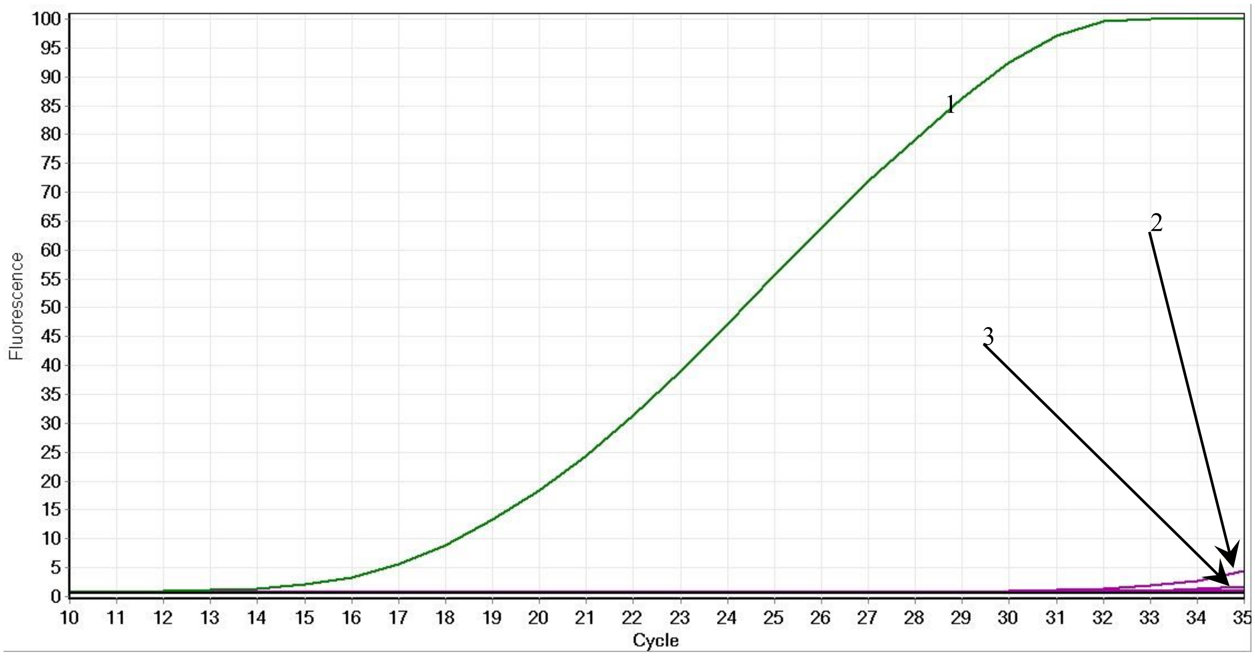

Figure 11: ITS2 amplification curves of blueberry DNA isolates obtained by the CTAB protocol. 1- positive control, 2-3 bilberry DNA isolates.

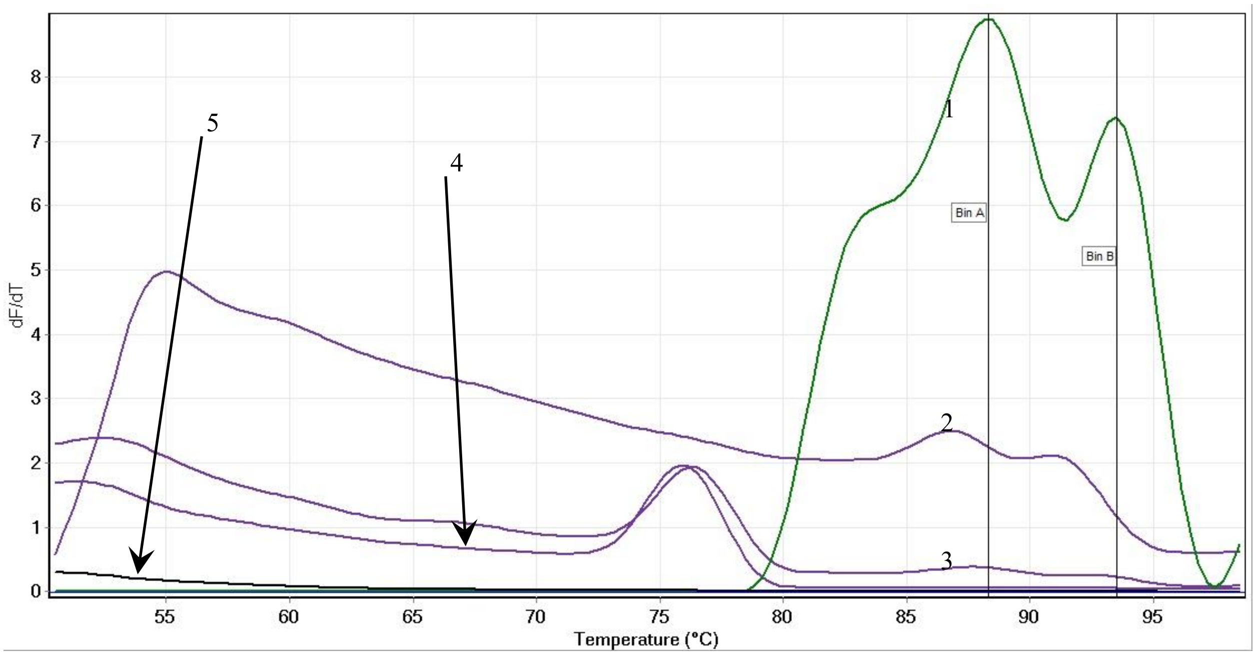

Figure 12: Melting curves of ITS2 amplicones of blueberry DNA isolates obtained by the CTAB protocol. 1 - positive control, 2-4 bilberry DNA isolates, 5 - no template control.

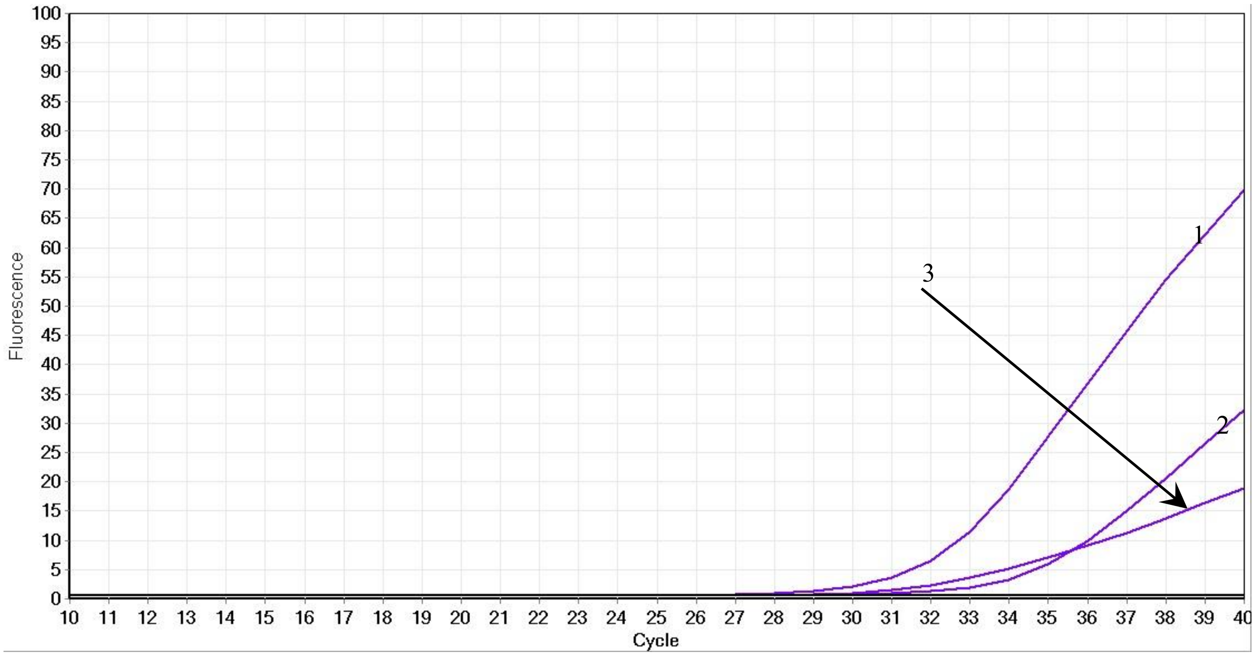

Figure 13: BHLH003 amplification curves of blueberry DNA isolates obtained by kit 1 (Qiagen). 1-3 bilberry DNA isolates.

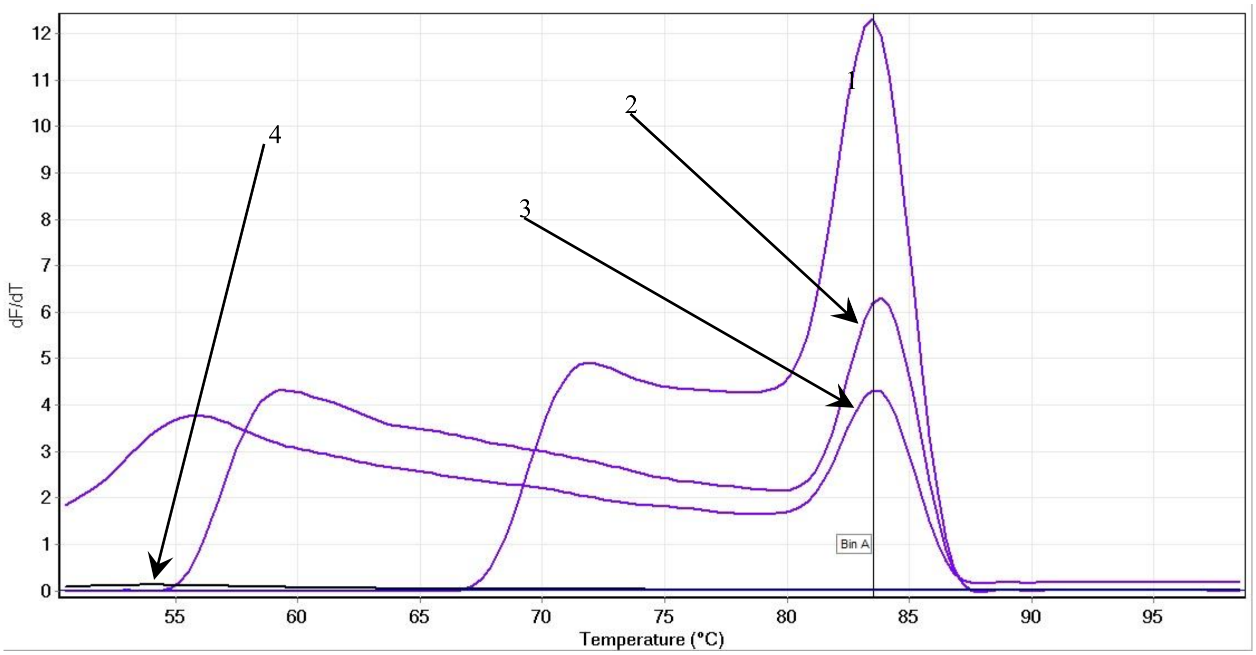

Figure 14: Melting curves of BHLH003 amplicones of blueberry DNA isolates obtained by kit 1 (Qiagen). 1-3 bilberry DNA isolates, 4 - no template control.

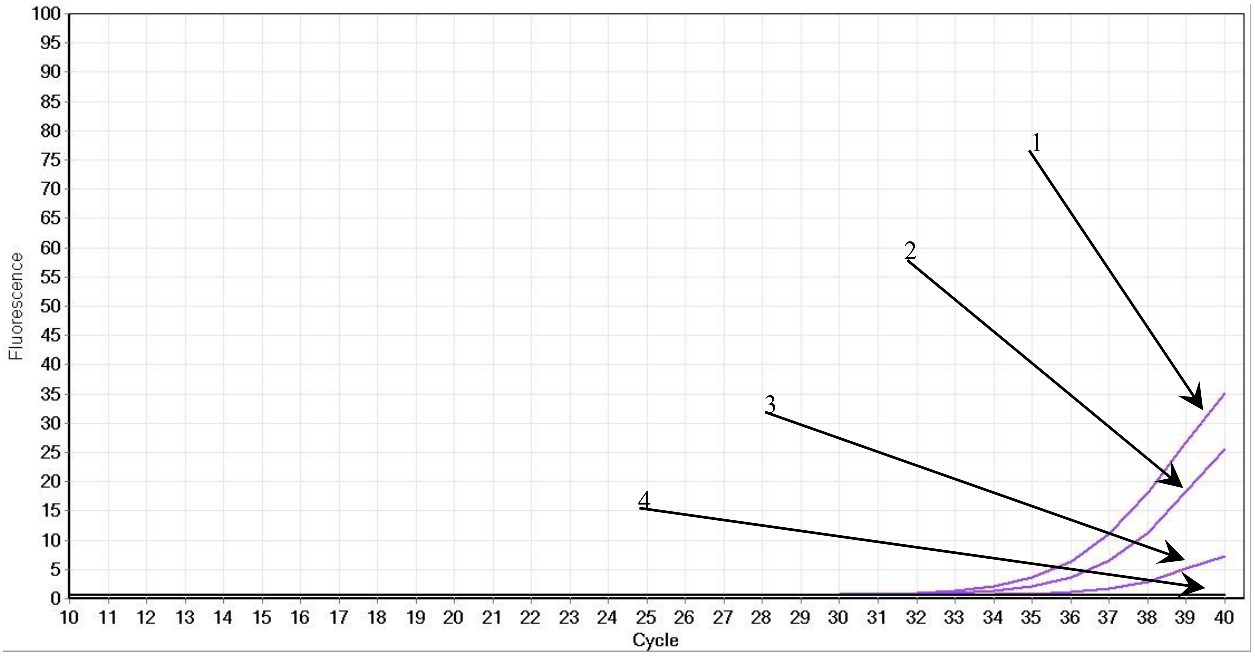

Figure 15: BHLH003 amplification curves of blueberry DNA isolates obtained by kit 2 (Elisabeth Pharmacon). 1-3 bilberry DNA isolates, 4 - no template control.

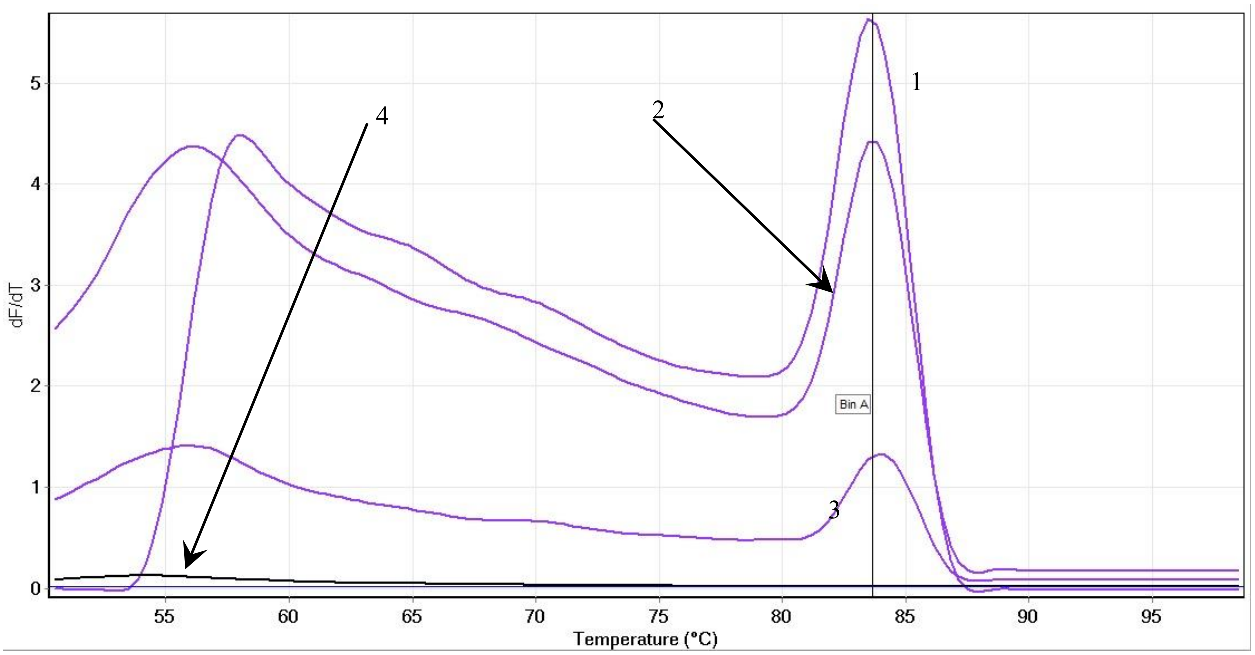

Figure 16: Melting curves of BHLH003 amplicones of blueberry DNA isolates obtained by kit 2 (Elisabeth Pharmacon). 1-3 bilberry DNA isolates, 4 - no template control.

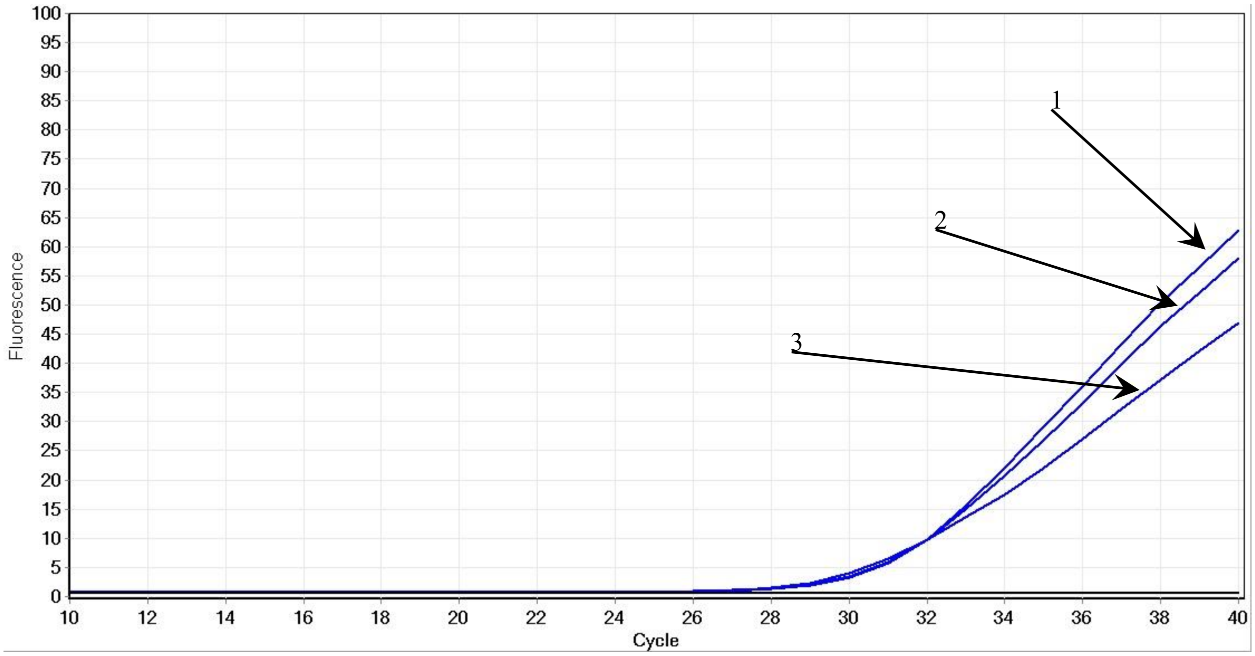

Figure 17: BHLH003 amplification curves of blueberry DNA isolates obtained by kit 3 (Invitrogen). 1-3 bilberry DNA isolates.

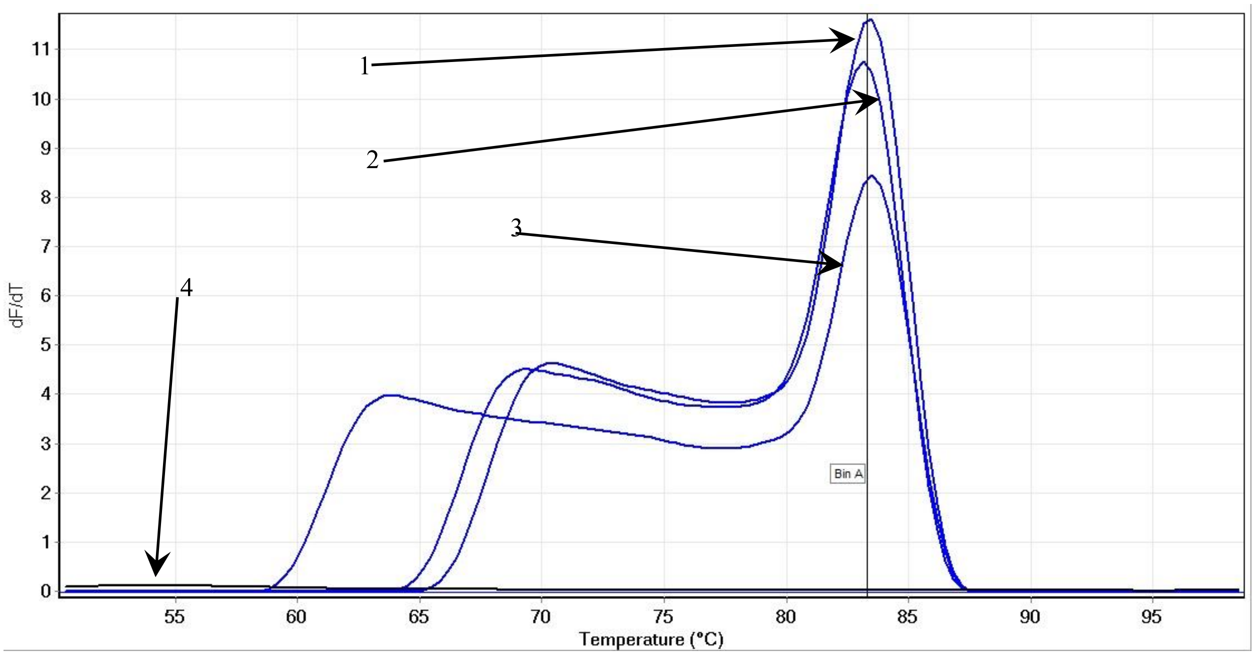

Figure 18: Melting curves of BHLH003 amplicones of blueberry DNA isolates obtained by kit 3 (Invitrogen). 1-3 bilberry DNA isolates, 4 - no template control.

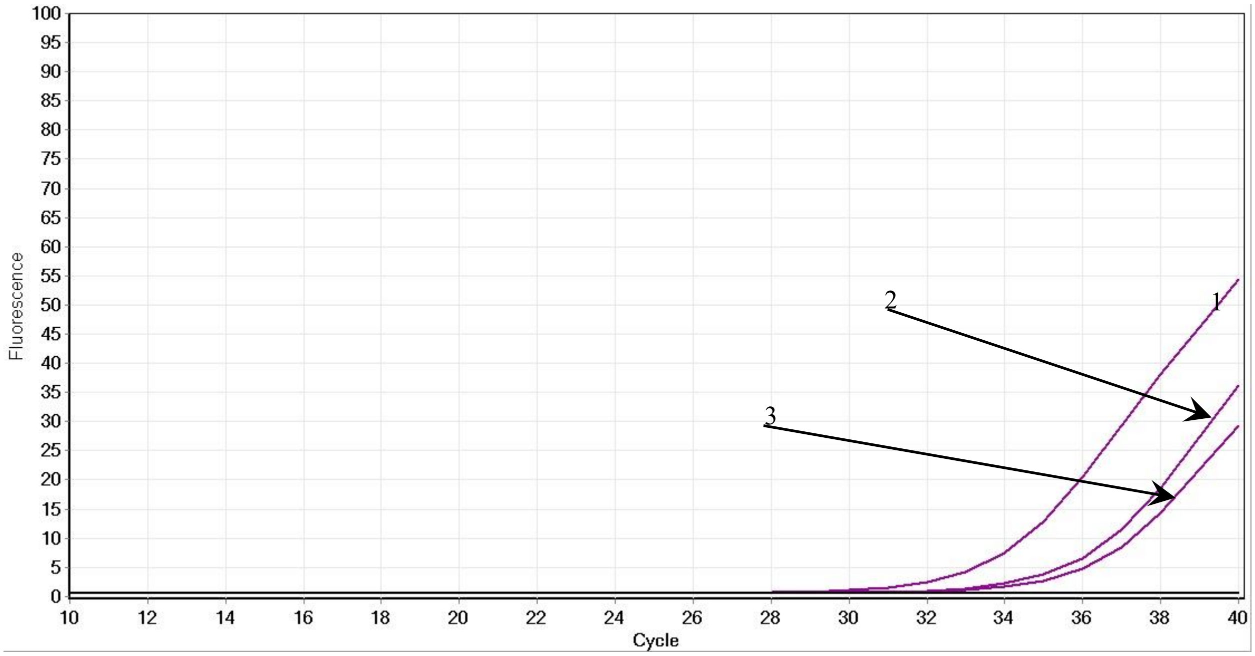

Figure 19: BHLH003 amplification curves of blueberry DNA isolates obtained by kit 4 (Perkin-Elmer). 1-3 bilberry DNA isolates.

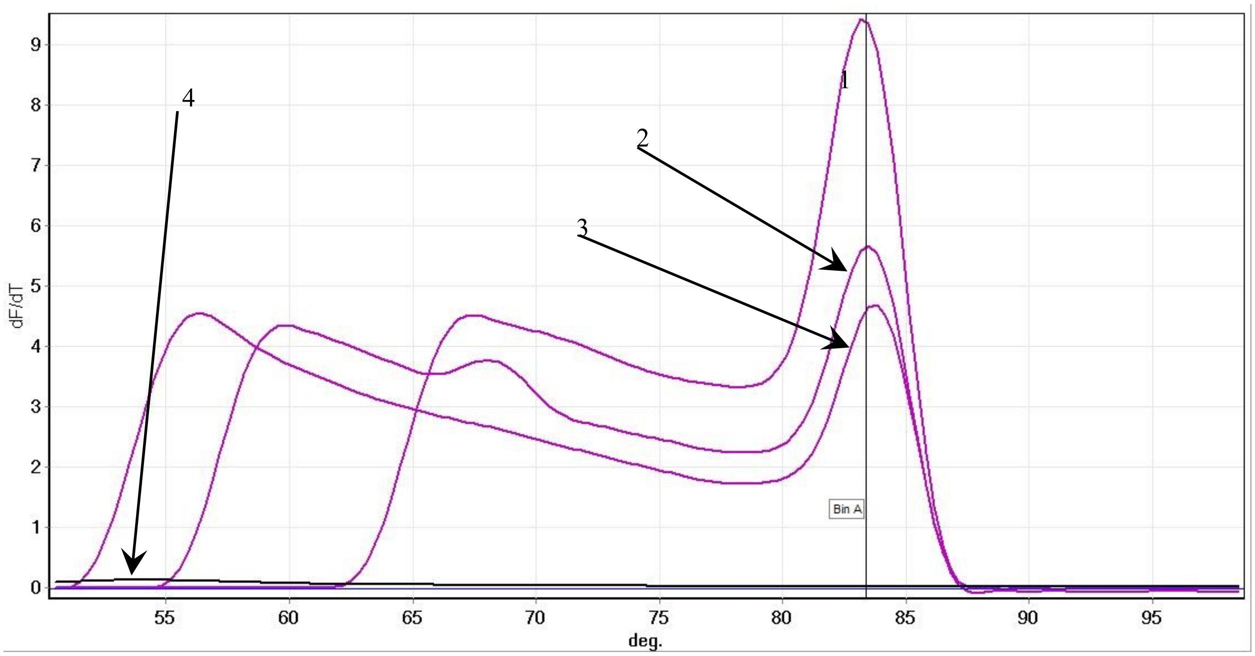

Figure 20: Melting curves of BHLH003 amplicones of blueberry DNA isolates obtained by kit 4 (Perkin-Elmer). 1-3 bilberry DNA isolates, 4 - no template control.

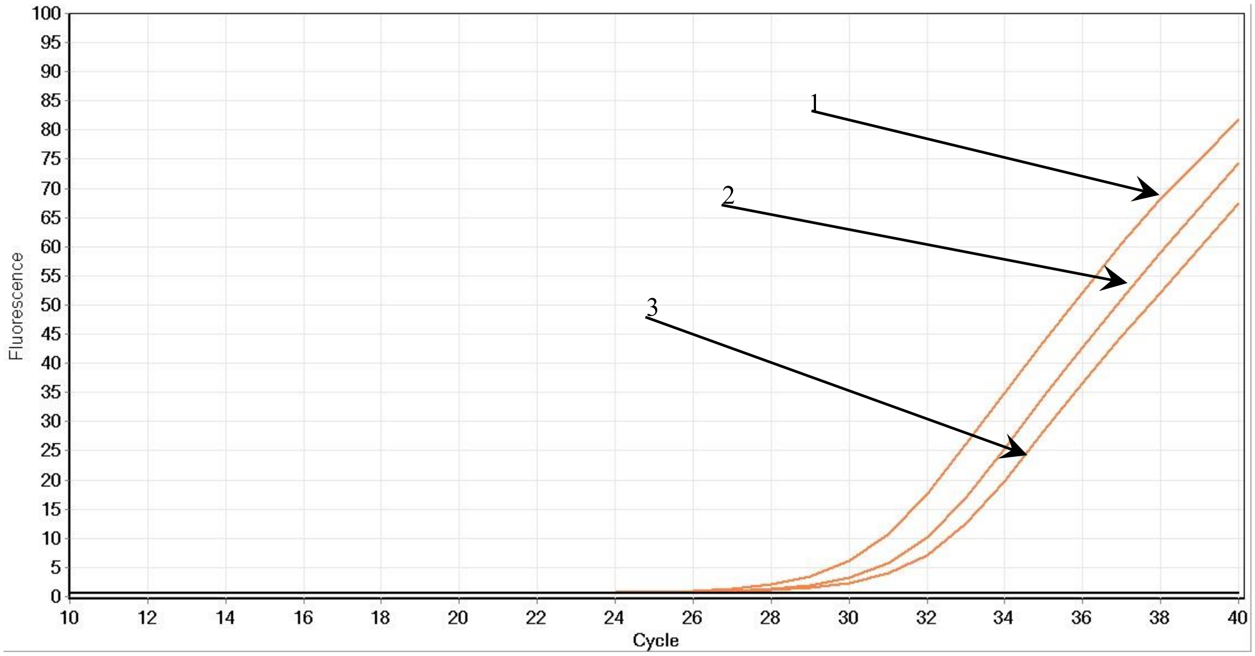

Figure 21: BHLH003 amplification curves of blueberry DNA isolates obtained by kit 5 (Tools). 1-3 bilberry DNA isolates.

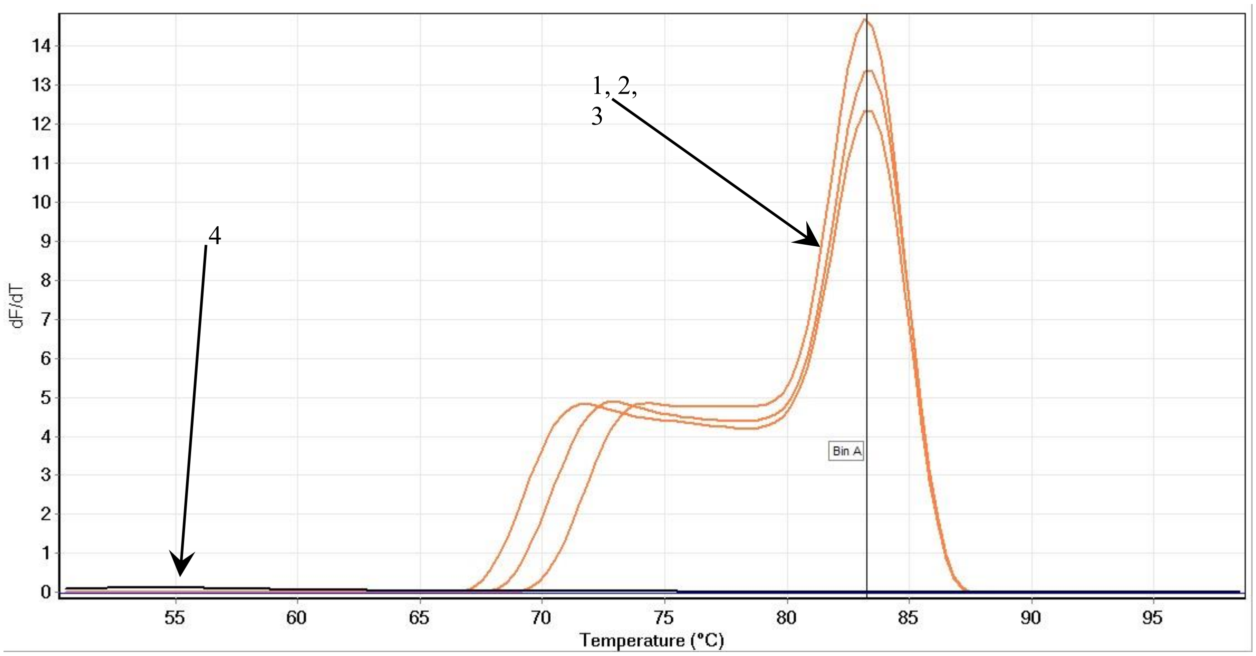

Figure 22: Melting curves of BHLH003 amplicones of blueberry DNA isolates obtained by kit 5 (Tools). 1-3 bilberry DNA isolates, 4 - no template control.

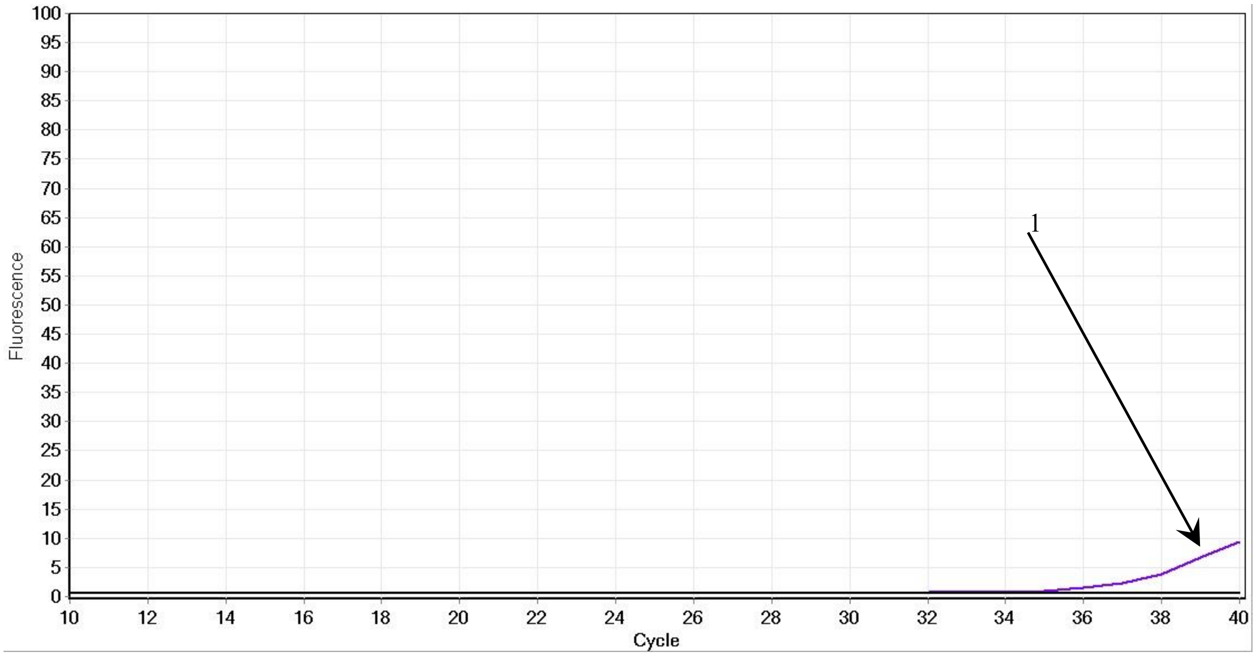

Figure 23: BHLH003 amplification curves of blueberry DNA isolates obtained by the CTAB protocol. 1 - bilberry DNA isolate

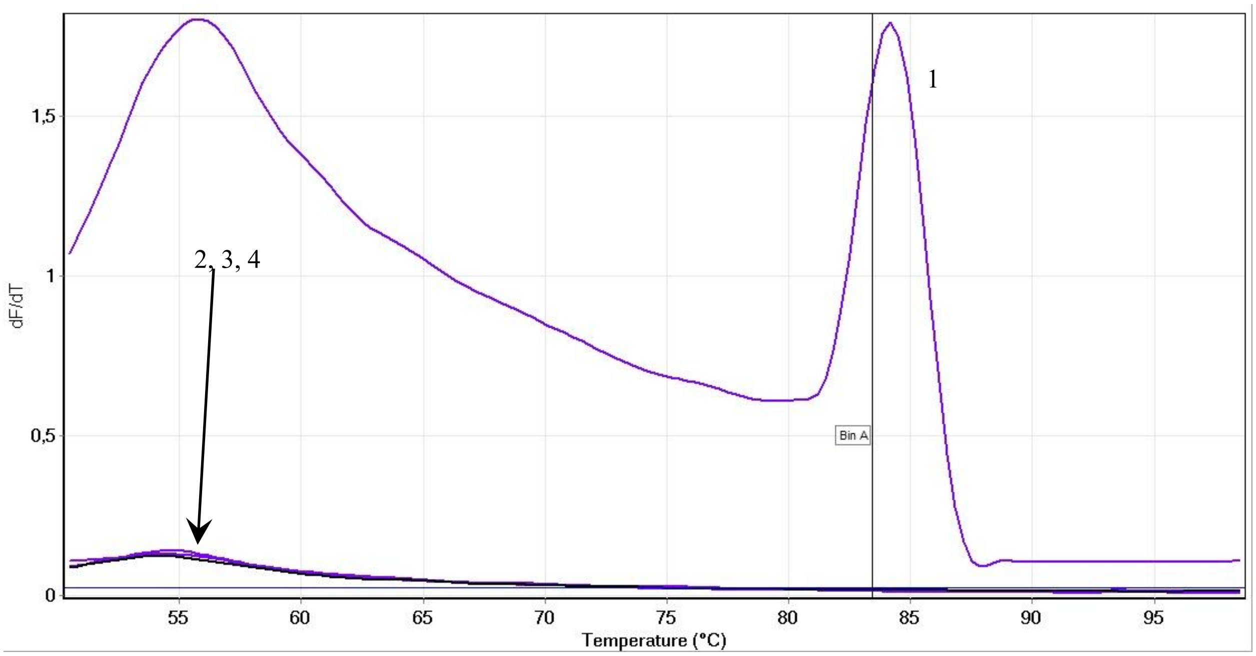

Figure 24:Melting curves of BHLH003 amplicones of blueberry DNA isolates obtained by the CTAB protocol. 1-3 bilberry DNA isolates, 4 - no template control.
