# Supplementary material for: A Comparative Study of Some Procedures for Isolation of Fruit DNA of Sufficient Quality for PCR-Based Assays
Source: Molecules. 2020 Sep 20;25(18):4317. doi: 10.3390/molecules25184317 (PMC7570663; doi:10.3390/molecules25184317)
Supplement: Supplementary file 1 [file molecules-25-04317-s001.zip › molecules-913289-supplementary-revised-2nd - original/molecules 913289/S9 strawberry qPCR.pdf]

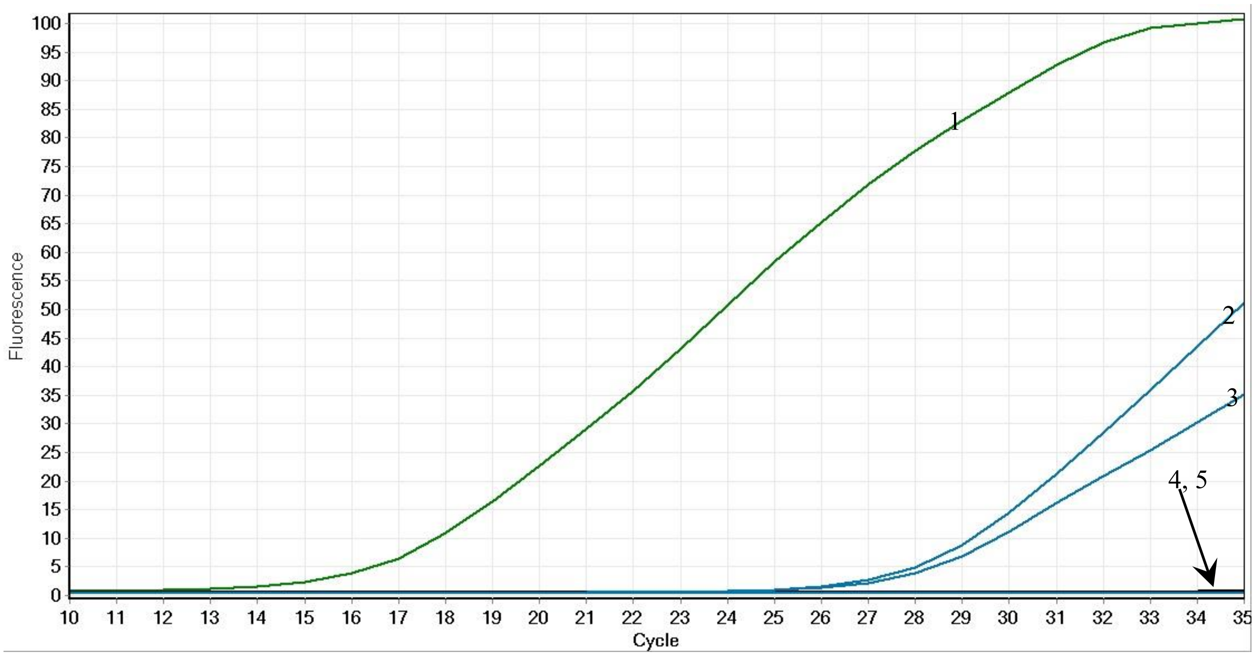

Figure 1: ITS2 amplification curves of strawberry DNA isolates obtained by kit 1 (Qiagen). 1 - positive control, 2-4 strawberry DNA isolates, 5 - no template control.

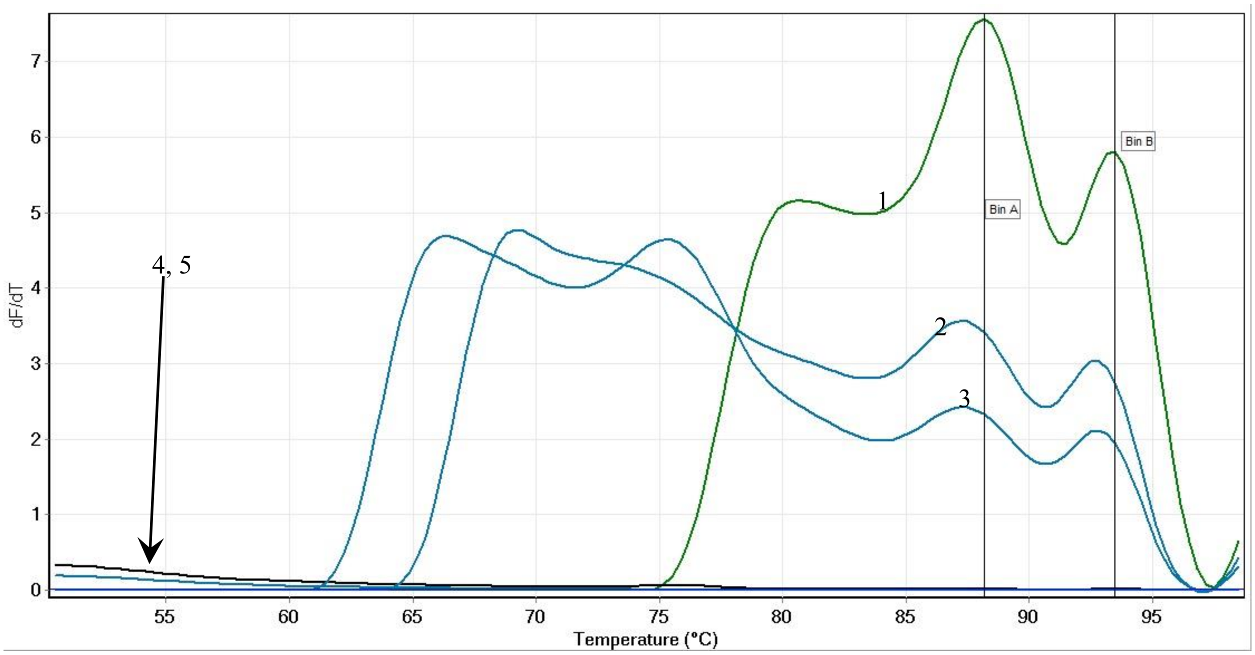

Figure 2: Melting curves of ITS2 amplicons of strawberry DNA isolates obtained by kit 1 (Qiagen). 1 - positive control, 2-4 strawberry DNA isolates, 5 - no template control.

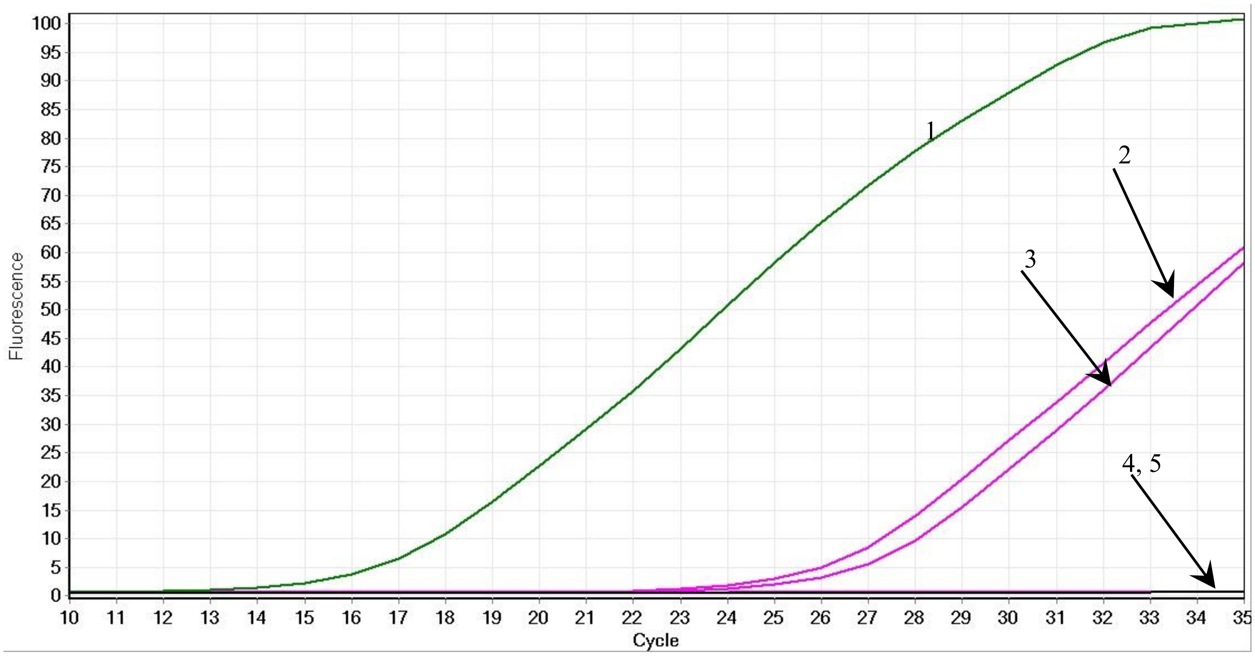

Figure 3: ITS2 amplification curves of strawberry DNA isolates obtained by kit 2 (Elisabeth Pharmacon). 1 - positive control, 2-4 strawberry DNA isolates, 5 - no template control.

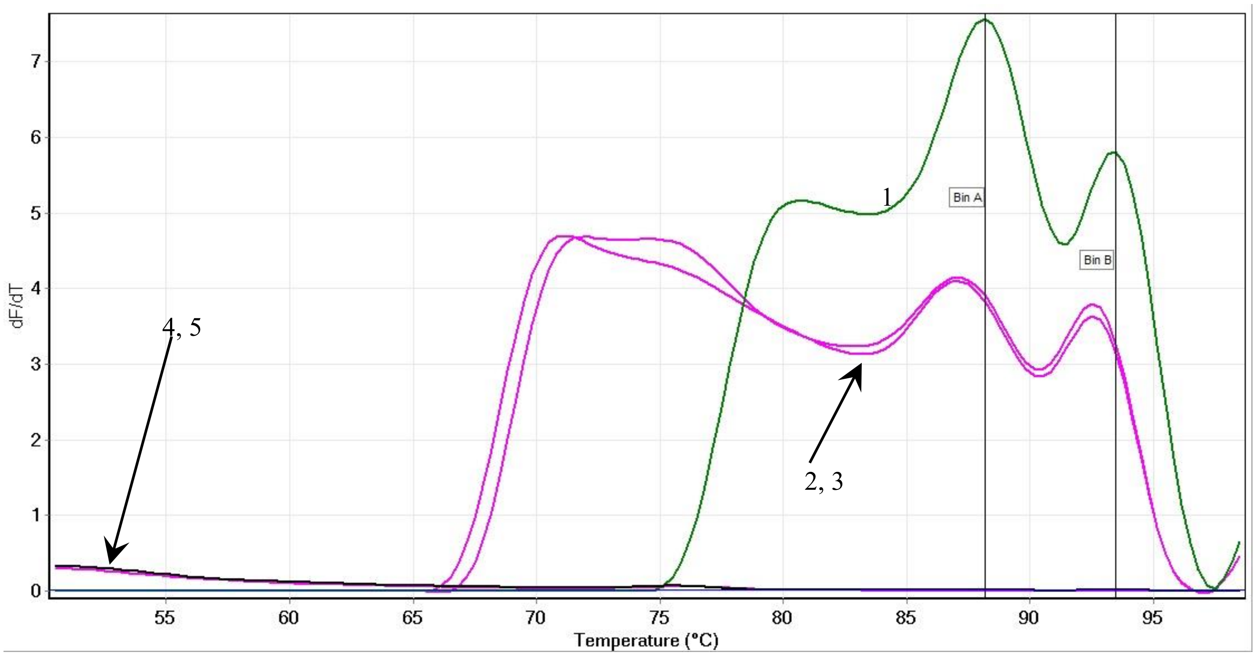

Figure 4: Melting curves of ITS2 amplicons of strawberry DNA isolates obtained by kit 2 (Elisabeth Pharmacon.) 1 - positive control, 2-4 strawberry DNA isolates, 5 - no template control.

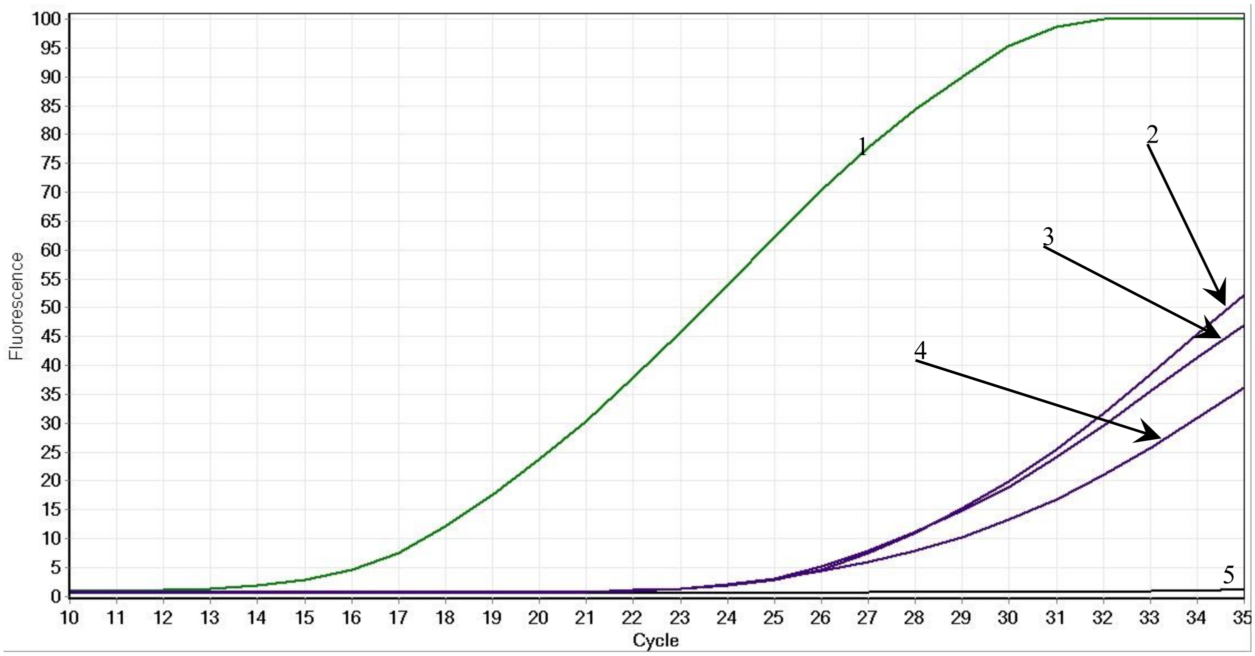

Figure 5: ITS2 amplification curves of strawberry DNA isolates obtained by kit 3 (Invitrogen). 1 - positive control, 2-4 strawberry DNA isolates, 5 - no template control.

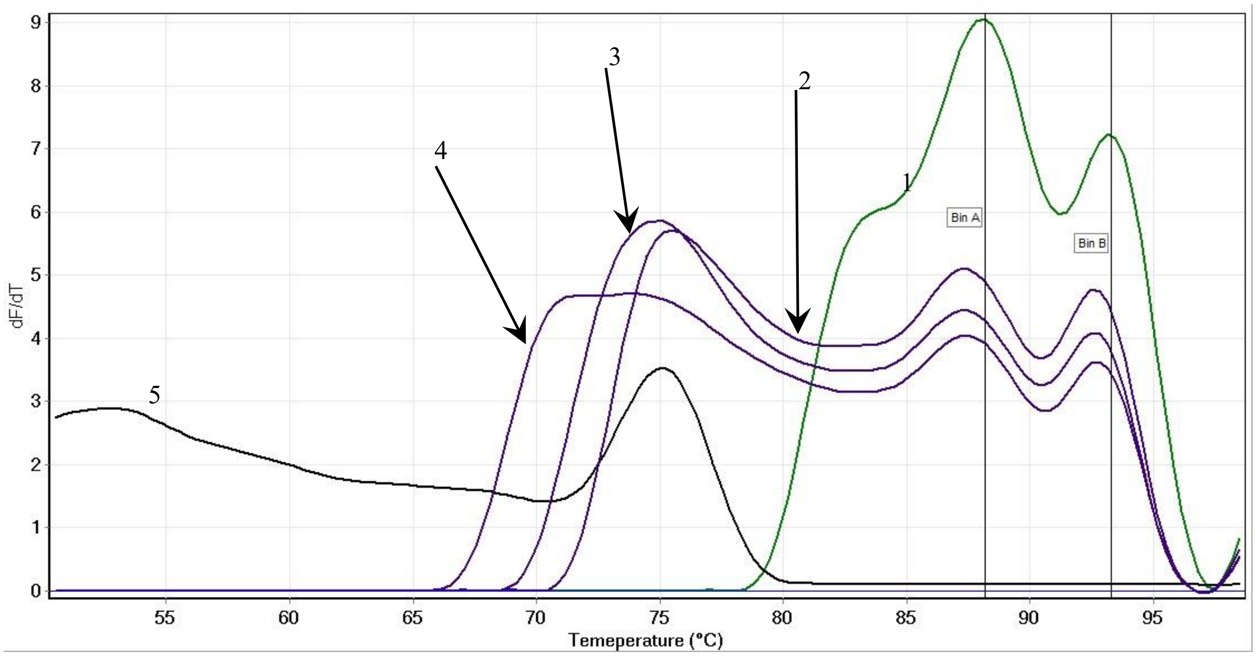

Figure 6: Melting curves of ITS2 amplicons of strawberry DNA isolates obtained by kit 3 (Invitrogen). 1 - positive control, 2-4 strawberry DNA isolates, 5 - no template control.

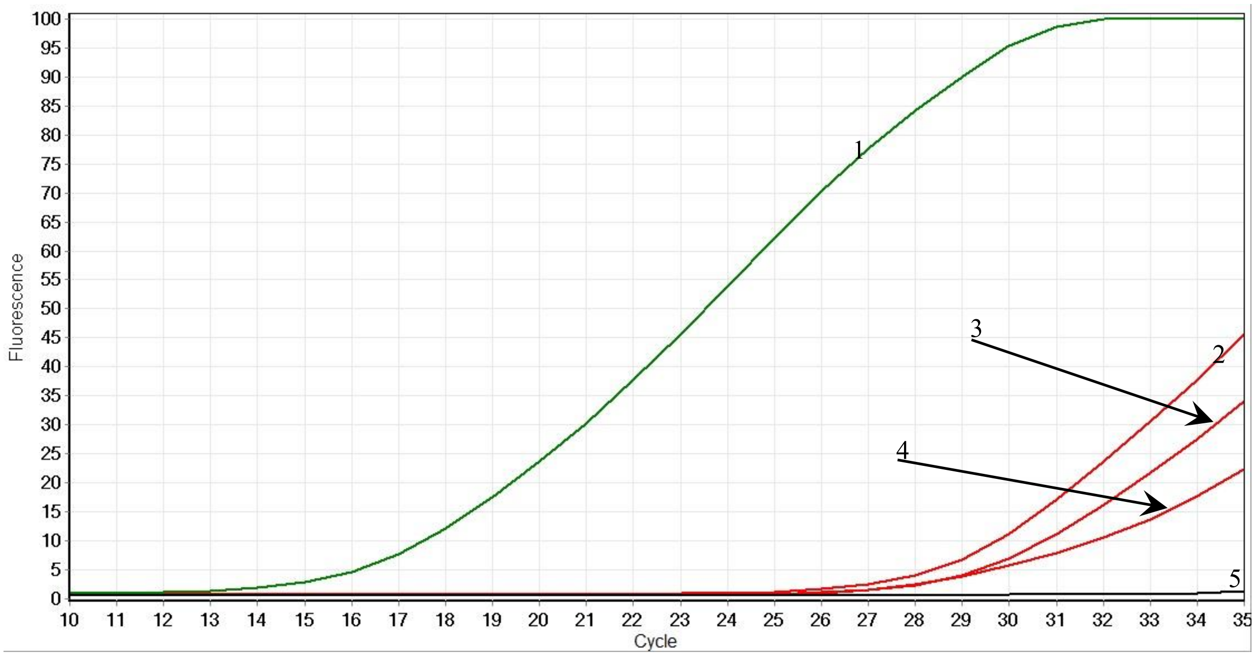

Figure 7: ITS2 amplification curves of strawberry DNA isolates obtained by kit 4 (Perkin-Elmer). 1 - positive control, 2-4 strawberry DNA isolates, 5 no template control.

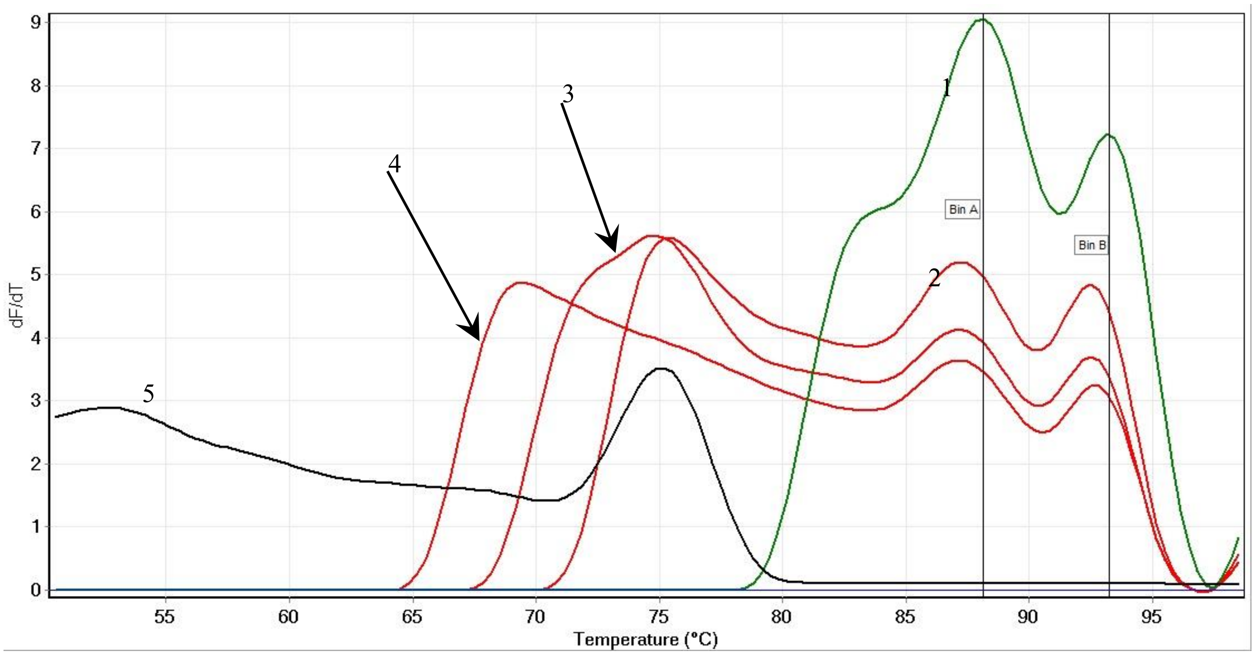

Figure 8: Melting curves of ITS2 amplicons of strawberry DNA isolates obtained by kit 4 (Perkin-Elmer). 1 - positive control, 2-4 strawberry DNA isolates, 5 - no template control.

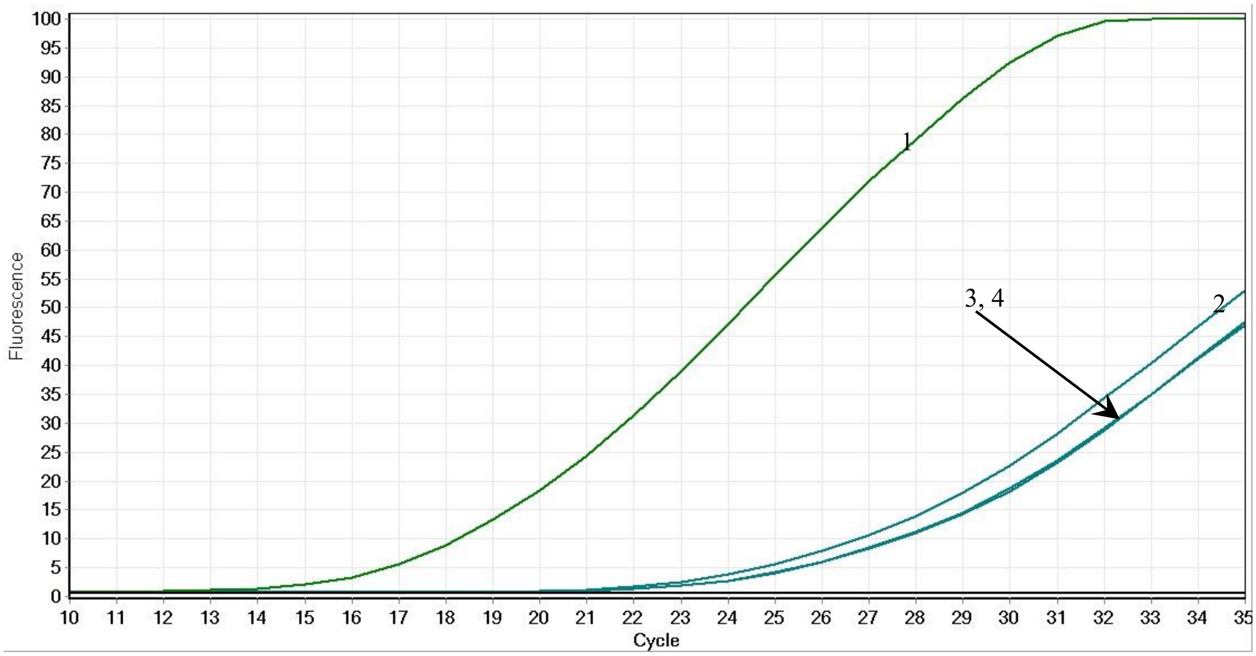

Figure 9: ITS2 amplification curves of strawberry DNA isolates obtained by kit 5 (Tools). 1 - positive control, 2-4 strawberry DNA isolates, 5 - no template control.

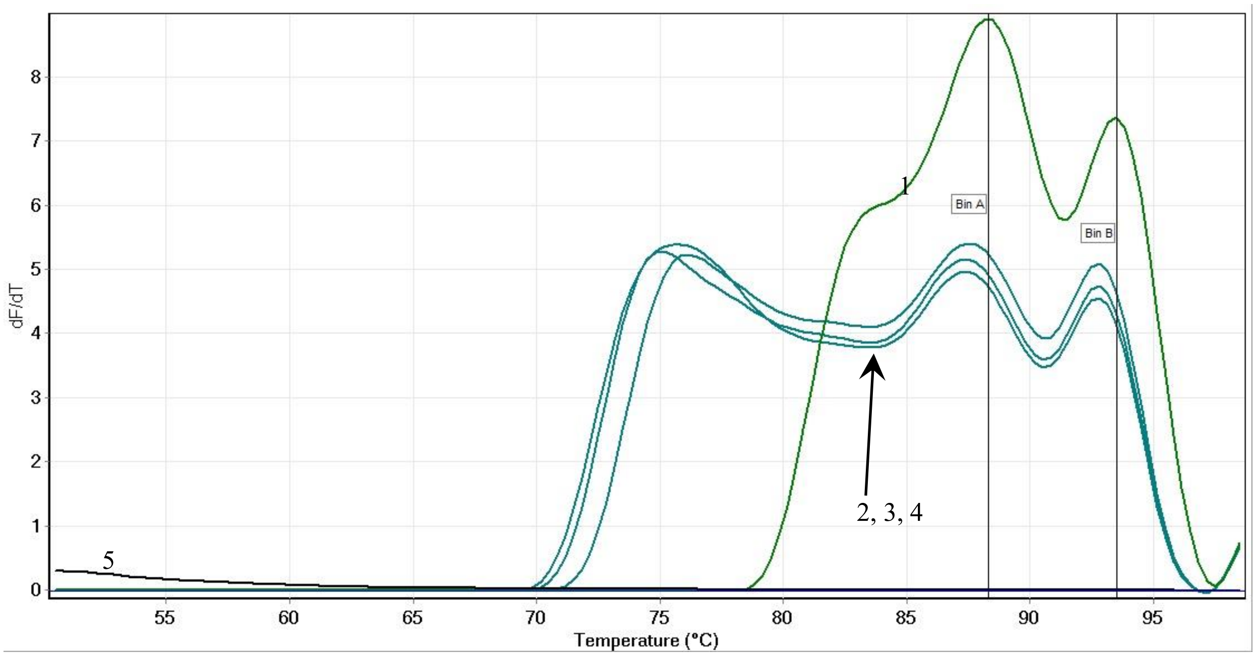

Figure 10: Melting curves of ITS2 amplicons of strawberry DNA isolates obtained by kit 5 (Tools). 1 - positive control, 2-4 strawberry DNA isolates, 5 - no template control.

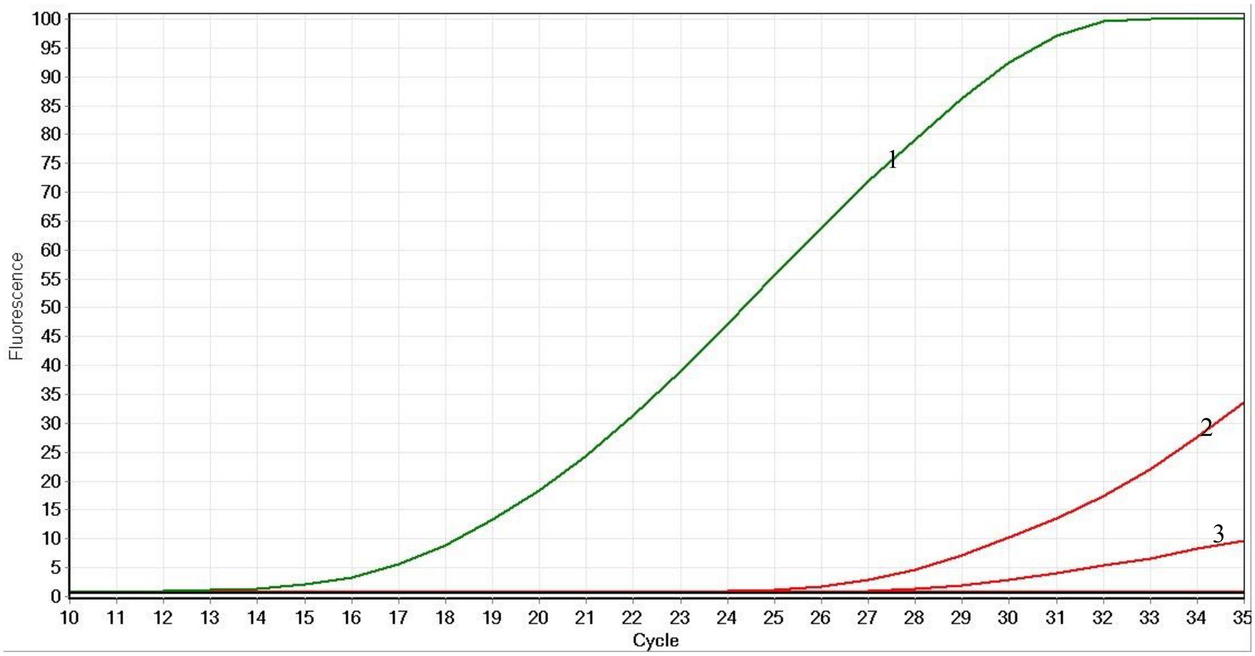

Figure 11: ITS2 amplification curves of strawberry DNA isolates obtained by the CTAB protocol. 1 - positive control, 2, 3 - strawberry DNA isolates.

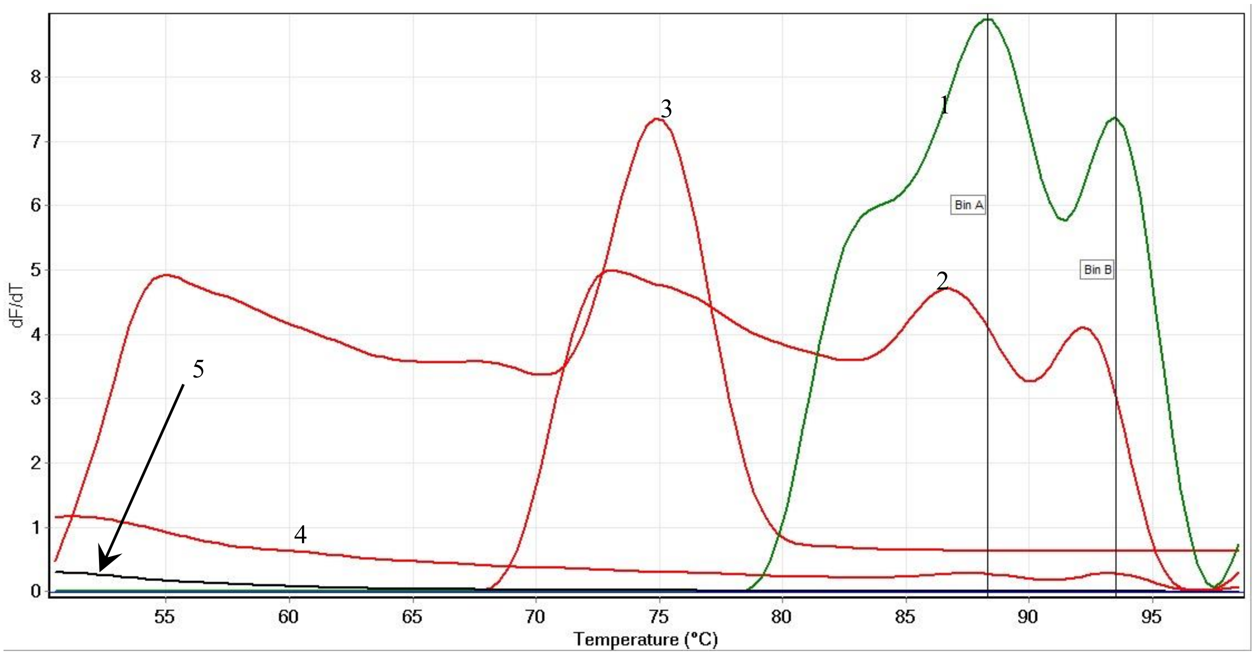

Figure 12: Melting curves of ITS2 amplicons of strawberry DNA isolates obtained by the CTAB protocol. 1 - positive control, 2-4 strawberry DNA isolates, 5 - no template control.

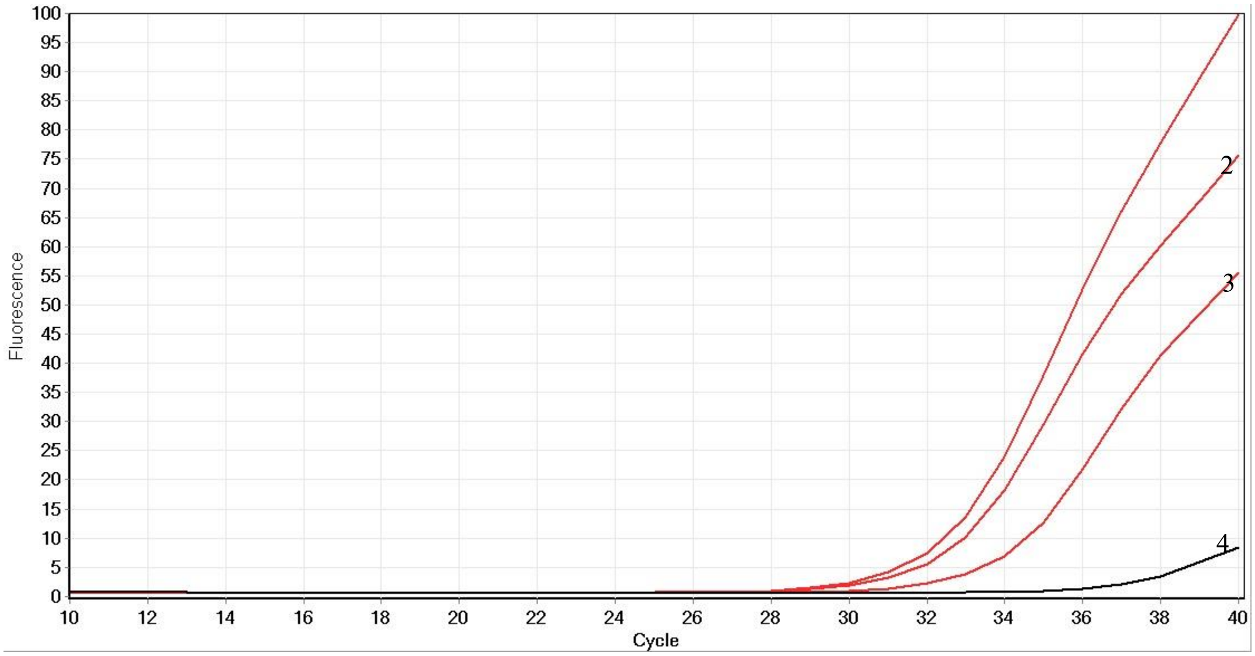

Figure 13: APX1B amplification curves of strawberry DNA isolates obtained by kit 1(Qiagen). 1-3 strawberry DNA isolates, 4 - no template control.

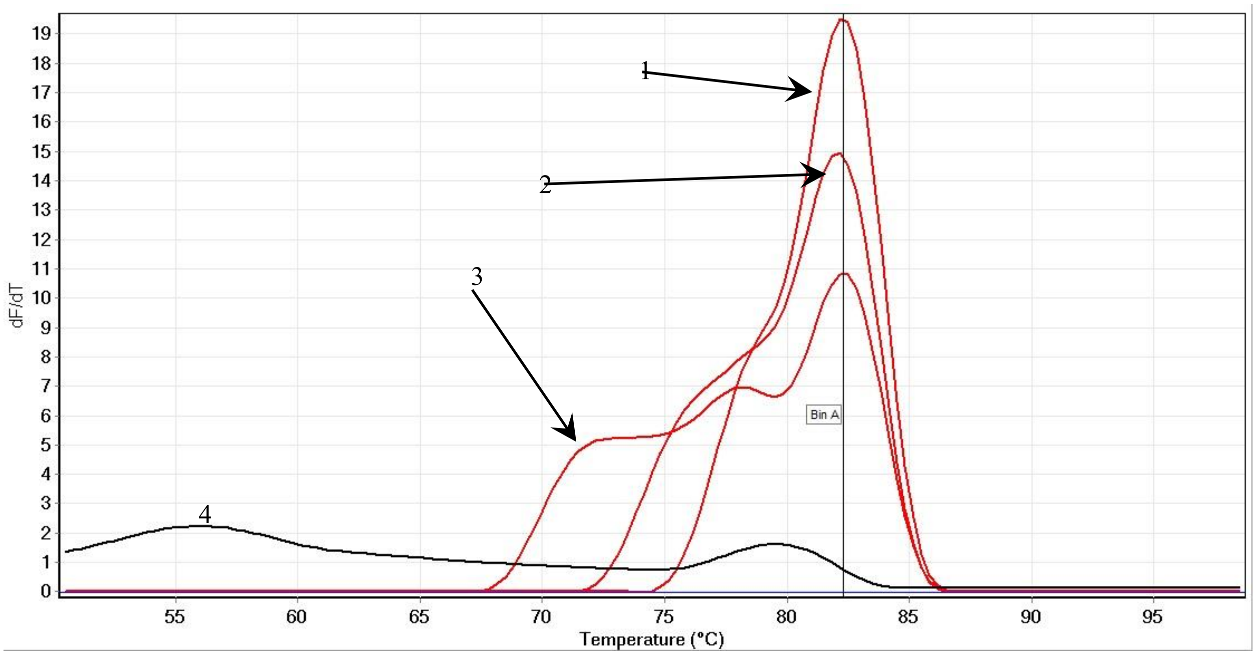

Figure 14: Melting curves of APX1B amplicones of strawberry DNA isolates obtained by kit 1 (Qiagen). 1-3 strawberry DNA isolates, 4 - no template control.

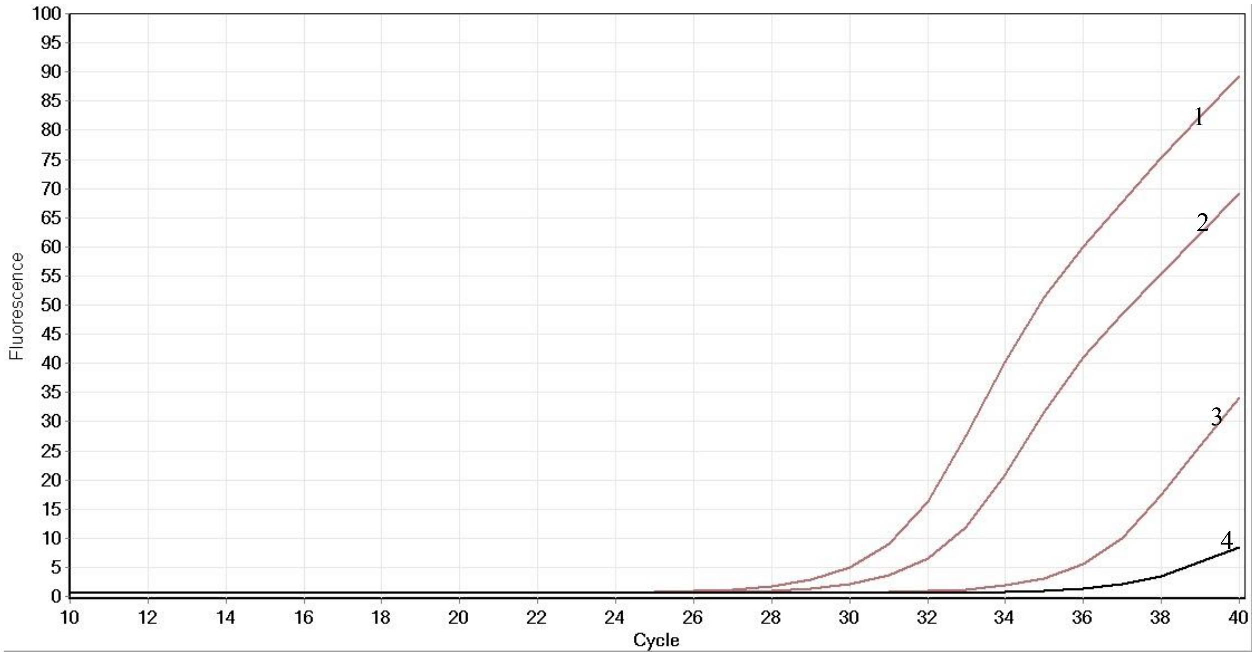

Figure 15: APX1B amplification curves of strawberry DNA isolates obtained by kit 2 (Elisabeth Pharmacon). 1-3 strawberry DNA isolates, 4 - no template control.

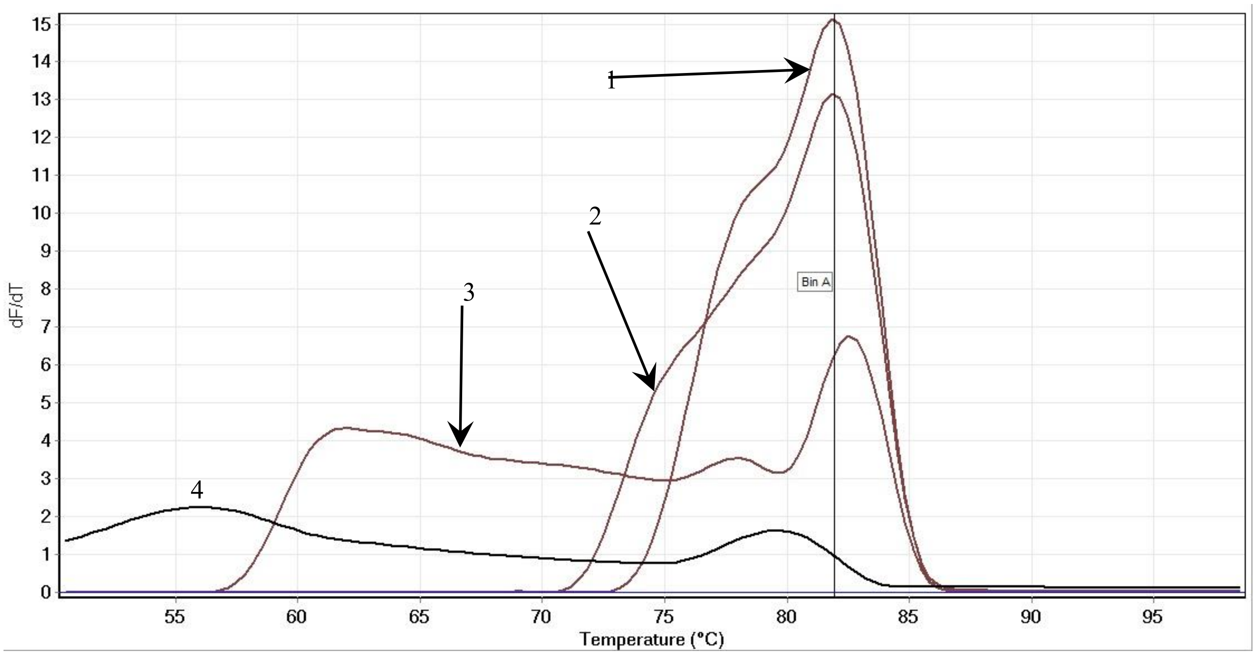

Figure 16: Melting curves of APX1B amplicones of strawberry DNA isolates obtained by kit 2 (Elisabeth Pharmacon). 1-3 strawberry DNA isolates, 4 - no template control.

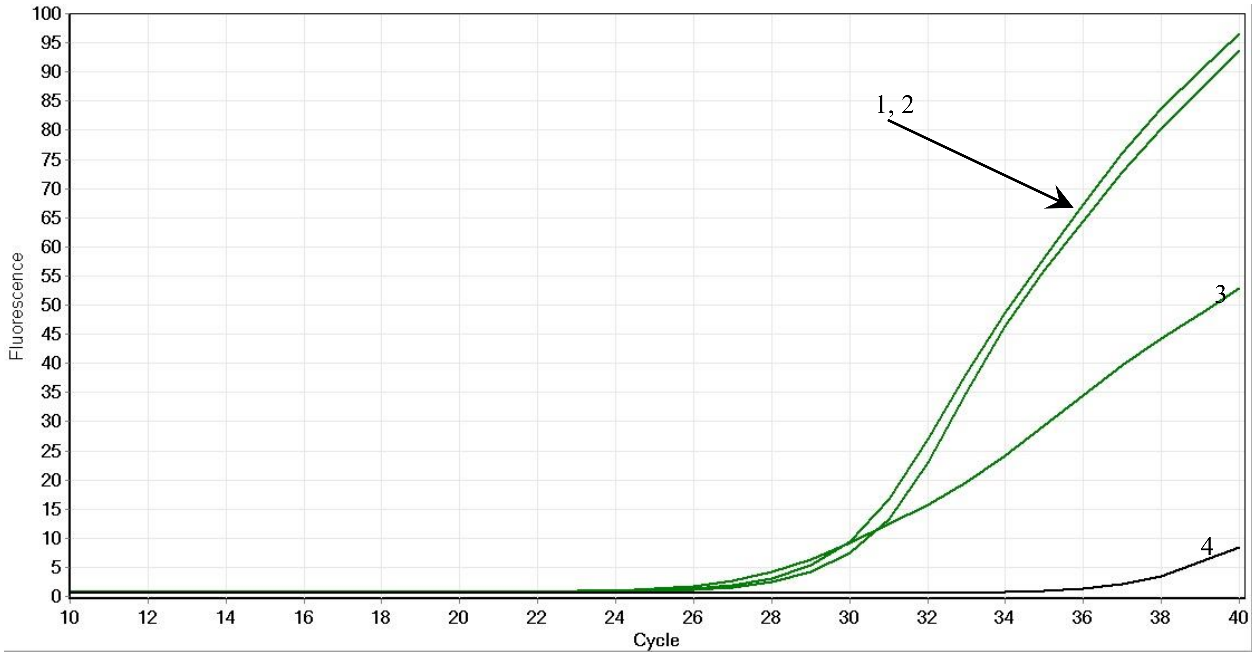

Figure 17: APX1B amplification curves of strawberry DNA isolates obtained by kit 3 (Invitrogen). 1-3 strawberry DNA isolates, 4 - no template control.

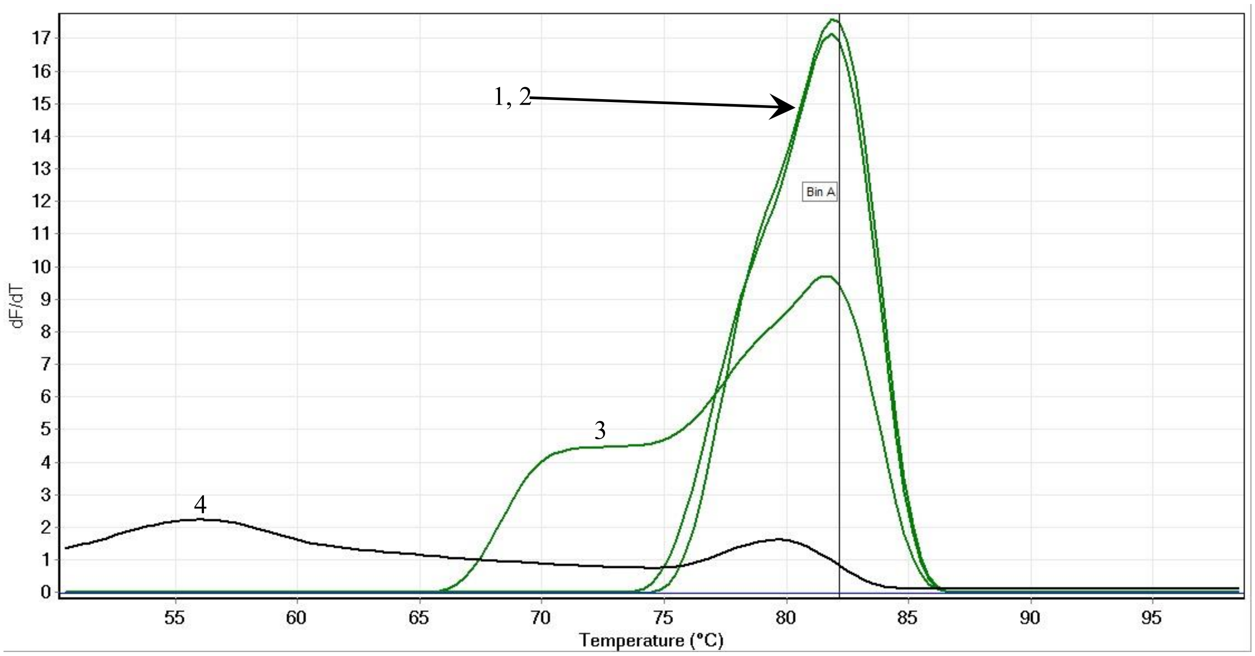

Figure 18: Melting curves of APX1B amplicones of strawberry DNA isolates obtained by kit 3 (Invitrogen). 1-3 strawberry DNA isolates, 4 - no template control.

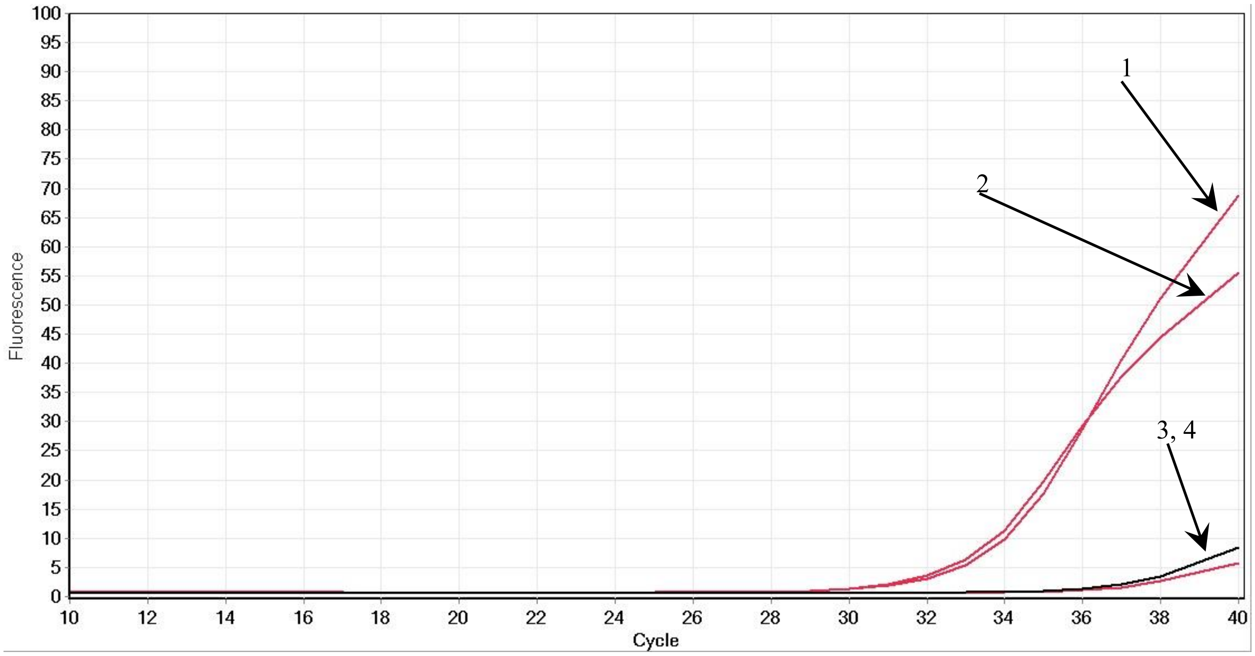

Figure 19: APX1B amplification curves of strawberry DNA isolates obtained by kit 4 (Perkin-Elmer). 1-3 strawberry DNA isolates, 4 - no template control.

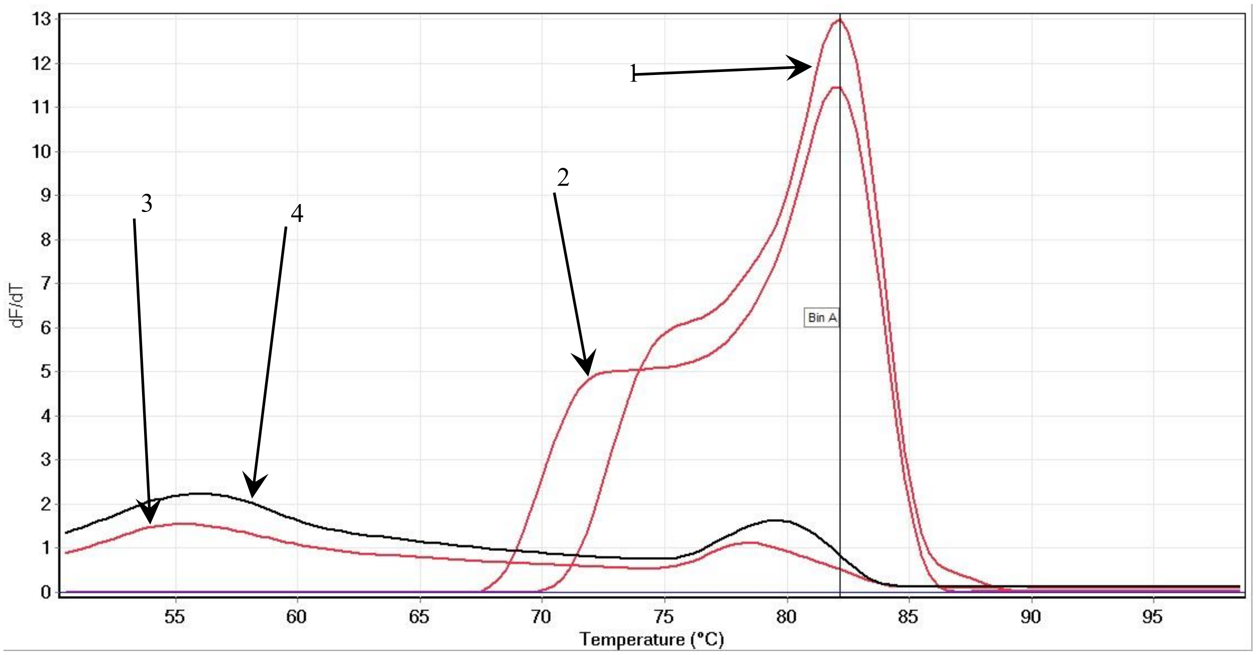

Figure 20: Melting curves of APX1B amplicones of strawberry DNA isolates obtained by kit 4 (Perkin-Elmer). 1-3 strawberry DNA isolates, 4 - no template control.

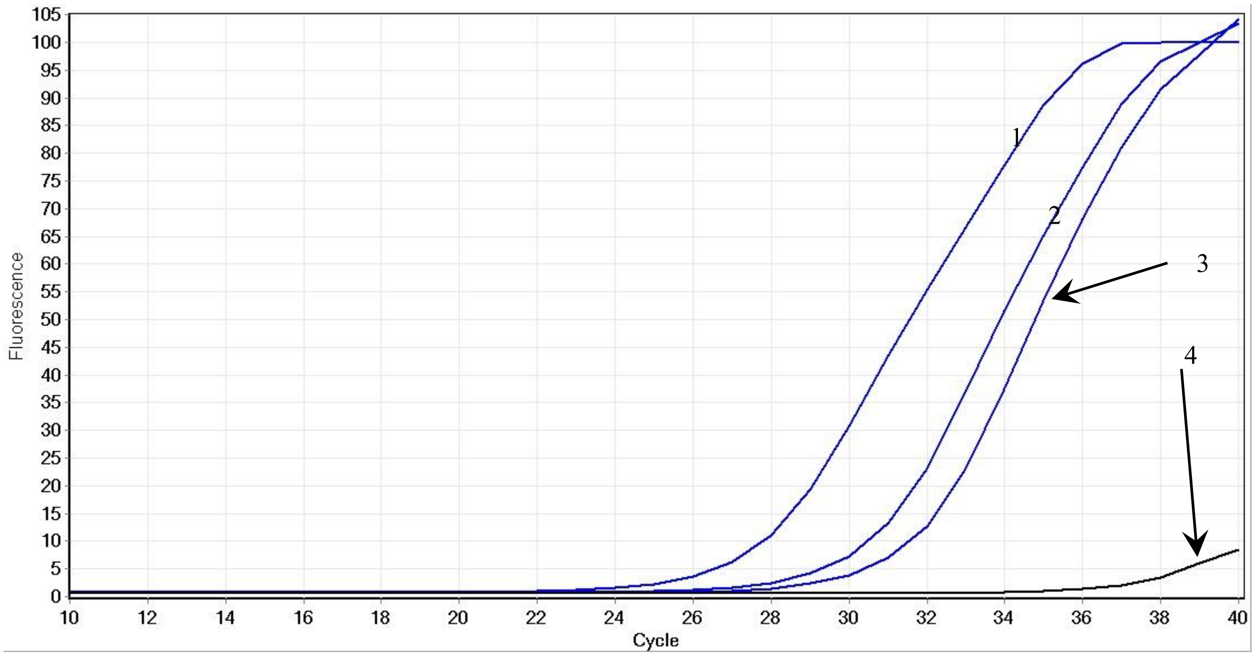

Figure 11: ITS2 amplification curves of strawberry DNA isolates obtained by the CTAB protocol. 1-3 strawberry DNA isolates, 4 - no template control.

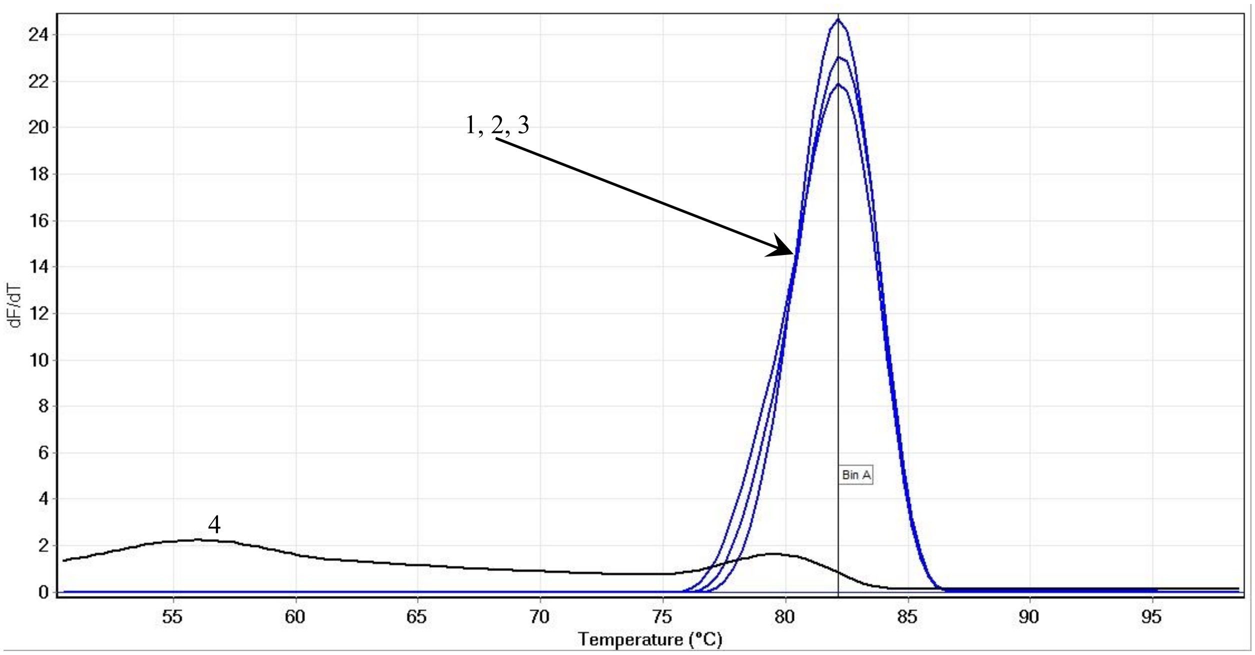

Figure 22: Melting curves of APX1B amplicones of strawberry DNA isolates obtained by kit 5 (Tools).
1-3 strawberry DNA isolates, 4 - no template control.

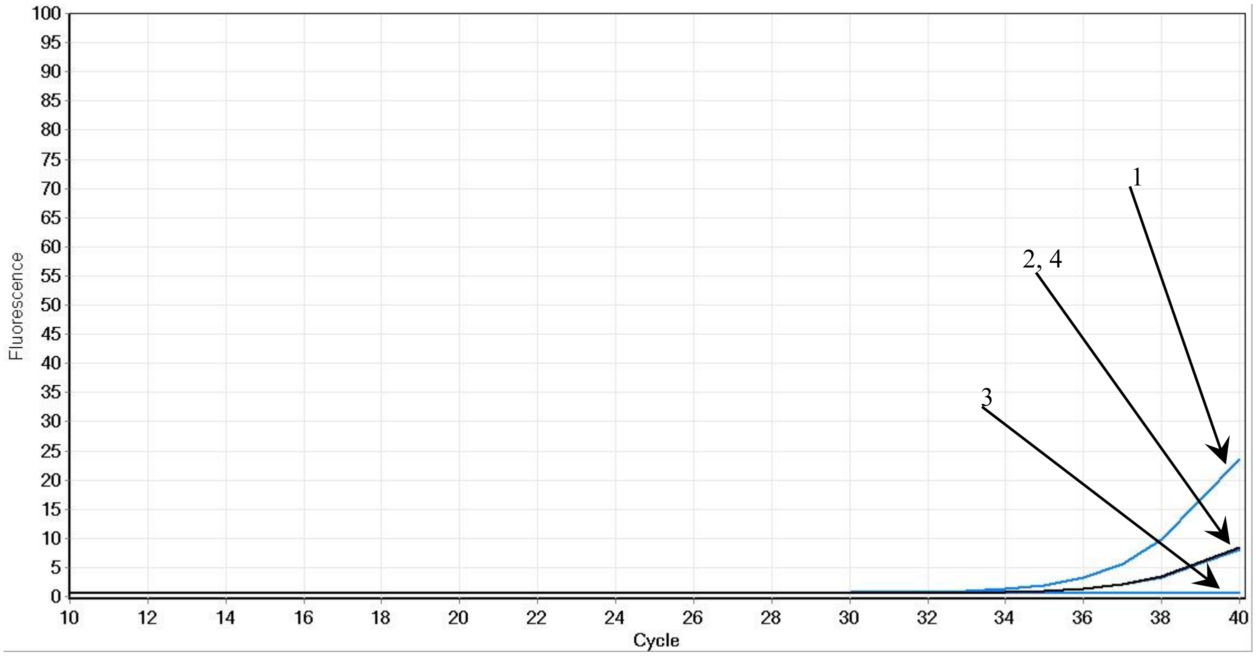

Figure 23: APX1B amplification curves of strawberry DNA isolates obtained by the CTAB protocol. 1-3 strawberry DNA isolates, 4 - no template control.

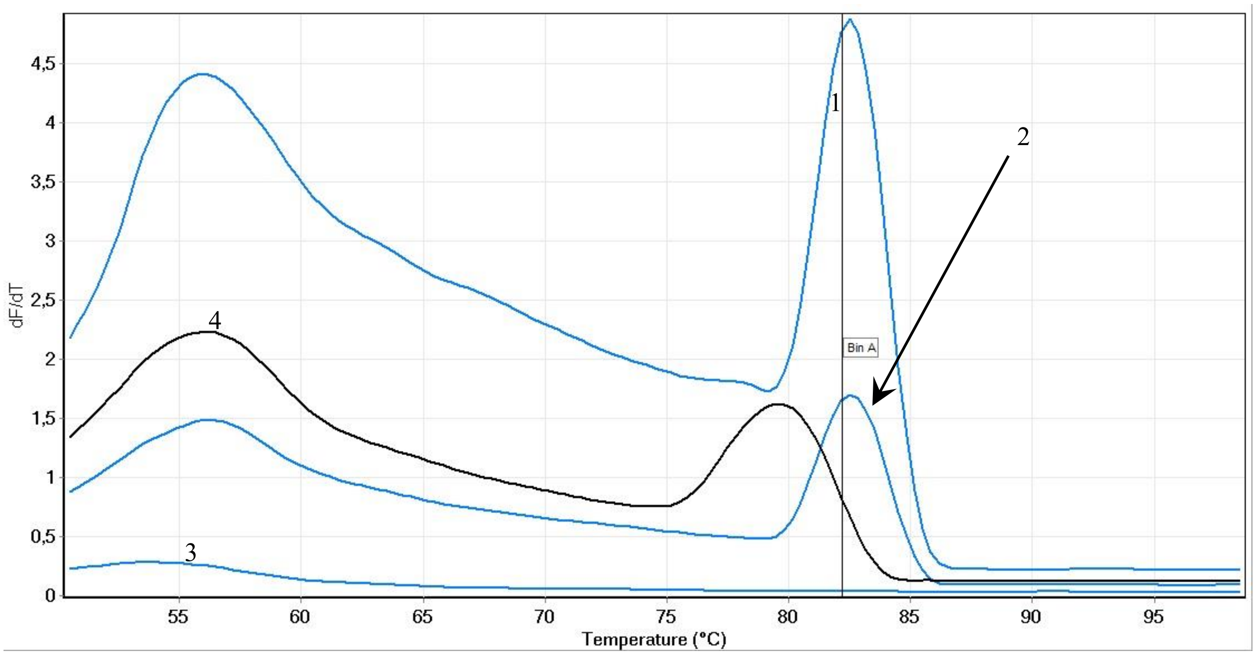

Figure 24: Melting curves of APX1B amplicones of strawberry DNA isolates obtained by the CTAB protocol. 1-3 strawberry DNA isolates, 4 - no template control.
