# Supplementary figures and images for: A Comparative Study of Some Procedures for Isolation of Fruit DNA of Sufficient Quality for PCR-Based Assays
Source: Molecules. 2020 Sep 20;25(18):4317. doi: 10.3390/molecules25184317 (PMC7570663; doi:10.3390/molecules25184317)

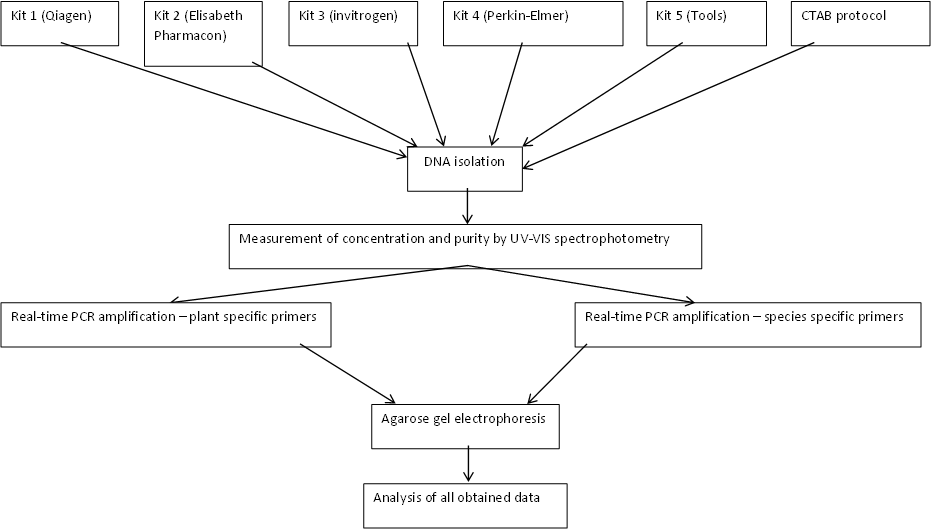

Supplement: Supplementary file 1 [file molecules-25-04317-s001.zip › molecules-913289-supplementary-revised-2nd - original/molecules 913289/S15 Workflow chart.tif]
